# Supplementary material for: Anisotropic flexibility and rigidification in a TPE-based Zr-MOFs with scu topology
Source: Nat Commun. 2023 Sep 2;14:5347. doi: 10.1038/s41467-023-41055-6 (PMC10475113; doi:10.1038/s41467-023-41055-6)
Supplement: Supplementary file 1 — Supplementary Information [file 41467_2023_41055_MOESM1_ESM.pdf]

Supplementary Information

Anisotropic Flexibility and Rigidification in a TPE-based Zr-MOFs with scu Topology

Sha-Sha Meng,[1,‡] Ming Xu,[1,‡] Hanxi Guan,[2,3,‡] Cailing Chen,[4] Peiyu Cai,[5] Bo Dong,[1] Wen-Shu Tan,[1] Yu-Hao Gu,[1] Wen-Qi Tang,[1] Lan-Gui Xie,[1] Shuai Yuan,[6] Yu Han,[4,7,8] Xueqian Kong,[2] and Zhi-Yuan Gu[1],*

[1]Jiangsu Key Laboratory of Biofunctional Materials, Jiangsu Collaborative Innovation Center of Biomedical Functional Materials, Jiangsu Key Laboratory of New Power Batteries, College of Chemistry and Materials Science, Nanjing Normal University, Nanjing 210023, E-mail: guzhiyuan@njnu.edu.cn

[2]Department of Chemistry, Zhejiang University, Hangzhou 310027, China

[3]Institute of Zhejiang University-Quzhou, Quzhou, 324100, China

[4]Advanced Membranes and Porous Materials Center, Physical Sciences and Engineering Division, King Abdullah University of Science and Technology, Thuwal, 23955-6900, Saudi Arabia

[5]Department of Chemistry, Texas A&M University, College Station, TX 77843-3255, United States

[6]State Key Laboratory of Coordination Chemistry, Key Laboratory of Mesoscopic Chemistry of MOE, School of Chemistry and Chemical Engineering, Nanjing University, Jiangsu 210023, China

[7]Electron Microscopy Center, South China University of Technology, Guangzhou 510640, China

[8]School of Emergent Soft Matter, South China University of Technology, Guangzhou 510640, China

[‡] These authors contributed equally to this work.

Supplementary Methods

Materials and Instruments

All chemicals employed were of analytical grade and used as supplied without further purification. The $ZrCl_4$, acetic acid, 3-aminopropyltriethoxysilane (APTES), anhydrous $AlCl_3$, carbon disulfide, anhydrous Na_2SO_4, dichloromethane, ethylene glycol, ethyltoluene, dichlorobenzene, C_6H_{14}, C_6H_{12}, C_7H_{16}, C_7H_{14}, C_8H_{18}, C_8H_{16}, and $C_{10}H_{22}$ were purchased from Aladdin Industrial Inc (Shanghai, China). The benzene-d_6, tetrahydrofuran (THF), and $TiCl_4$ were purchased from Energy Chemical (Anhui, China). Ethanol (EtOH), methanol (MeOH), N, N-Dimethylformamide (DMF) were purchased from Sinopharm Chemical Reagent Co., Ltd (Shanghai, China). The 1,1,2,2-tetra(4-carboxylphenyl)ethylene (H_4TCPE) was purchased from Chemsoon Co., Ltd (Shanghai, China). The fumaric acid (FA) and 2,6-naphthalenedicarboxylic acid (NDC) were purchased from TCI (Shanghai, China).

Powder X-ray diffraction (PXRD) patterns were obtained from a Rigaku SmartLab 9 Kw (Tokyo, Japan) diffractometer with a CuKα radiation (1.54056 Å). Transmission electron microscopy (TEM) images were performed on JEOL JEM-2100F transmission electron microscopy ope, rated at an accelerating voltage of 200 kV. Scanning electron microscope (SEM) images were collected on a JSM-7600F (JEOL Ltd) scanning electron microscope. Nuclear magnetic resonance (NMR) was recorded on a Bruker AN-400 MHz instrument. About 2 mg material was added into a 1.5 mL plastic tube, mixed with about 500 μL DMSO-d_6 and 2.5 μL D_2SO_4. Then, the tube was heated until all the solid was dissolved. The solution was transferred to the NMR tube and analyzed on an AN-400 MHz spectrometer. Thermogravimetric analysis (TGA) was collected on a Perkin-Elmer Pyris Diamond 1 TGA analyzer. The fluorescence spectroscopy was collected on a fluorescence spectrometer (Hitachi F-4600). Nitrogen sorption measurements were conducted on ASAP 2460 instrument. The high-angle annular dark field images (HAADF) were recorded on a Titan Themis Cubed G2 60-300 transmission electron microscope (FEI, US) with a probe corrector operated at 200 keV. All of the separations were performed on an Agilent 7890B gas chromatographic system with a flame ionization detector (FID). Data acquisition and processing were controlled by ChemStation software. Nitrogen (99.999%, Air Liquide, France)

was employed as the carrier gas. The inlet temperature of the GC was set to 250 °C, while the temperature of FID was set to 300 °C. A 2 μL analyte was introduced to a 20 mL gastight-sealed glass vial and homogenized at 100 °C before the injection for gas chromatographic separation.

Synthesis of Zr-TCPE-DLI

The Zr-TCPE-DLI was synthesized according to the previous report.[1] Typically, as-synthesized Zr-TCPE (about 15 mg) was soaked in DMF (2 mL) with the addition of linker L_2 (0.03 M, 4.6 mL DMF). The mixture was stirred at 75 ºC for 24 h. The product was collected by centrifugation and washed with DMF three times and dispersed in 2 mL DMF, then stirred with the solution of linker L_1 (0.03 M, 4.6 mL DMF) at 75 ºC for 24 h. The product was collected by centrifugation and soaked in fresh DMF for 3 days to remove uncoordinated linkers.

Synthesis of Zr-TCPE-DLI'

The Zr-TCPE-DLI' was synthesized according to the previous report.[1] Typically, as-synthesized Zr-TCPE (about 15 mg) was soaked in DMF (2 mL) with the addition of linker L_1 (0.03 M, 4.6 mL DMF). The mixture was stirred at 75 ºC for 24 h. The product was collected by centrifugation and washed with DMF three times and dispersed in 2 mL DMF, then stirred with the solution of linker L_2 (0.03 M, 4.6 mL DMF) at 75 ºC for 24 h. The product was collected by centrifugation and soaked in fresh DMF for 3 days to remove uncoordinated linkers.

Method 1 of restoration from Zr-TCPE-H to Zr-TCPE

The 10 mg Zr-TCPE-H was dispersed in 2 mL DMF in a glass vial with the addition of 0/200/400 μL AA. Then the vial was heated at 120 °C for 12 h in an oven. After cooling to room temperature, the product was collected by configuration and washed with DMF and EtOH three times, respectively, before drying under vacuum.

Method 2 of restoration from Zr-TCPE-H to Zr-TCPE

The 10 mg Zr-TCPE-H was dispersed in 2 mL DMF in a glass vial with the addition of 200/400 μL AA. Then the vial was heated at 100 °C for 12 h in an oven. After cooling to room temperature, the product was collected by configuration and washed with DMF and EtOH three times, respectively, before drying under vacuum.

Method 3 of restoration from Zr-TCPE-H to Zr-TCPE

The 10 mg Zr-TCPE-H was dispersed in 2 mL DMF in a glass vial with the addition of 200/400 μL AA. Then the vial was kept at room temperature for 12 h. The product was collected by configuration and washed with DMF and EtOH three times, respectively, before drying under vacuum.

Rietveld Refinement

High-quality PXRD data for Rietveld refinement was collected on Rigaku SmartLab 9 Kw (Tokyo, Japan) diffractometer with a CuKα radiation (1.54056 Å, angle range: 4-110°, step size: 0.01°, IS=1/4, RS1=5 mm, room temperature). All the processes of refinements were performed on the TOPAS 64 V6.

Refinement details of Zr-TCPE

A structural model of Zr-TCPE was developed starting from NU-901 structure.[2] Indexing of the PXRD data of Zr-TCPE suggested orthorhombic space groups. Assuming the inorganic building unit to be the hexanuclear cluster most frequently observed in Zr-MOFs, a structure model starting from the NU-901 was set up. The first step was replacing the ligand from TBAPy in NU-901 to TCPE and adjusting the original lattice parameters to the ones obtained by indexing. Molecular mechanics optimizations of the atom positions within the unit cell were performed to reach energy minimization using the Universal force field (UFF) in Material Studio 7.0. Interpenetration was not possible in this case due to limited lattice void space. Pawley refinement (Reflex module of Materials Studio) against the full powder pattern using profile fitting, FWHM, and asymmetry correction parameters yields cell parameters a = 17.724(46) Å, b = 30.182(02) Å, and c = 12.292(77) Å (residuals: R_p = 4.40%, R_{wp} = 6.41%). The low residuals indicated the refined profile matched the experimental XRD pattern very well. To examine the model, Rietveld refinement was also performed on the TOPAS 64 V6.

Low-dose HRTEM Measurements

Low-dose HRTEM experiments were performed on a Cs-corrected FEI cubed G2 Titan 60-300 electron microscope at an acceleration voltage of 300 kV, using a Gatan K2 direct-detection camera in the electron-counting mode with the dose fractionation function. The ED pattern was simulated by using Single Crystal Software.

Capillary Pretreatment

A fused silica capillary (15 m long × 0.25 mm i.d., Yongnian Optic Fiber Plant, Hebei, China) was pre-treated according to the following process before dynamic coating: the capillary was washed sequentially with 1 mol/L NaOH, ultrapure water, 0.1 mol/L HCl, ultrapure water again until the outflow reached pH=7.0, and finally MeOH. The pretreated capillary was filled with a 1:1 (v/v) mixture of MeOH and APTES and then incubated in a 40 ^{o}C water bath overnight with both ends of the capillary sealed. The APTES-modified capillary was rinsed with MeOH to flush out the residuals and dried with a stream of nitrogen at 120 ^{o}C.

Coating the Materials

Different materials were coated on the pretreated capillary columns by the dynamic coating method as follows: 1 mL methanol suspension of each material (2 mg/mL) was first filled into the insulin syringe. The insulin syringe was connected to the capillary column. Then the MOF suspension was pushed through the column by a syringe pump at a velocity of 4.5 mL/min to leave a wet coating layer on the inner wall of the capillary column. After coating, the capillary column was settled for conditioning under nitrogen to remove the solvent. Further conditioning of the capillary column was carried out using a temperature program: maintain 30 ^{o}C for 30 min, then ramp to 250 ^{o}C at a rate of 2 ^{o}C/min and keep at 250 ^{o}C for 240 min.

Calculation of Thermodynamic Parameters

The adsorption enthalpy (*ΔH*) was calculated from the van’t Hoff equation (1):

$$lnk' = -\frac{\Delta H}{RT} + \frac{\Delta S}{R} + ln\emptyset \quad (1)$$

Here, *k’* is the retention factor, *R* is the gas constant (*R*= 8.314 J·mol^{-1}·K^{-1}), *T* is the absolute temperature, and *Ø* is the phase ratio (the ratio of the volume of the stationary phase (V_s) to that of the mobile phase (V_m)). To obtain *Ø*, V_s was calculated from the thickness of MOFs coated on the capillary column, while V_m was calculated from the column internal volume subtracting V_s.

k’ is calculated from equation (2):

$$k' = \frac{t-t_0}{t_0} \quad (2)$$

t is the retention time of the analyte, and t_0 is the retention time of an unretained compound on the column.

Calculation of Kinetic Parameters

The kinetic diffusion constant (D_s) was calculated from the Golay equation:

$$H = \frac{2D_g}{u} + \frac{1+6k+11k^2}{24(1+k)^2} \times \frac{r^2}{D_g} \times u + \frac{2}{3} \times \frac{k}{(1+k)^2} \times \frac{d_f^2}{D_s} \times u \quad (3)$$

Here, D_g is the diffusion constant of the analyte in the gas phase, D_s is the diffusion constant of the analyte in the stationary phase, *r* is the radius of the capillary column (*r*=125 mm), *d* is the thickness of the stationary phase (*d*=100 nm), *u* is the linear velocity of the carrier gas, *H* is the height equivalent of the theoretical plate.

The resistance to mass transfer coefficient (C_m) is calculated according to equation (4):

$$C_m = \frac{2}{3} \times \frac{k}{(1+k)^2} \times \frac{d_f^2}{D_s} \quad (4)$$

H is calculated according to equation (5):

$$H = \frac{L}{16(\frac{t}{w})^2} \quad (5)$$

L was the length of the capillary column (*L*=15 m). *w* represents the full width of the analyte.

Supplementary Figures

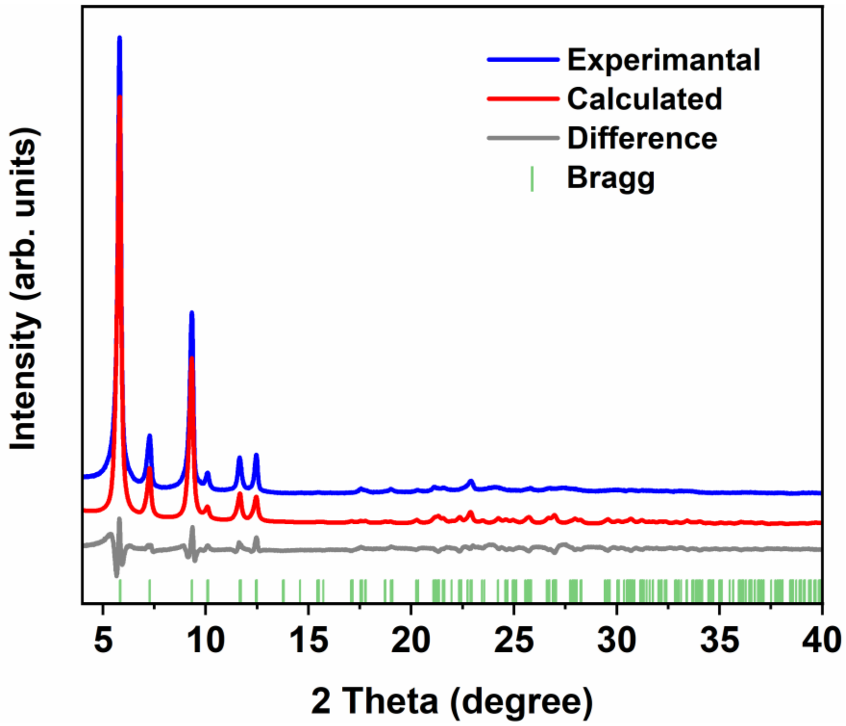

Supplementary Figure 1. Final Rietveld refinement plots of Zr-TCPE. The experimental, calculated, and difference curves are in blue, red, and gray, respectively. The vertical bars indicate the positions of the Bragg peaks.

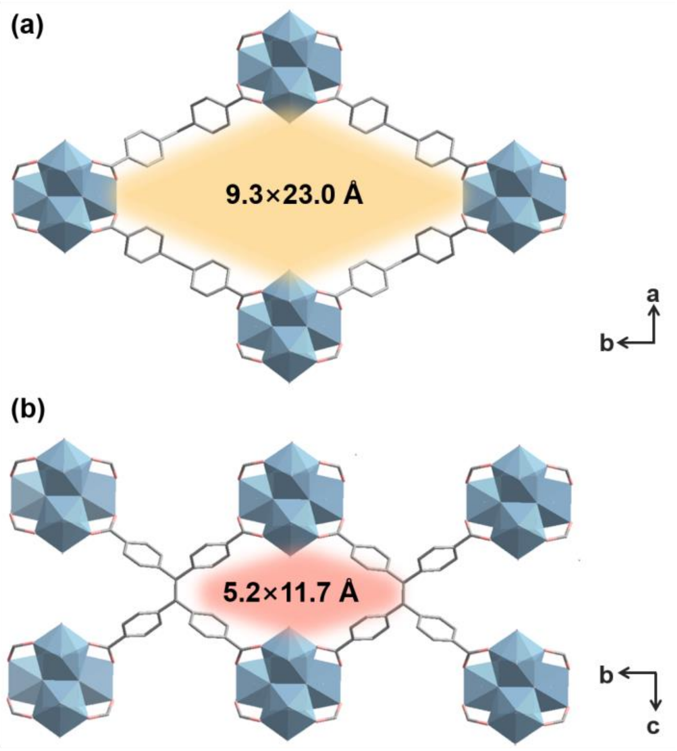

Supplementary Figure 2. The rhombic channels in Zr-TCPE along the (a) [001] direction and (b) the [100] direction. Zr, blue; C, grey; O, red. Hydrogen atoms were omitted for clarity.

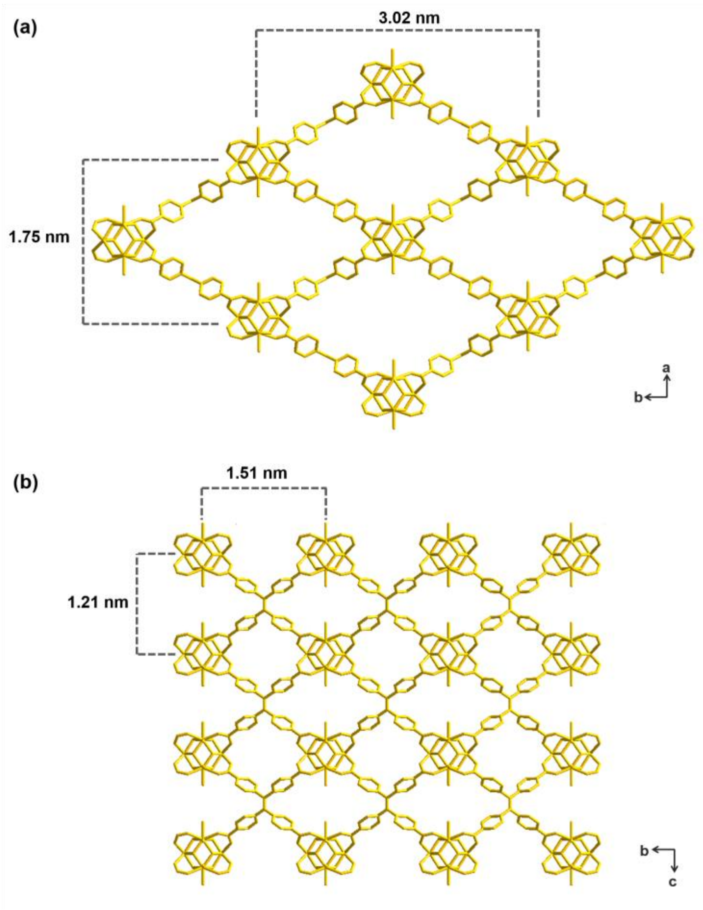

Supplementary Figure 3. The simulated structure of non-interpenetrated Zr-TCPE along (a) the [001] direction and (b) the [100] direction.

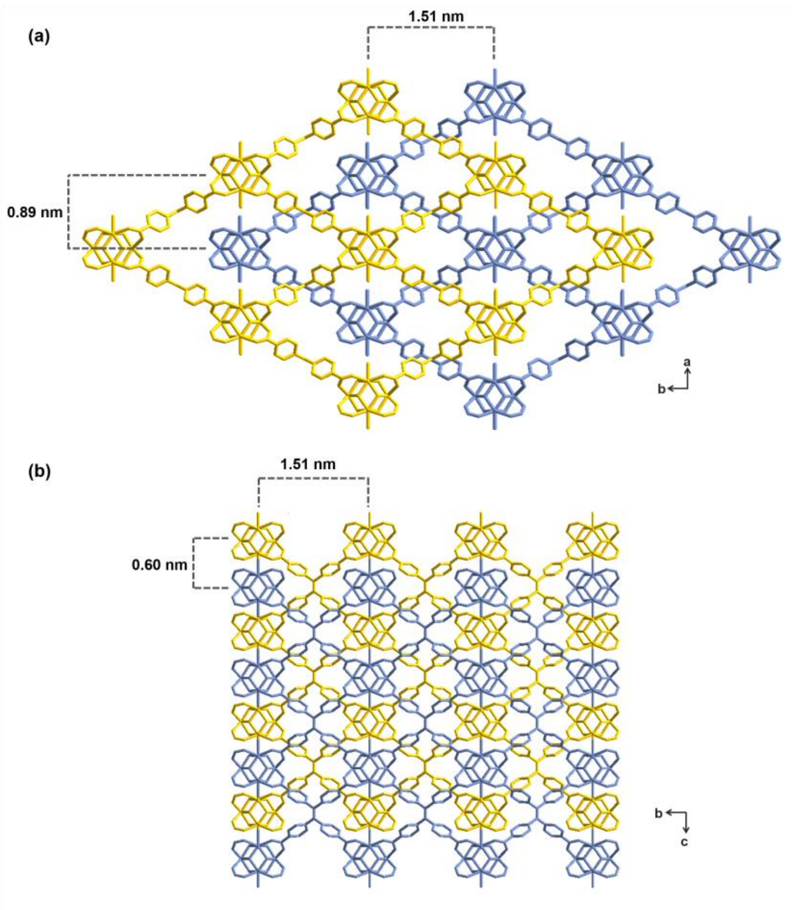

Supplementary Figure 4. The simulated structure of interpenetrated Zr-TCPE along (a) the [001] direction and (b) the [100] direction.

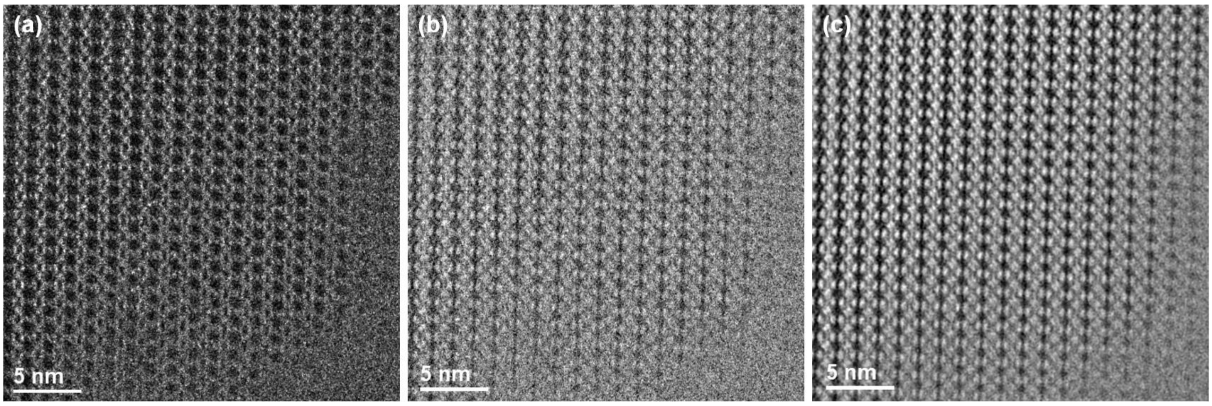

Supplementary Figure 5. (a) The raw low-dose HRTEM image of Zr-TCPE. The black dots in the HRTEM images represented the Zr_6 clusters. (b) CFT-corrected image. (c) ABSF-filtered CTF-corrected image.

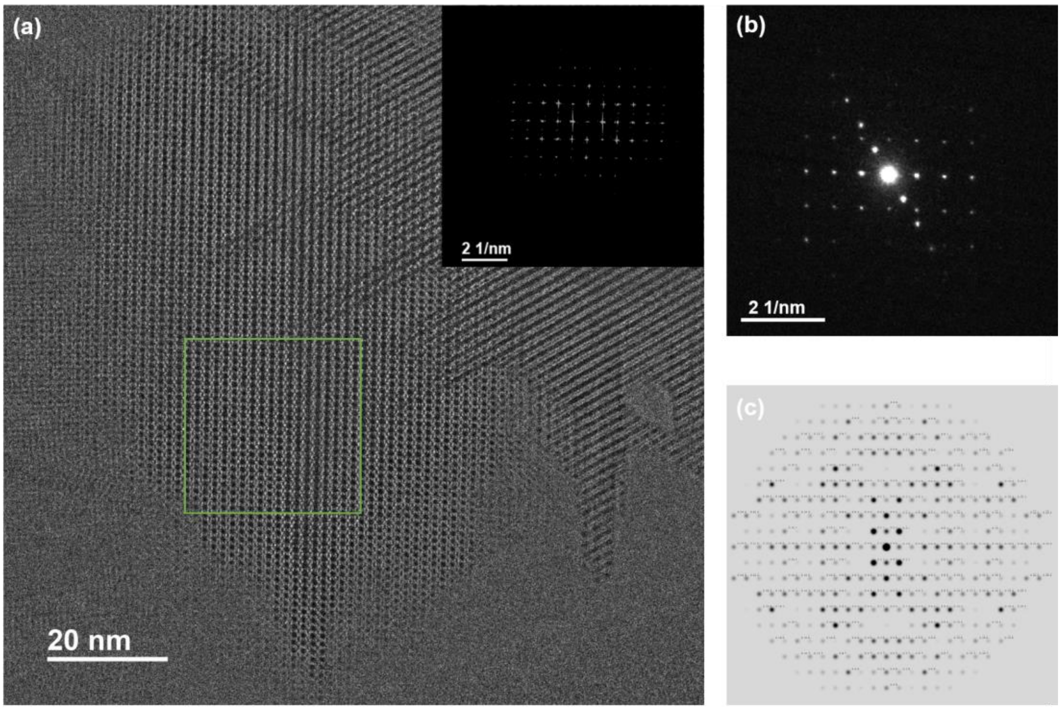

Supplementary Figure 6. (a) The low-dose HRTEM image of Zr-TCPE along the [100] direction. The black dots in the HRTEM images represented the Zr_6 clusters. The insert in the FFT pattern of the marked region. (b) The SAED pattern of Zr-TCPE. (c) Simulated electron diffraction pattern along the [100] direction.

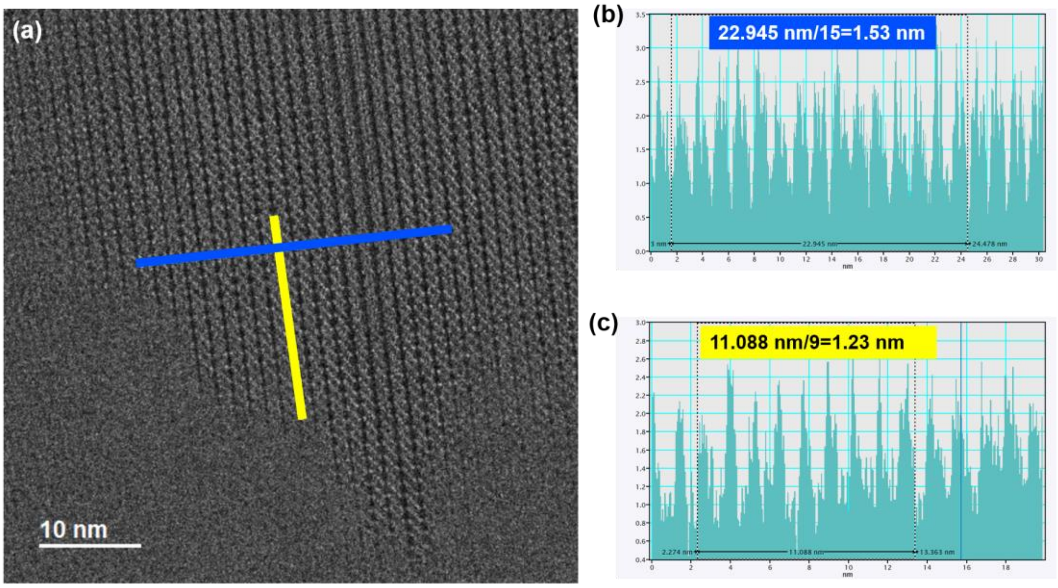

Supplementary Figure 7. (a) The raw low-dose HRTEM image of Zr-TCPE along the [100] direction. The black dots in the HRTEM images represented the Zr_6 clusters. (b,c) The measured distance of adjacent Zr_6 clusters was along the b-axis (labeled in blue) and the c-axis (labeled in yellow). The distance of adjacent Zr_6 clusters along the b-axis and c- axis was around 1.53 nm and 1.23 nm, which was comparable to the simulated value (1.51 nm and 1.21 nm shown in Supplementary Fig. 3).

On the one hand, the structure of Zr-TCPE was analyzed by Rietveld refinement. The refinement results revealed that each TCPE ligand was connected to four Zr_6 clusters and each Zr_6 cluster was coordinated with eight TCPE ligands. Then, the non-interpenetrated scu coordination structure was generated in Zr-TCPE. On the other hand, the low-dose HRTEM image acquired along the [100] direction further confirmed the non-interpenetrated structure. The arrangement of Zr_6 clusters on the ABSF-filtered CTF-corrected image was consistent with the simulated non-interpenetrated structure of Zr-TCPE along the [100] direction. The calculated distances of adjacent Zr_6 clusters were also comparable to the simulated values in non-interpenetrated Zr-TCPE along the [100] direction. Besides, the calculated d-spacing of (020), (001), and (021) planes from the FFT pattern was similar to the simulated value. All the results revealed the non-interpenetrated structure of Zr-TCPE.

We can reasonably speculate that if Zr-TCPE possesses the interpenetrated structure, the distance of adjacent Zr_6 clusters along the c-axis will be half of the c-axis cell parameter (Supplementary Fig. 4). This hypothesis is inconsistent with the actual experimental value in HRTEM images. The distance of adjacent Zr_6 clusters along the c-axis calculated from the HRTEM image is 1.25 nm, which is consistent with the value in simulated non-interpenetrated Zr-TCPE (1.21 nm). Thus, non-interpenetrated structure not interpenetrated one is assigned to Zr-TCPE.

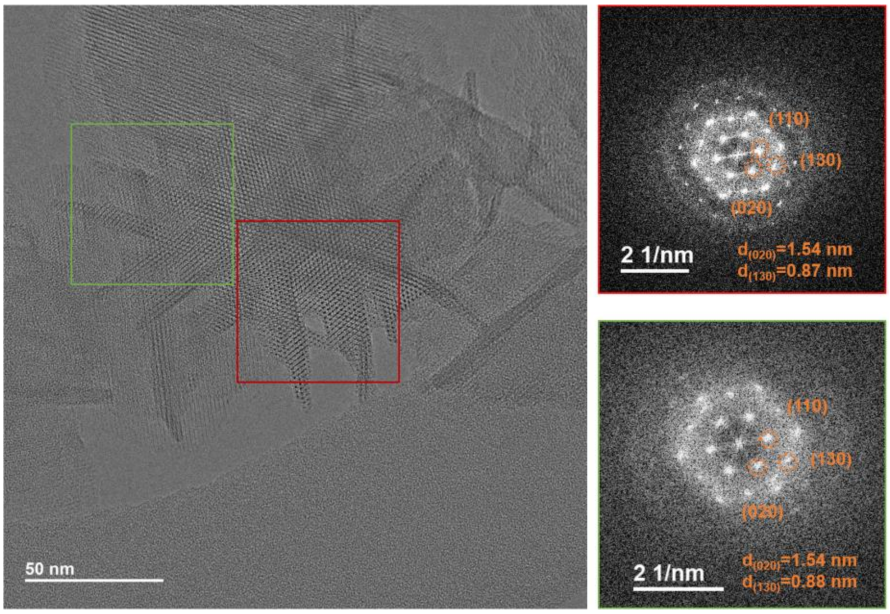

Supplementary Figure 8. The HRTEM image of Zr-TCPE with FFT patterns of marked areas. The calculated d-spacing of (020) and (130) planes were consistent with the simulated values ($d_{(020)}$=1.51 nm, $d_{(130)}$=0.87 nm).

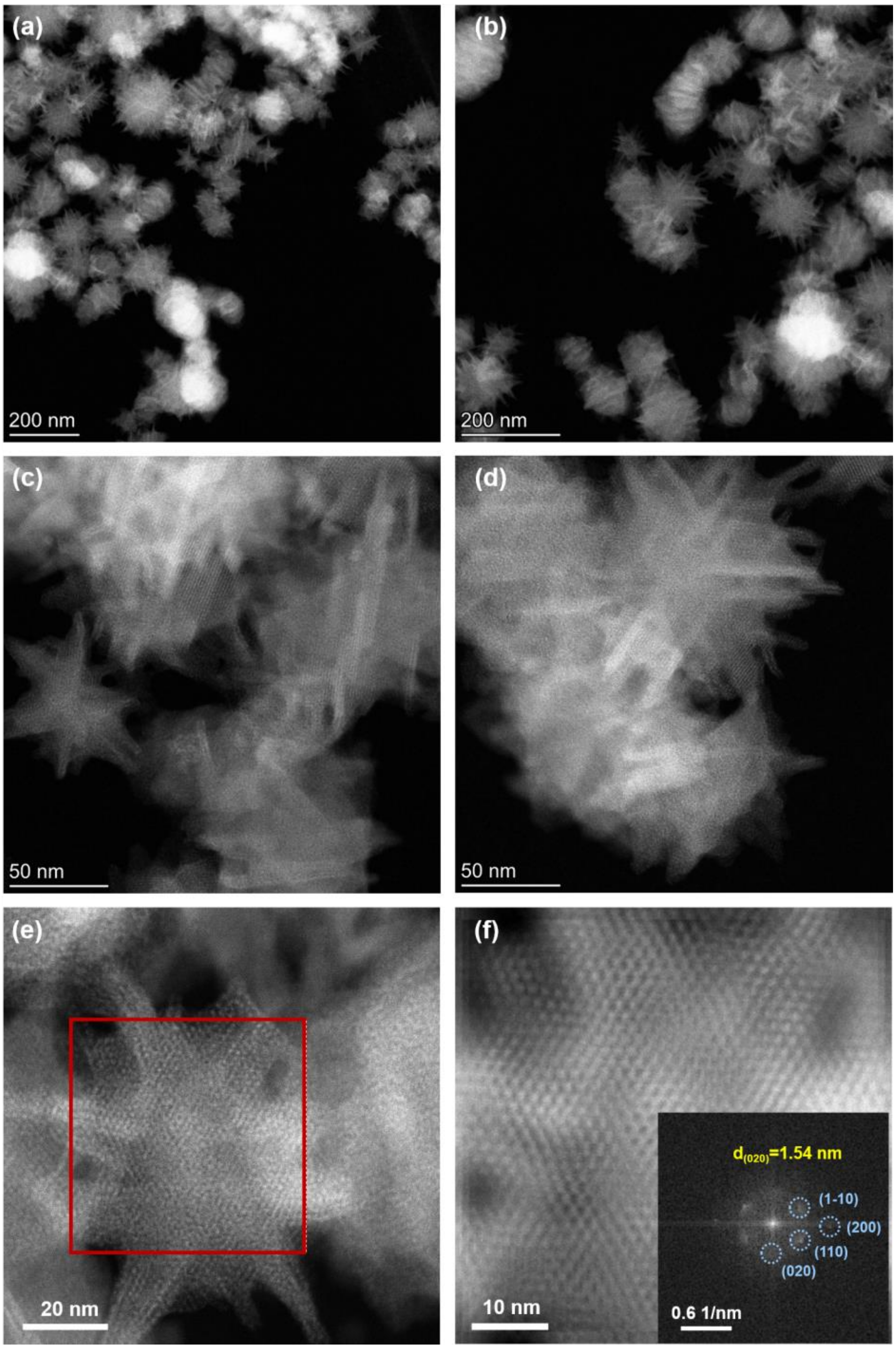

Supplementary Figure 9. (a-e) The HAADF images of Zr-TCPE. The white dots in the HAADF images represented the Zr_6 clusters. (f) The denoised HAADF image of the marked region in (e) and the FFT pattern is presented in the bottom right corner. The denoised image was acquired by an inverse-FFT process after applying a periodic mask to the FFT pattern. The d-spacing of the (020) plane was measured around 1.54 nm, which was comparable to the simulated value from PXRD refinement (d=1.51 nm).

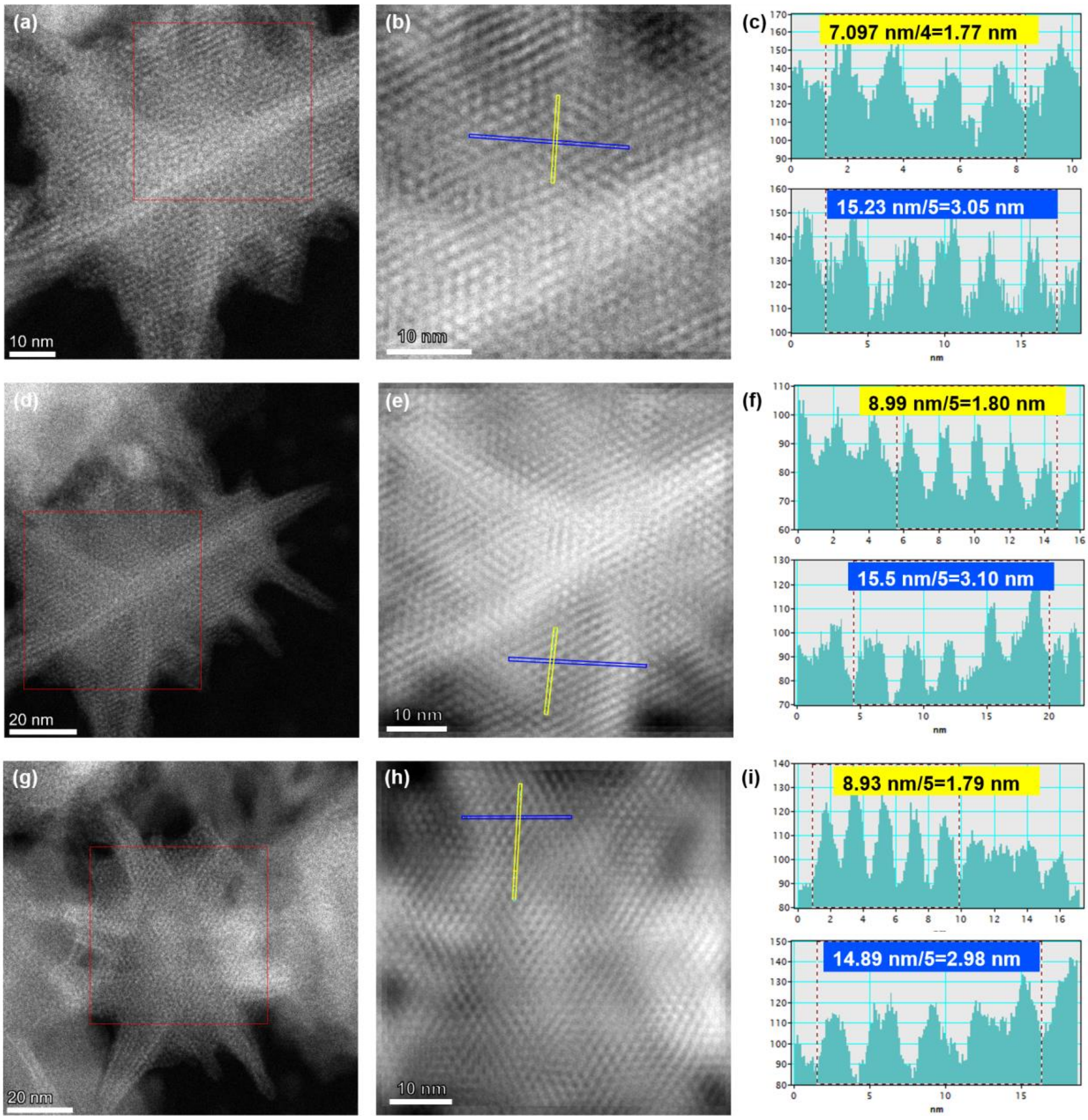

Supplementary Figure 10. (a,d,g) The HAADF images of Zr-TCPE. The white dots in the HAADF images represented the Zr_6 clusters. (b,e,h) The denoised HAADF images of the red square region. The denoised image was acquired by an inverse-FFT process after applying a periodic mask to the FFT pattern. (c,f, i) The measured distance of adjacent Zr_6 clusters was along the a-axis (labeled in yellow) and the b-axis (labeled in blue). The distance of adjacent Zr_6 clusters along the a-axis was around 1.79 nm, which was similar to the simulated value (d=1.75 nm). The distance of adjacent Zr_6 clusters along the b-axis was around 3.04 nm, which was similar to the simulated value (d=3.02 nm).

The distance of adjacent Zr_6 clusters was 1.75 nm along the a-axis in the simulated non-interpenetrated structure, which was consistent with the HAADF image measurements (d=1.79 nm). However, the distance of adjacent Zr_6 clusters in a double-interpenetrated

structure would be 0.89 nm (Supplementary Fig. 4), which was not found in any of the HAADF image measurements. Meanwhile, the distance of adjacent Zr_6 clusters was 3.02 nm along the b-axis in the simulated non-interpenetrated structure, which was also consistent with the HAADF image measurements (d=3.04 nm). Therefore, we assigned a non-interpenetrated structure to Zr-TCPE.

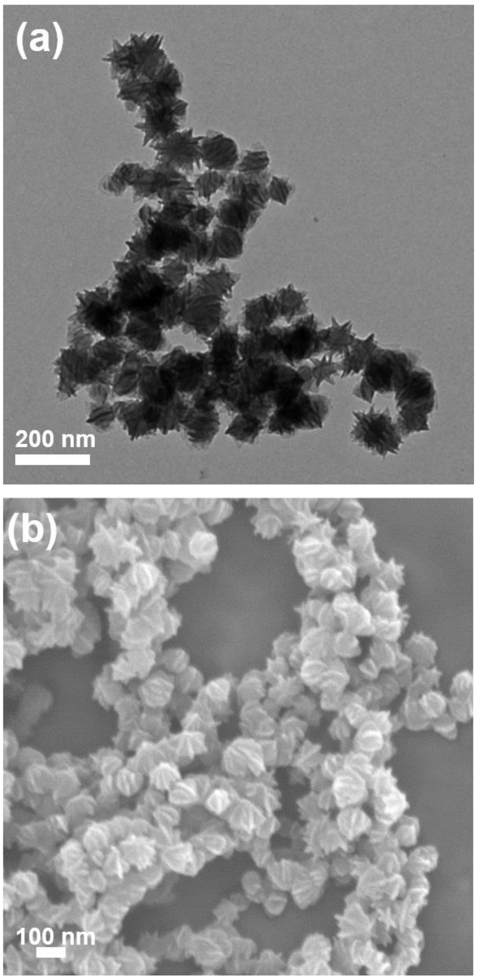

Supplementary Figure 11. (a) TEM and (b) SEM images of Zr-TCPE.

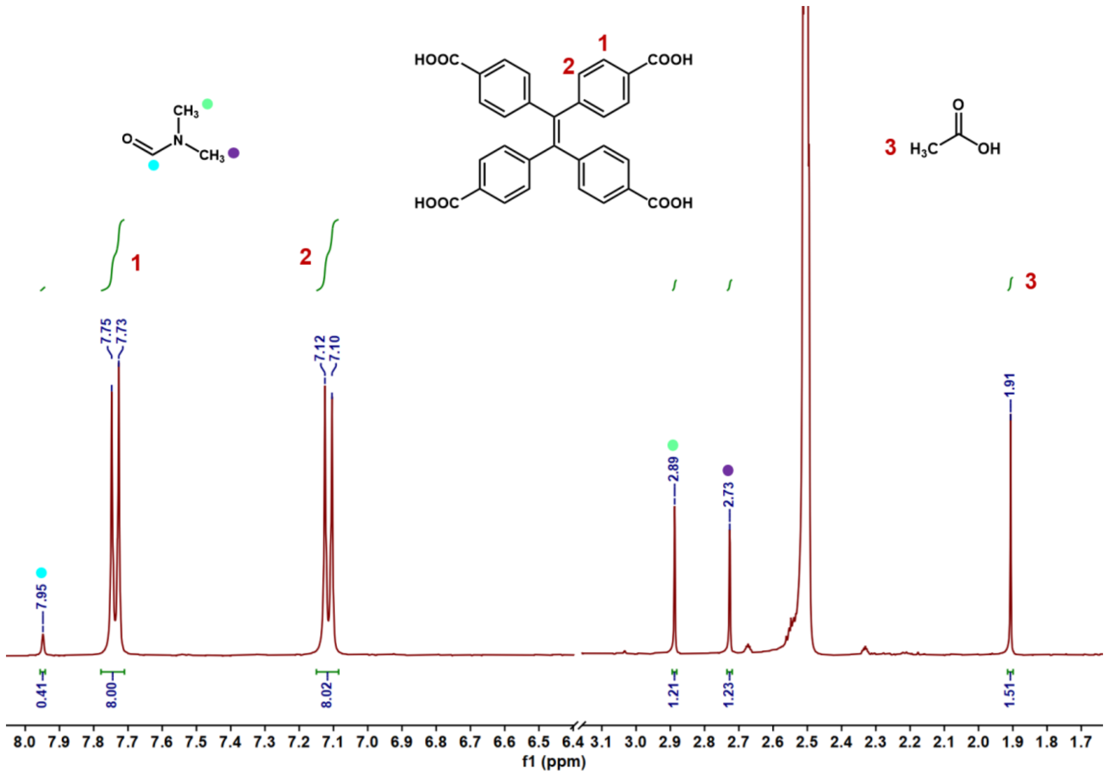

Supplementary Figure 12. The ^{1}H NMR spectroscopy of digested Zr-TCPE (TCPE: AA: DMF = 1: 0.5: 0.4).

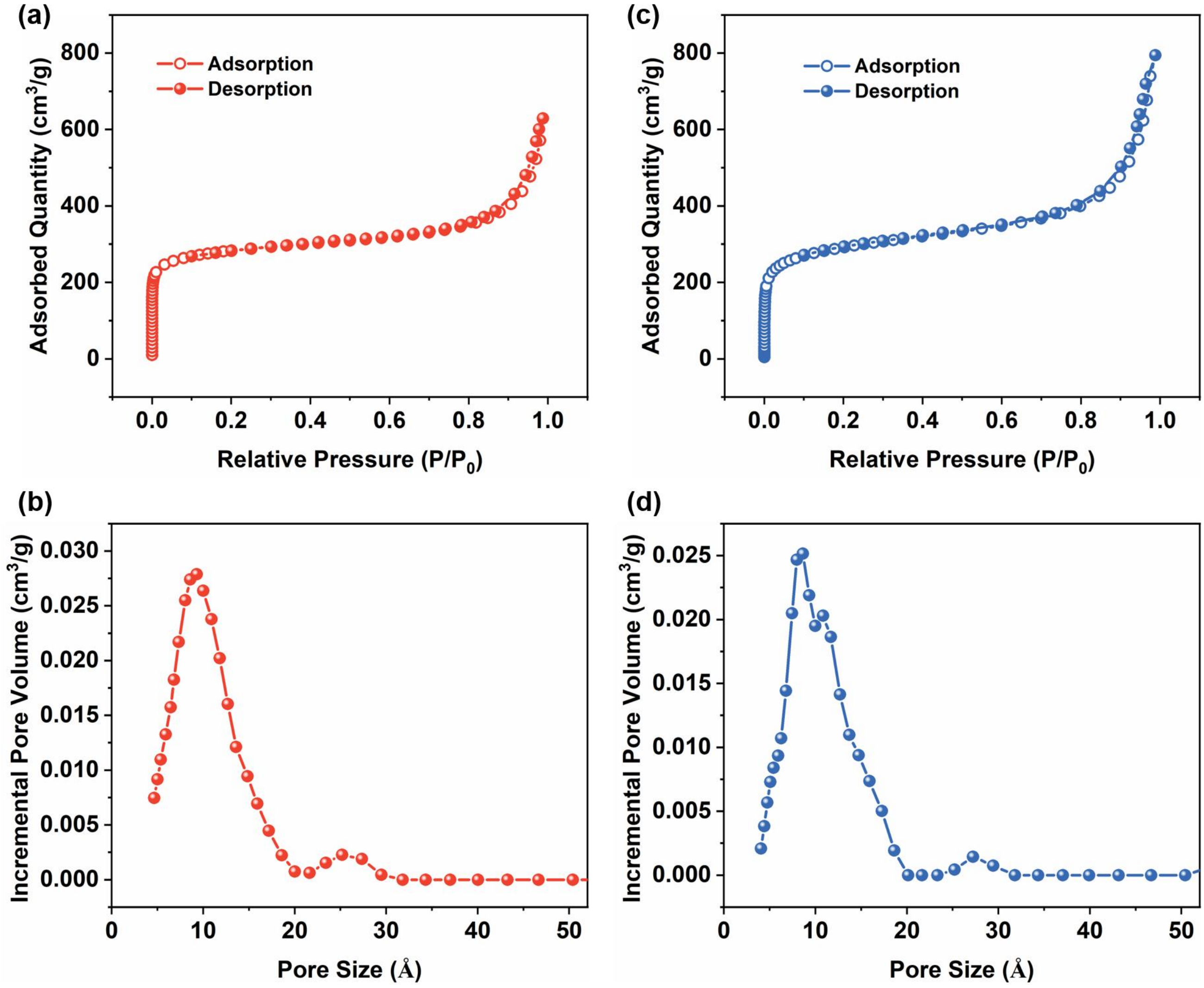

Supplementary Figure 13. (a) The N_2 adsorption-desorption isotherms and (b) pore size distribution of Zr-TCPE measured at 77 K. (c) The Ar adsorption-desorption isotherms and (d) pore size distribution of Zr-TCPE measured at 87K.

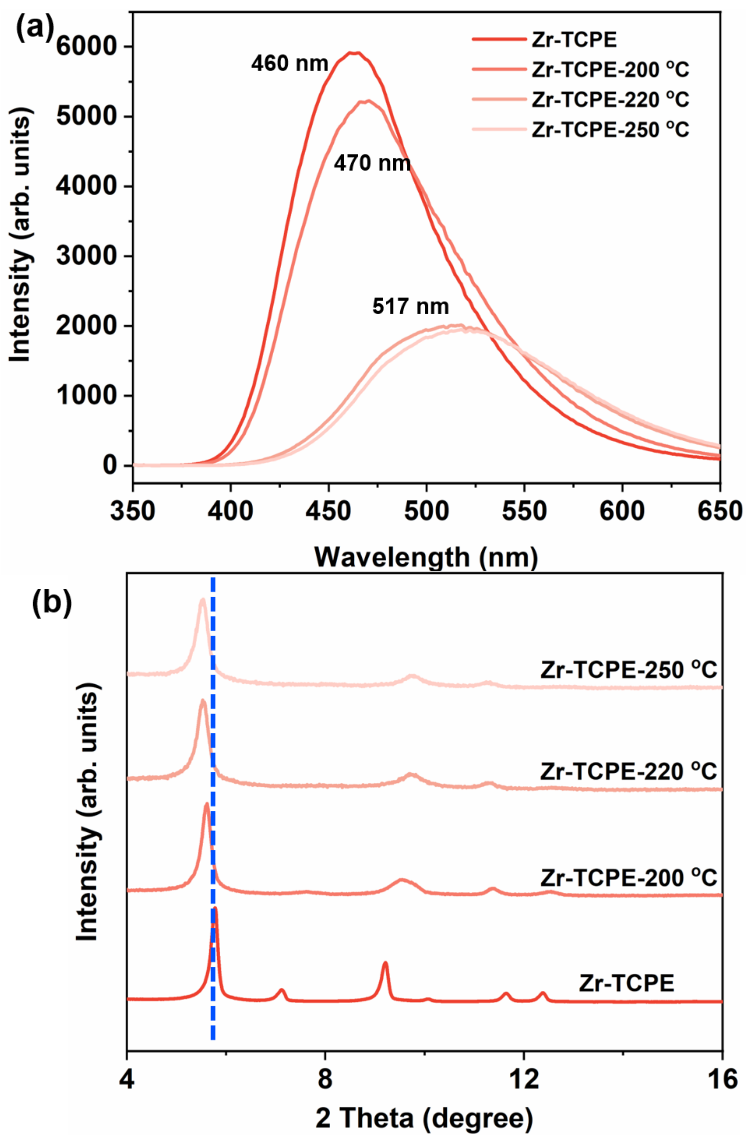

Supplementary Figure 14. (a) The fluorescence spectra of Zr-TCPE and heated materials dispersed in methanol solution at the concentration of 40 μg/mL (λ_{ex}=340 nm). (b) The PXRD patterns of Zr-TCPE and heated materials at 200 °C, 220 °C, and 250 °C (Zr-TCPE-H) for 6 h.

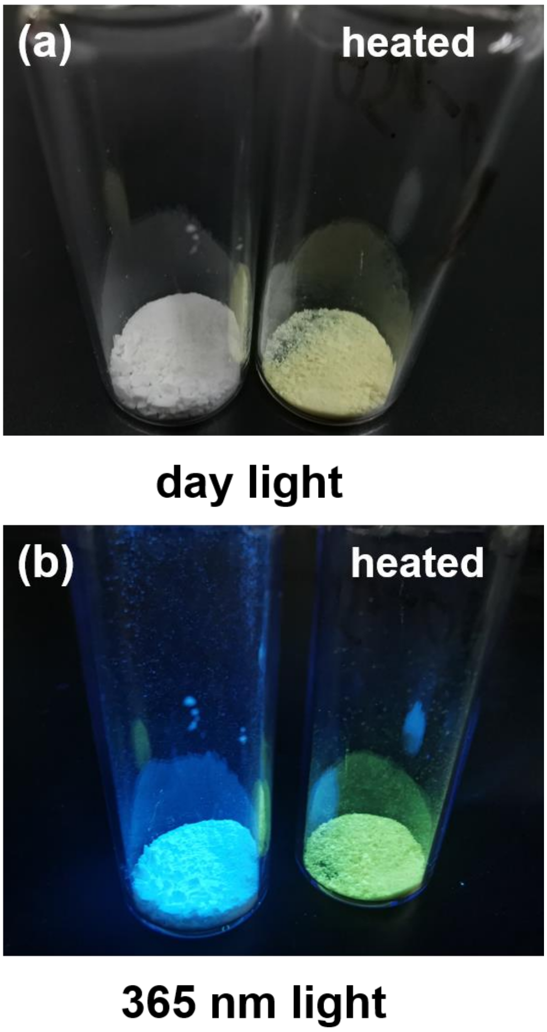

Supplementary Figure 15. The photographs of as-synthesized Zr-TCPE and Zr-TCPE-H under (a) daylight and (b) 365 nm ultraviolet light, respectively.

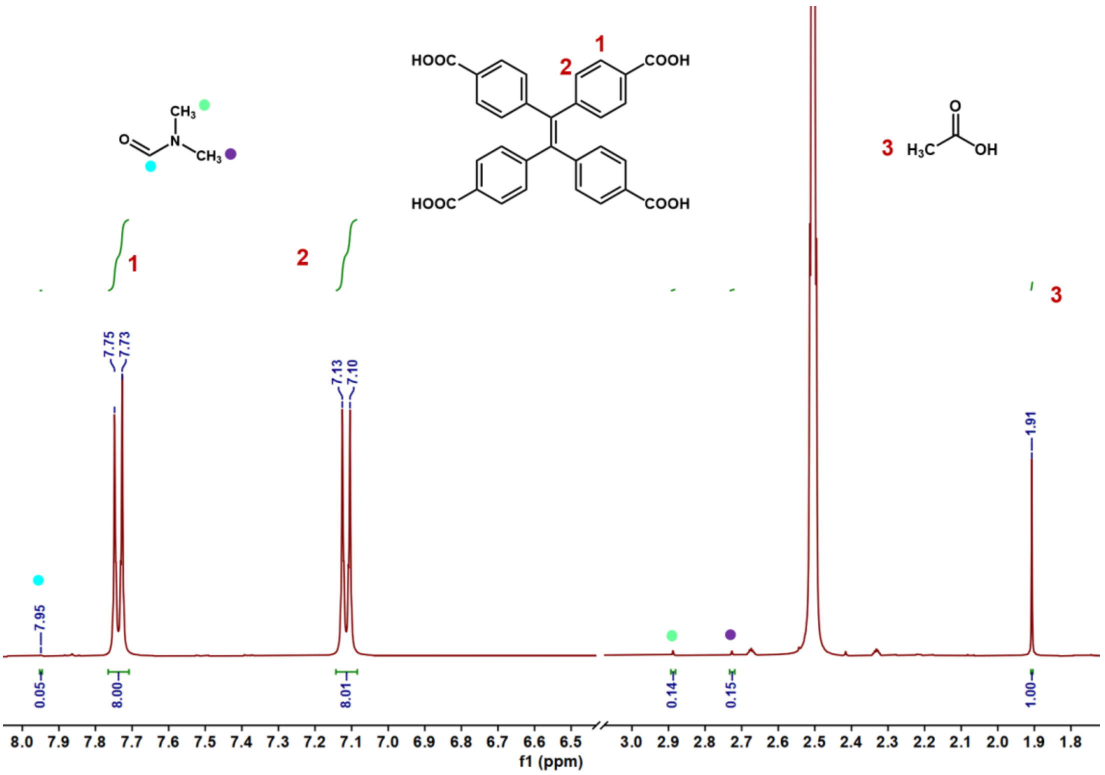

Supplementary Figure 16. The ^{1}H NMR spectroscopy of digested Zr-TCPE-H (TCPE: AA: DMF = 1: 0.3: 0.05). The amount of DMF in Zr-TCPE decreased apparently after heating treatment.

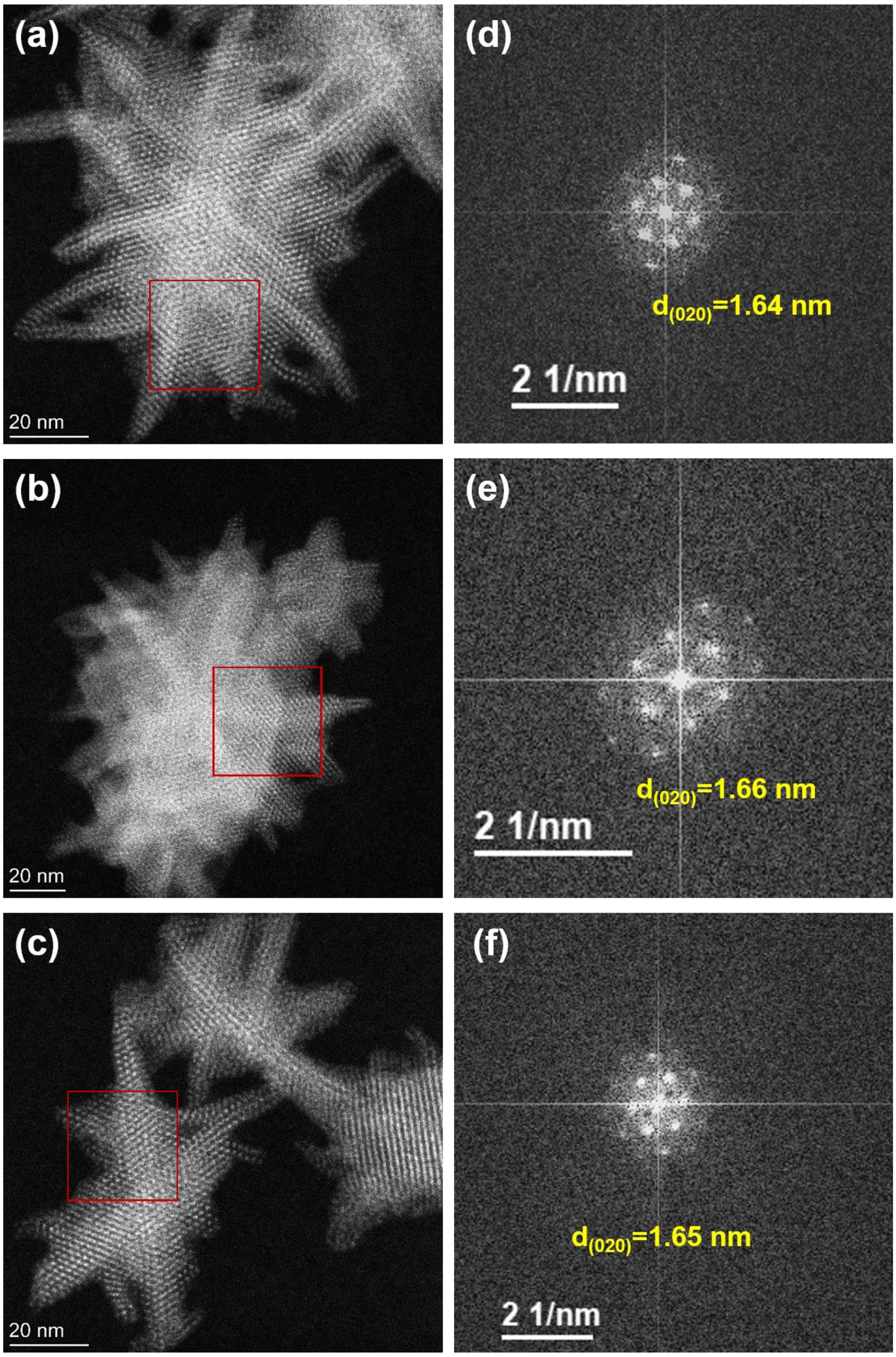

Supplementary Figure 17. (a-c) The HAADF images of Zr-TCPE-H and (d-f) the corresponding FFT images. The white dots in the HAADF images represented the Zr_6 clusters. The d-spacing of the (020) plane was measured at around 1.65 nm.

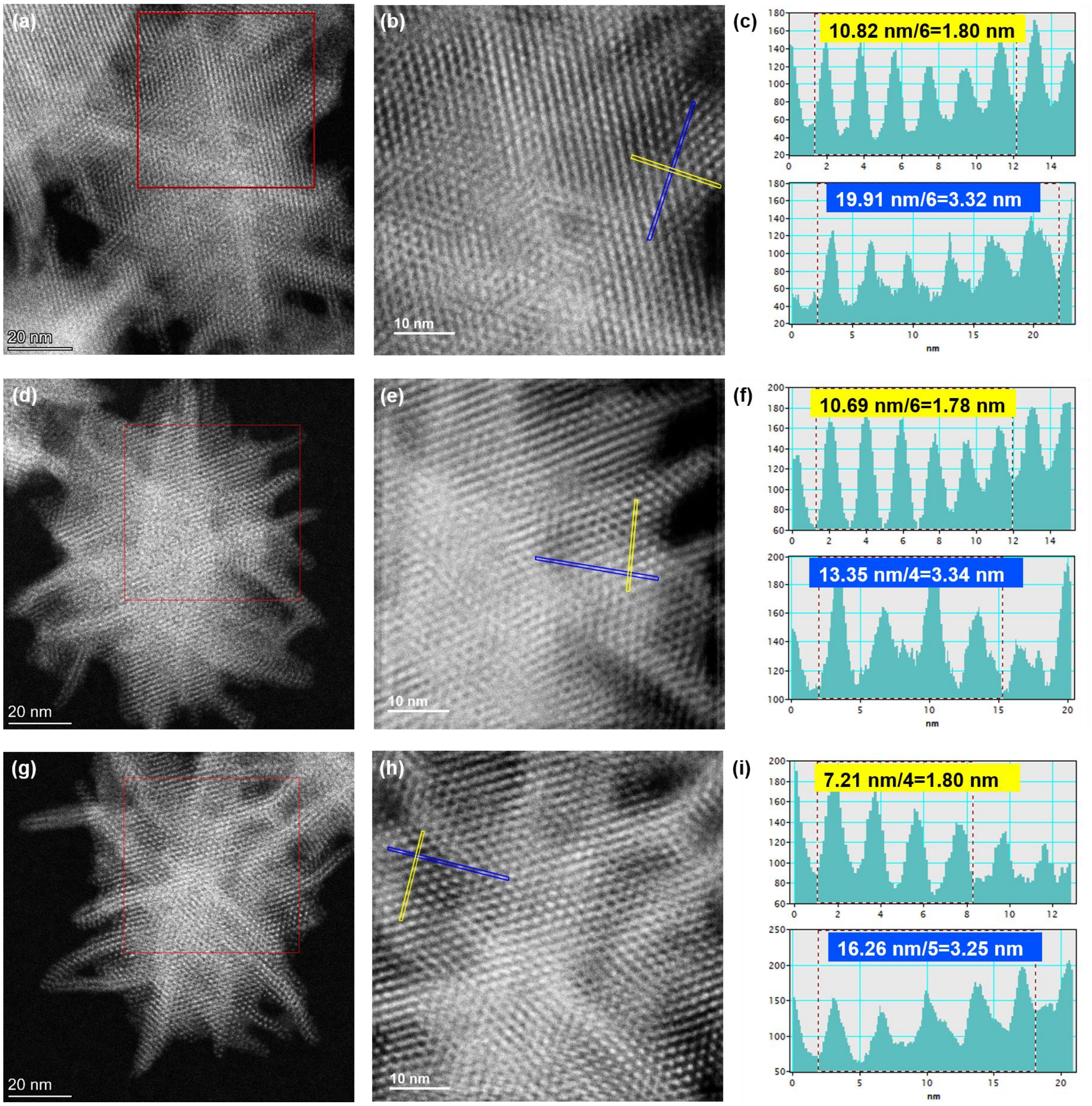

Supplementary Figure 18. (a,d,g) The HAADF images of Zr-TCPE-H. The white dots in the HAADF images represented the Zr_6 clusters. (b,e,h) The denoised HAADF images of the red square region. The denoised image was acquired by an inverse-FFT process after applying a periodic mask to the FFT pattern. (c,f, i) The measured distance of adjacent Zr_6 clusters was along the a-axis (labeled in yellow) and the b-axis (labeled in blue). The distance of adjacent Zr_6 clusters along the a-axis and b-axis was around 1.79 nm and 3.25 nm, respectively.

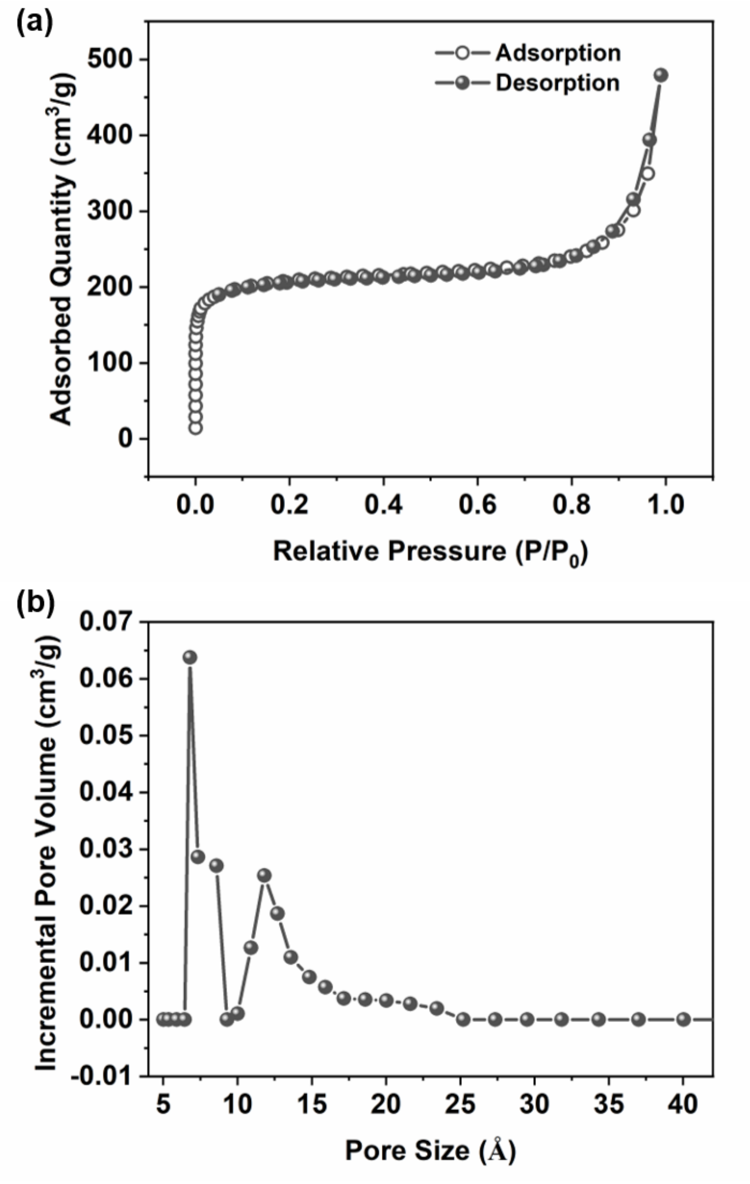

Supplementary Figure 19. (a) The N_2 adsorption-desorption isotherms and (b) pore size distribution of Zr-TCPE-H measured at 77 K.

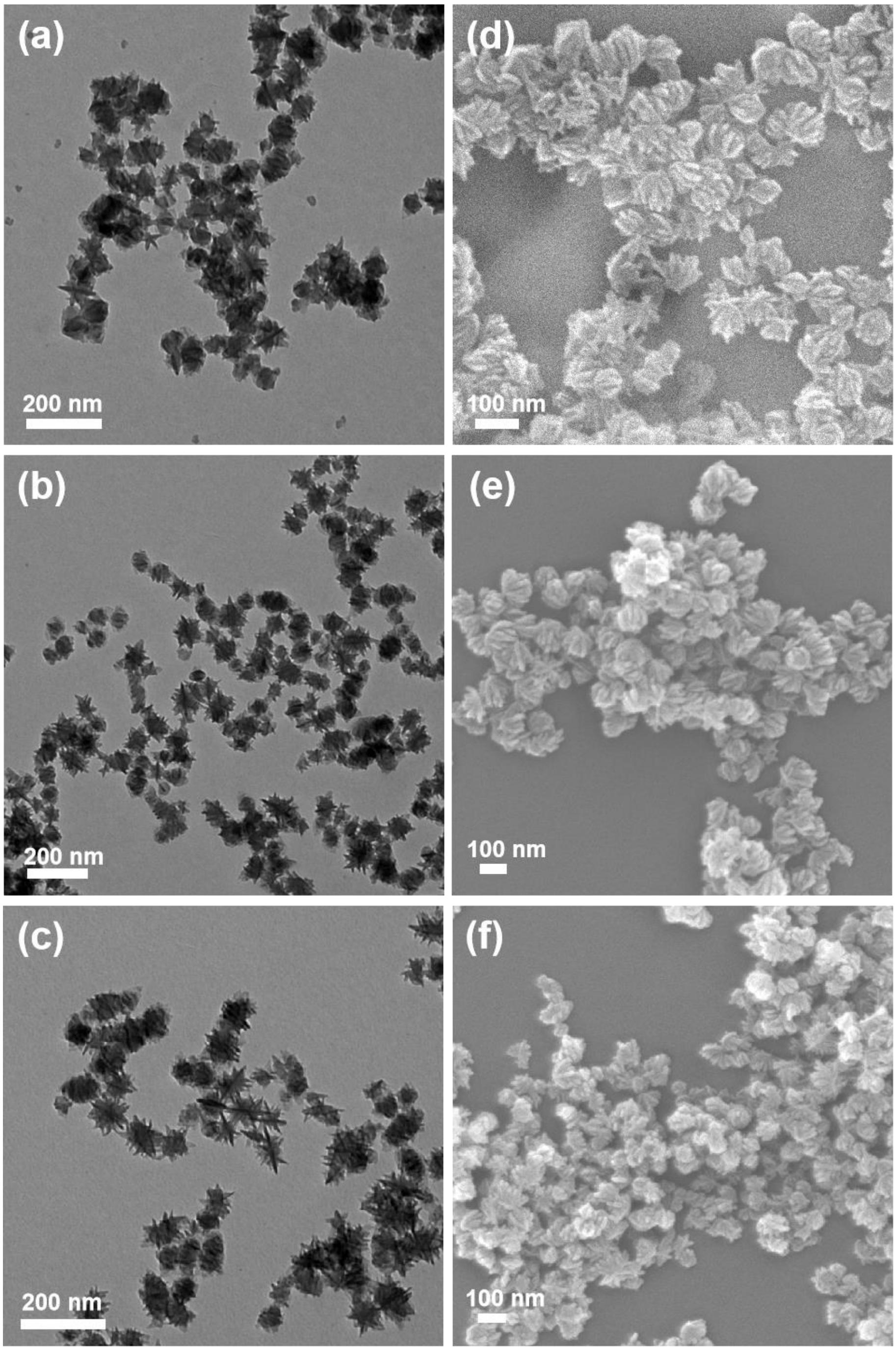

Supplementary Figure 20. The TEM (a-c) and SEM (d-f) images of Zr-TCPE after the heating process. (a,d) Zr-TCPE-200 ^{o}C**,** (b,e) Zr-TCPE-220 ^{o}C, and (c,f) Zr-TCPE-250 ^{o}C (Zr-TCPE-H).

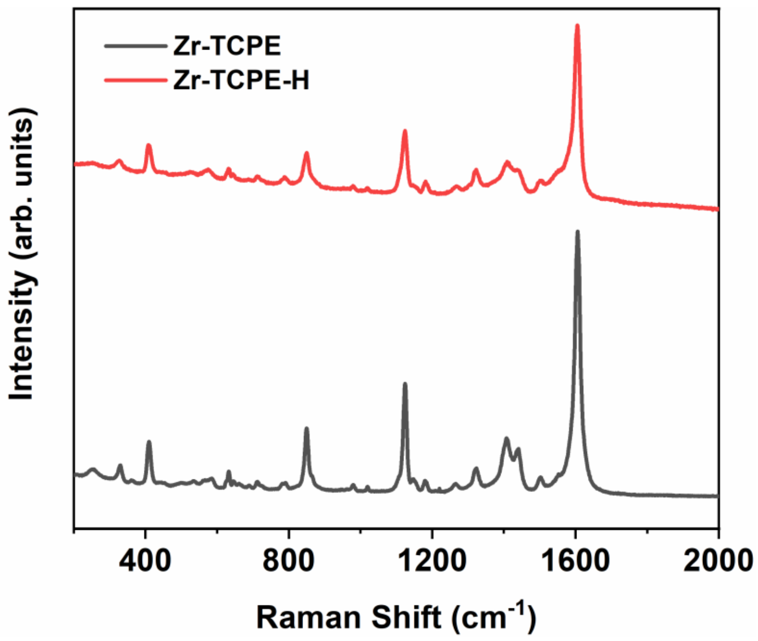

Supplementary Figure 21. The Raman spectra of Zr-TCPE and Zr-TCPE-H.

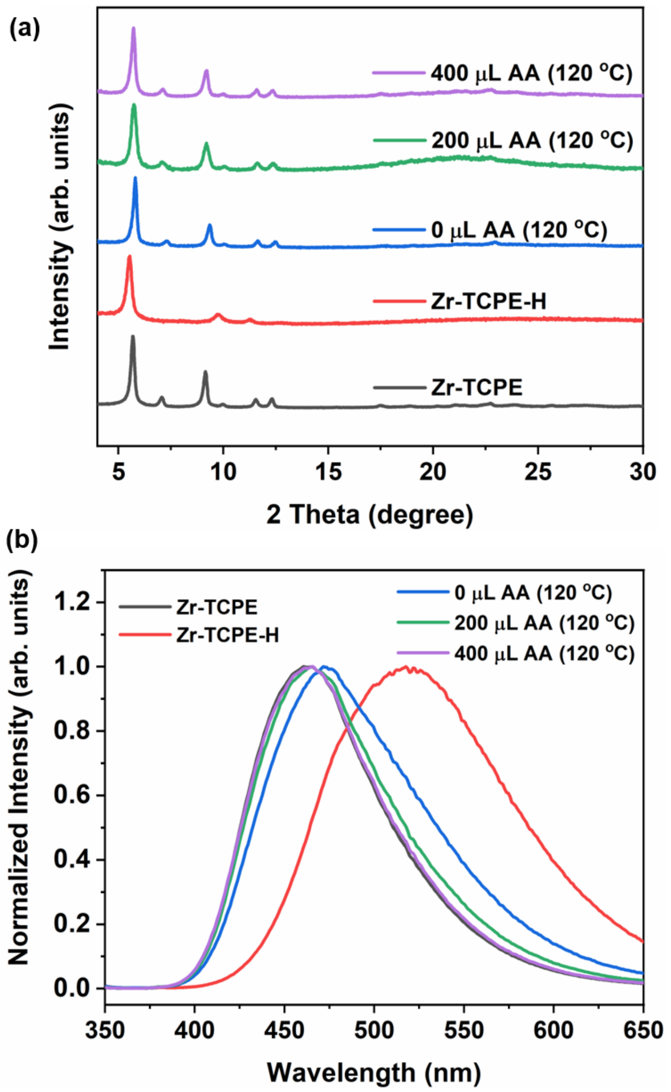

Supplementary Figure 22. (a) The PXRD patterns and (b) the fluorescence spectra of Zr-TCPE-H after treatment in DMF solution with different amounts of acetic acid at 120 °C for 12 h. These MOFs were dispersed in methanol at a concentration of 40 μg/mL (λ_{ex}=340 nm). After treatment at 120 °C without acetic acid, the maximum emission wavelength was 472 nm.

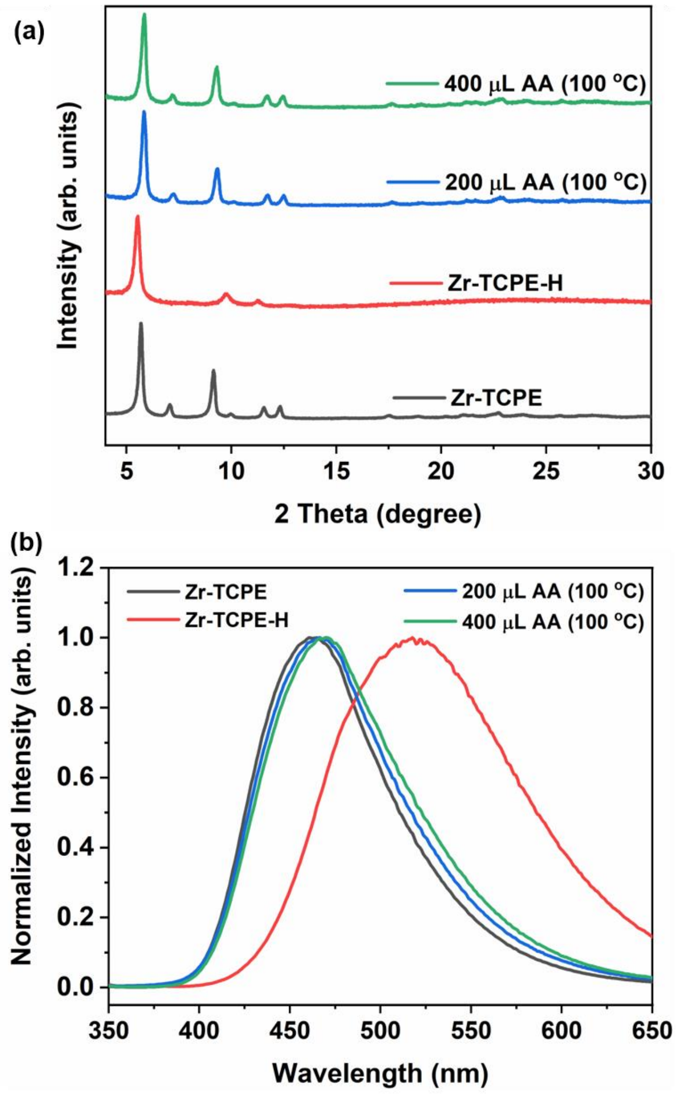

Supplementary Figure 23. (a) The PXRD patterns and (b) the fluorescence spectra of Zr-TCPE-H after treatment in DMF solution with different amounts of acetic acid at 100 ^{o}C for 12 h. These MOFs were dispersed in methanol at a concentration of 40 μg/mL (λ_{ex}=340 nm). The maximum emission wavelengths of Zr-TCPE-H after treatment with 200 μL AA and 400 μL AA were 466 nm and 472 nm, respectively.

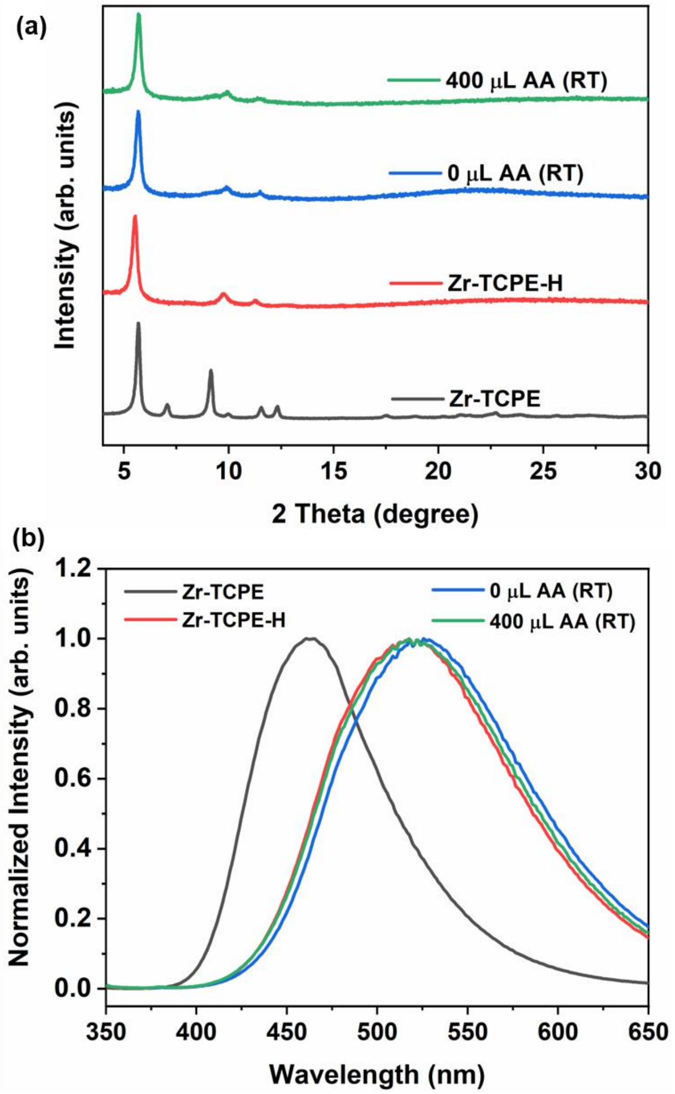

Supplementary Figure 24. (a) The PXRD patterns and (b) the fluorescence spectra of Zr-TCPE-H after treatment in DMF solution with different amounts of acetic acid at room temperature for 12 h. These MOFs were dispersed in methanol at a concentration of 40 μg/mL (λ_{ex}=340 nm). Neither PXRD patterns nor fluorescence spectra of Zr-TCPE-H were restored.

When treated at room temperature, the PXRD patterns and PL properties of Zr-TCPE-H did not restore, despite the addition of DMF and acetic acid. When the temperature was raised to 120 °C, the PXRD pattern and fluorescence emission were restored even without the addition of acetic acid. These experiments verified the importance of temperature in restoring PL properties. Besides, at 120 °C, DMF could hydrolyze into formic acid. Thus, acid coordination is inevitable during structural restoration.

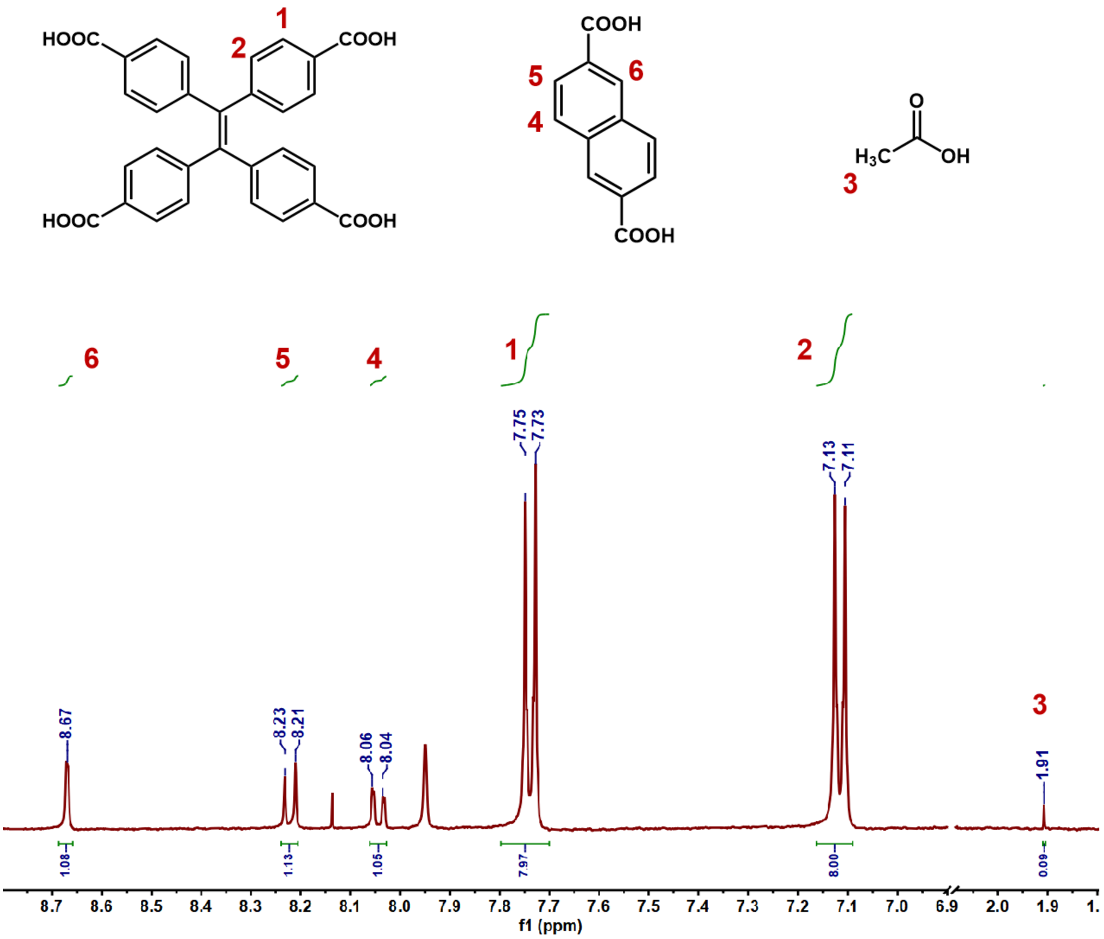

Supplementary Figure 25. The ^{1}H NMR spectroscopy of digested Zr-TCPE-L_1 (TCPE: L_1: AA = 1: 0.5: 0.03).

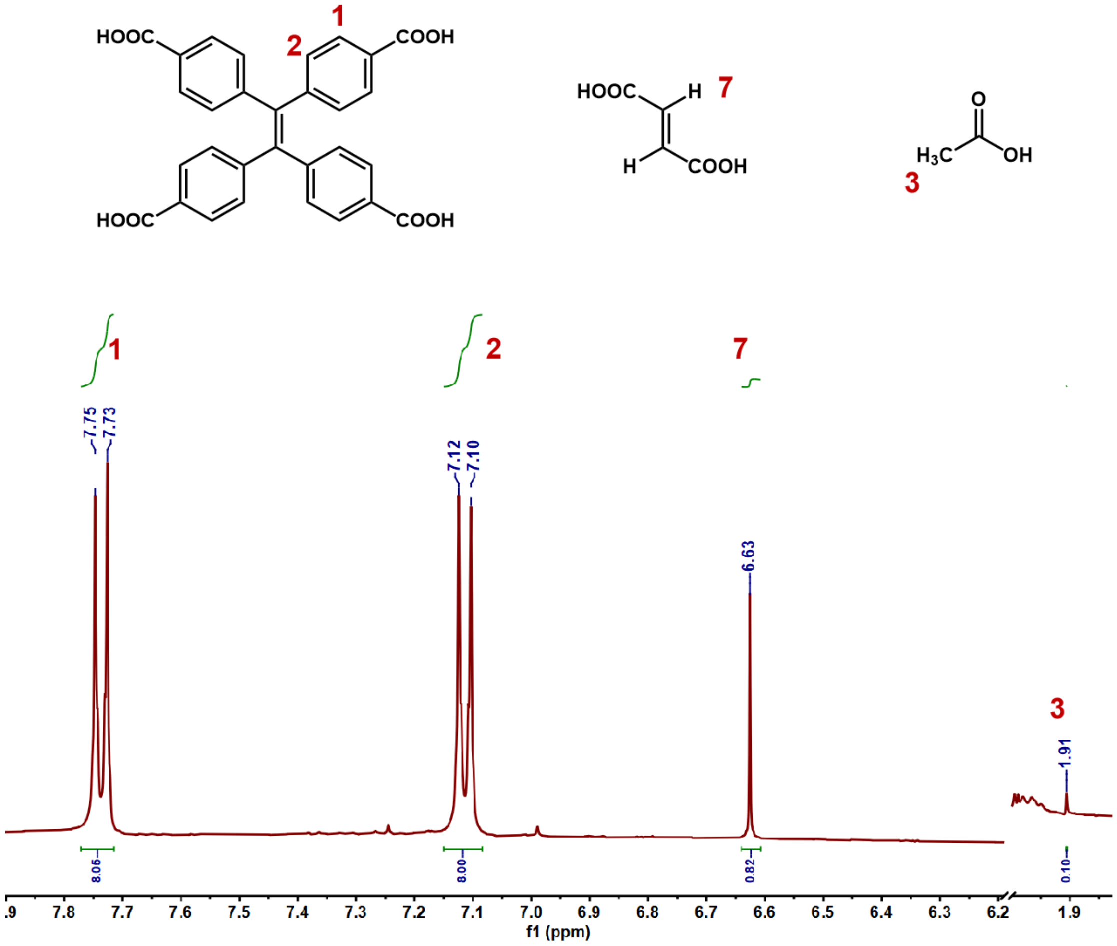

Supplementary Figure 26. The ^{1}H NMR spectroscopy of digested Zr-TCPE-L_2 (TCPE: L_2: AA = 1: 0.4: 0.03).

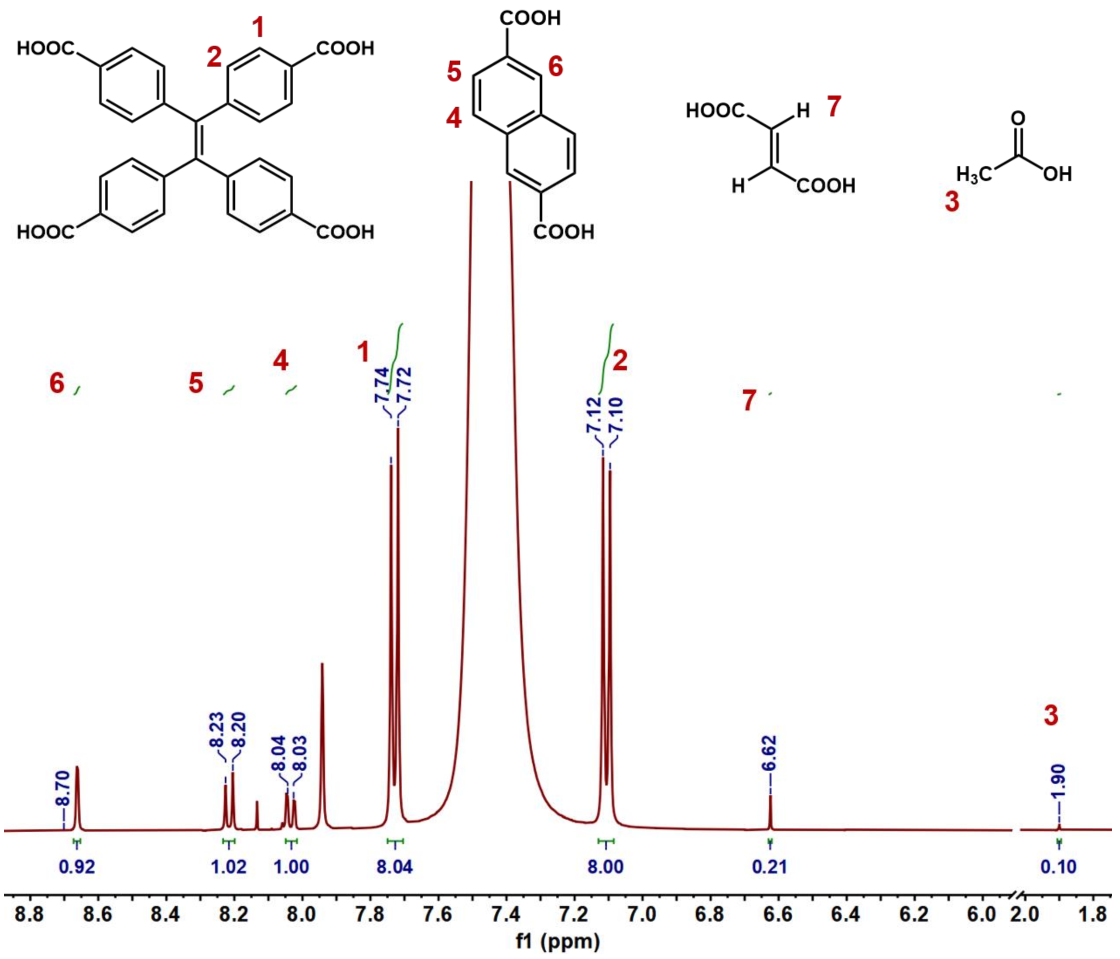

Supplementary Figure 27. The ^{1}H NMR spectroscopy of digested Zr-TCPE-DLI (TCPE: L_1: L_2: AA = 1: 0.5: 0.1: 0.03).

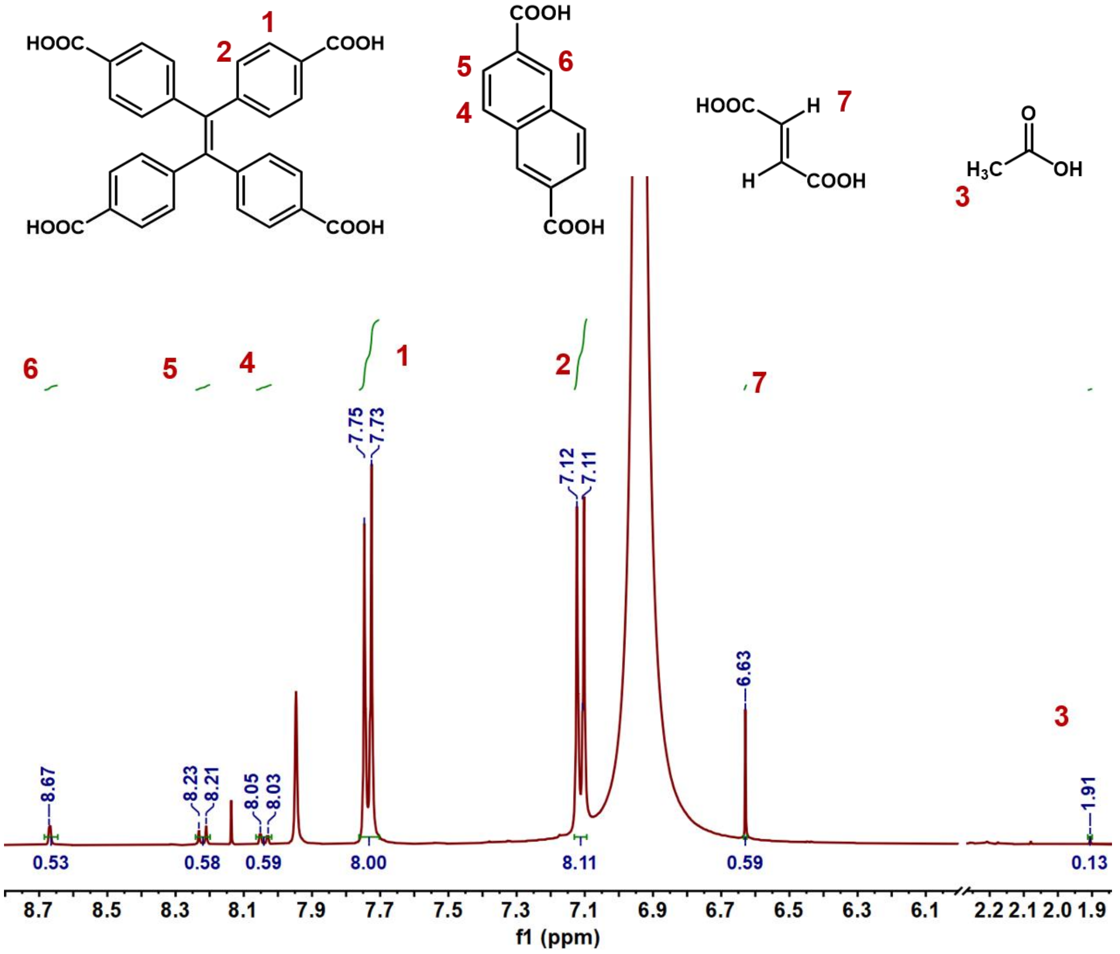

Supplementary Figure 28. The ^{1}H NMR spectroscopy of digested Zr-TCPE-DLI' (TCPE: L_1: L_2: AA = 1: 0.3: 0.3: 0.04).

The ligand ratio of L_1: L_2: TCPE in the synthesized Zr-TCPE-DLI (L_2-L_1) and Zr-TCPE-DLI' (L_1-L_2) was calculated from the ^{1}H NMR results. The ligand ratio of L_1: L_2: TCPE was 0.3: 0.3: 1 in Zr-TCPE-DLI'. The ratio of L_1 was much less than 0.5, which might result from the competitive coordination of L_2 at pocket A. Meanwhile, the ratio of L_2 was slightly less than 0.4, we considered that the L_2 dangled at pocket A was not stable and can be partly removed by the solvent exchange process. The ligand ratio of L_1: L_2: TCPE was 0.5: 0.1: 1 in Zr-TCPE-DLI. Considering that L_1 can only coordinate at pocket A, the results illustrated that the L_2 dangled at pocket A was replaced by L_1, leading to this full coordination of L_1 at pocket A. Thus, L_1 mainly existed in pocket A and L_2 mainly existed in pocket B in Zr-TCPE-DLI, preventing the intramolecular rotation and partitioning pore.

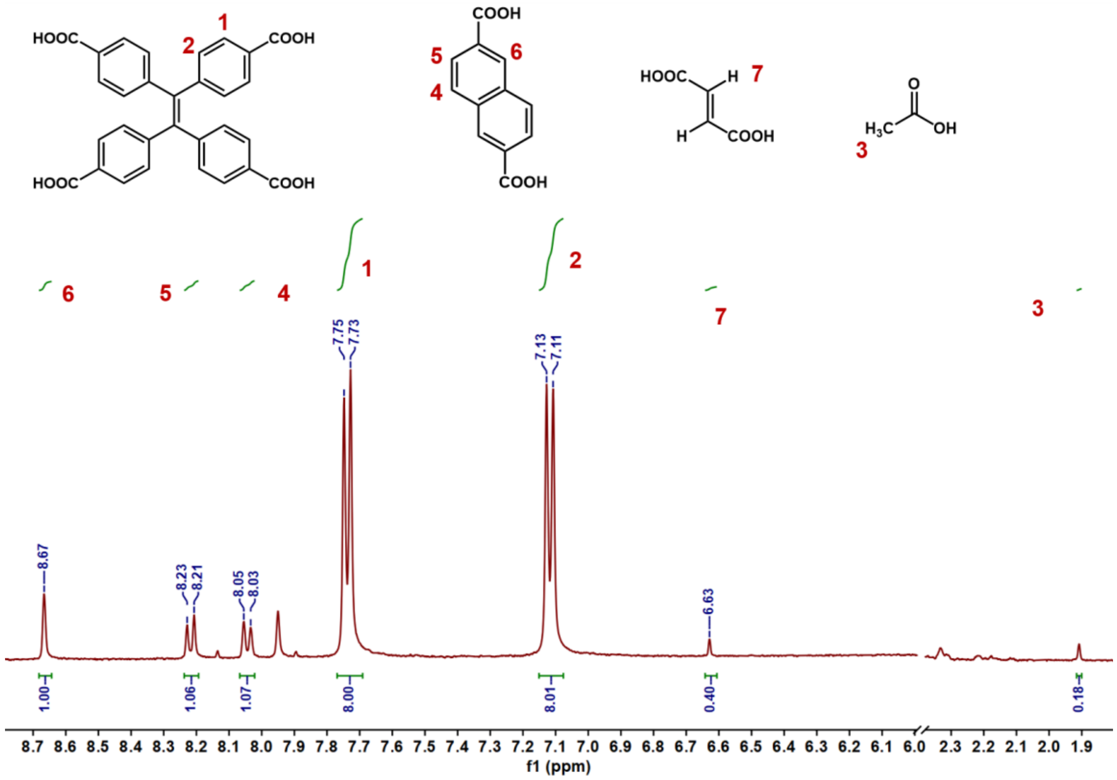

Supplementary Figure 29. The ^{1}H NMR spectroscopy of digested Zr-TCPE-DLI with twice the amount of linkers (TCPE: L_1: L_2: AA = 1: 0.5: 0.2: 0.06).

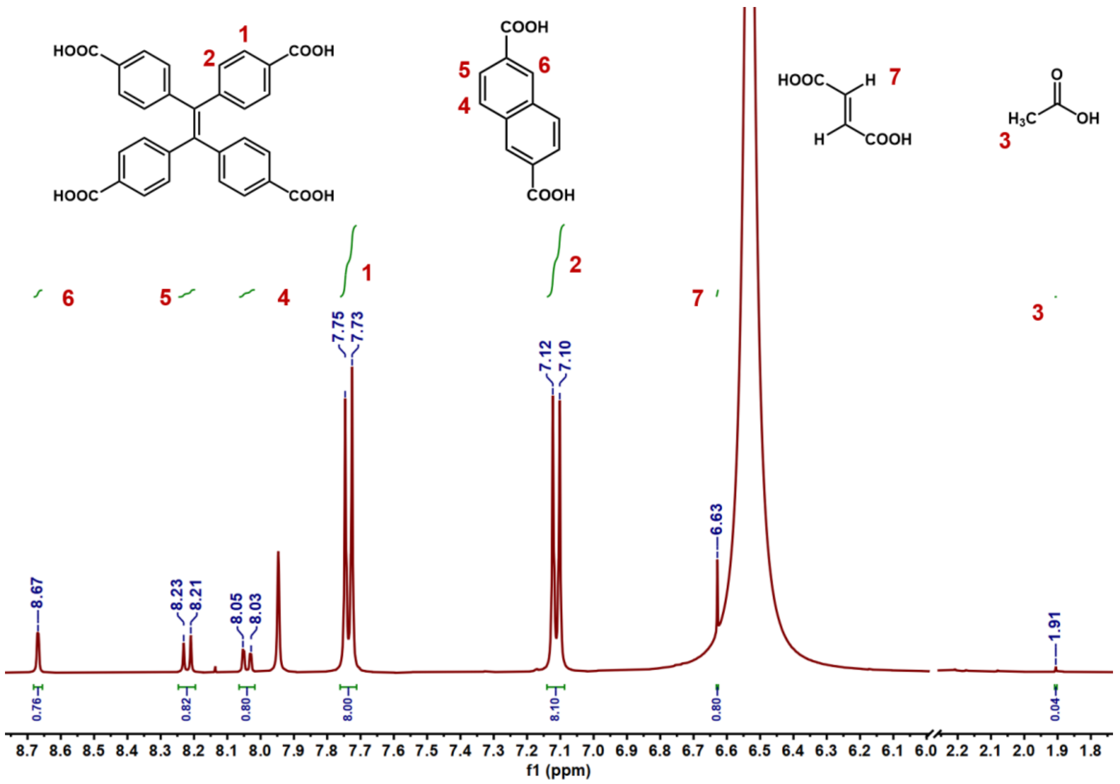

Supplementary Figure 30. The ^{1}H NMR spectroscopy of digested Zr-TCPE-DLI with the addition of L_1 and L_2 simultaneously (TCPE: L_1: L_2: AA = 1: 0.4: 0.4: 0.01).

Further increasing the amount of L_1 and L_2 in the synthesis of Zr-TCPE-DLI, the ratio of L_1: L_2: TCPE was 0.5: 0.2: 1. The pocket B was still not fully occupied. Besides, adding L_1 and L_2 simultaneously in the synthesis process, the ratio of L_1: L_2: TCPE was 0.4: 0.4: 1, indicating neither pocket was fully occupied.

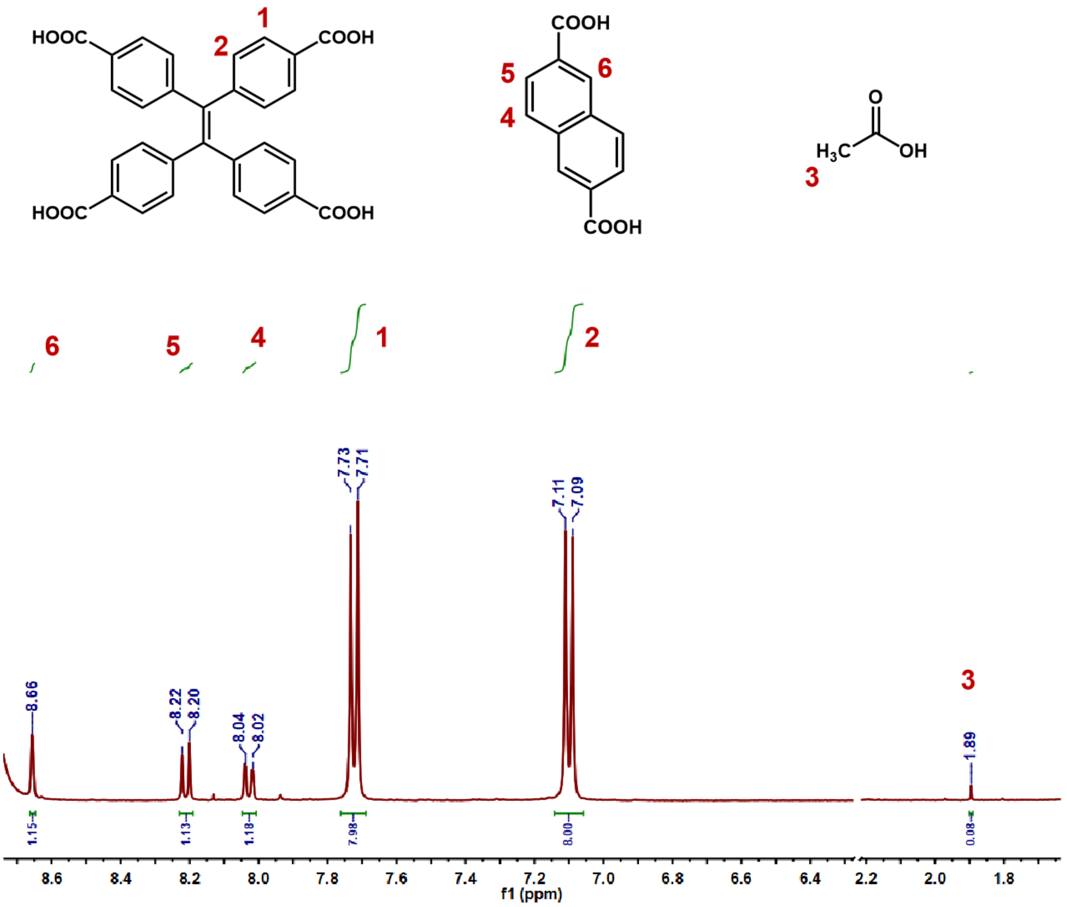

Supplementary Figure 31. The ^{1}H NMR spectroscopy of digested Zr-TCPE-L_1-H (TCPE: L_1: AA = 1: 0.5: 0.03).

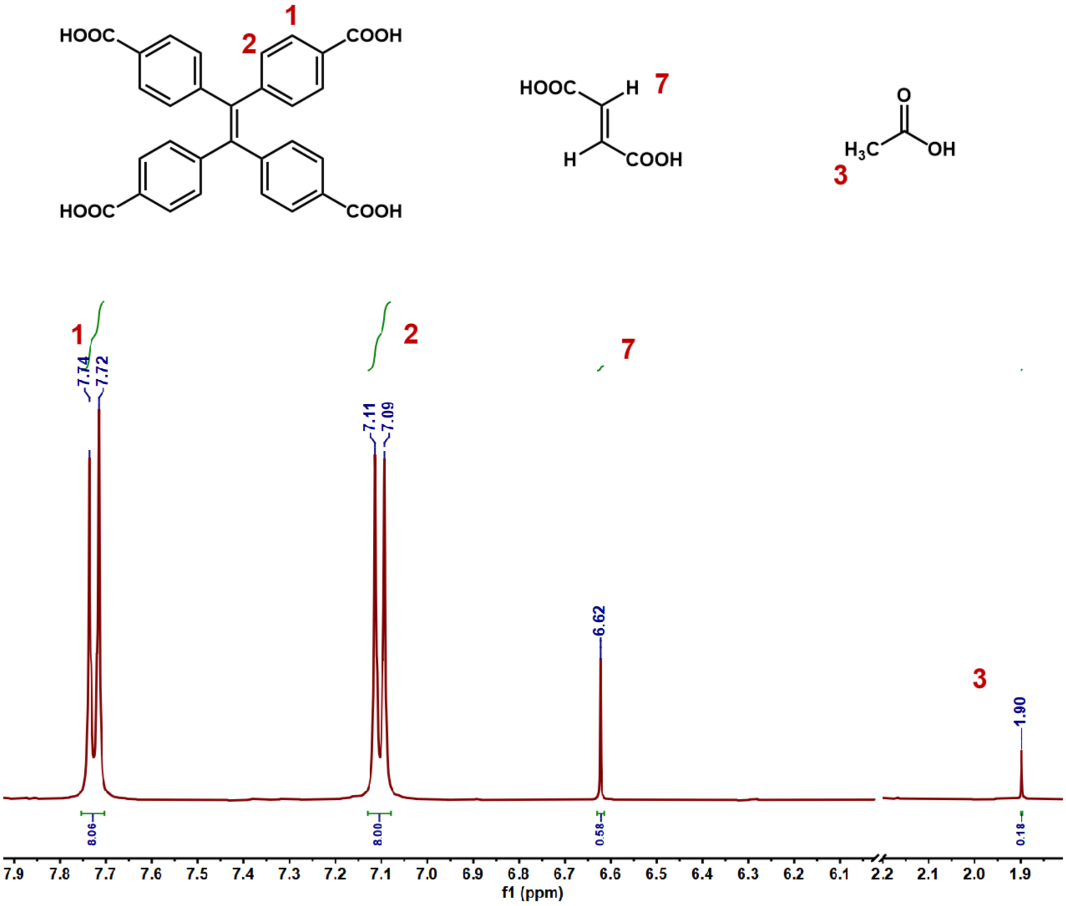

Supplementary Figure 32. The ^{1}H NMR spectroscopy of digested Zr-TCPE-L_2-H (TCPE: L_2: AA = 1: 0.3: 0.06).

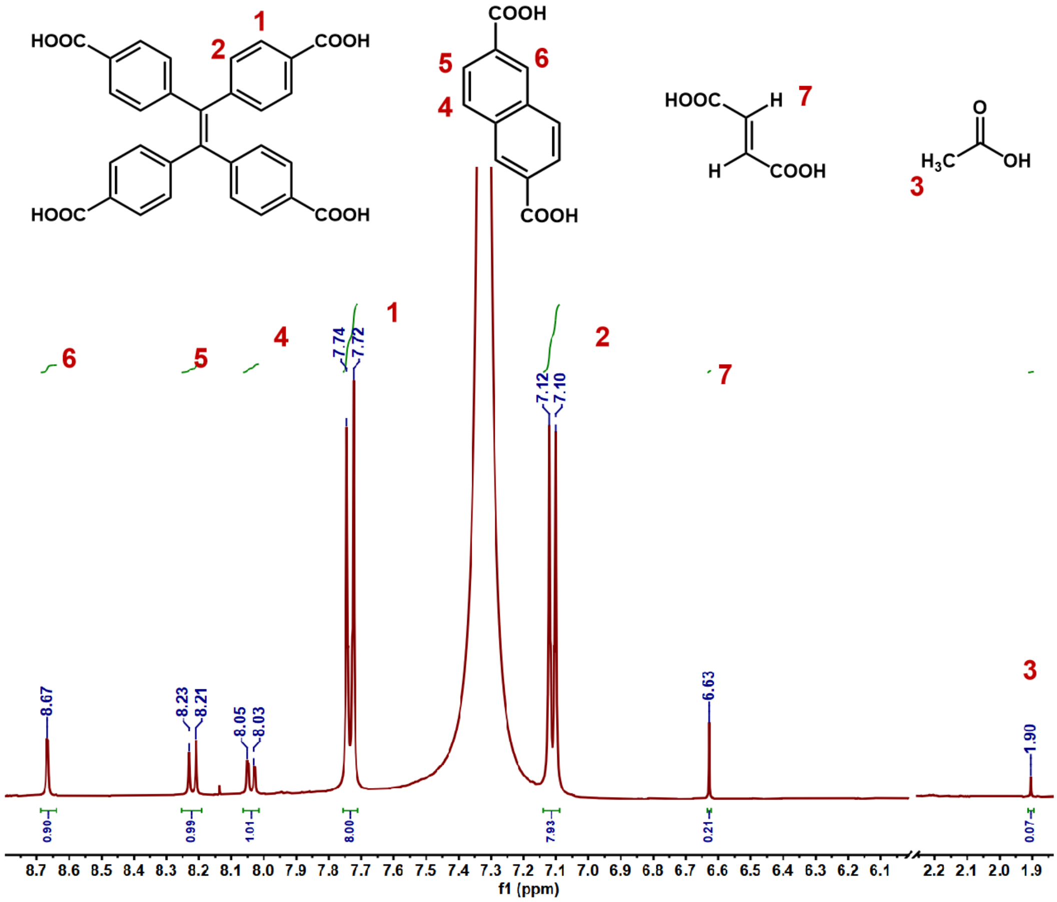

Supplementary Figure 33. The ^{1}H NMR spectroscopy of digested Zr-TCPE-DLI-H (TCPE: L_1: L_2: AA = 1: 0.5: 0.1: 0.02).

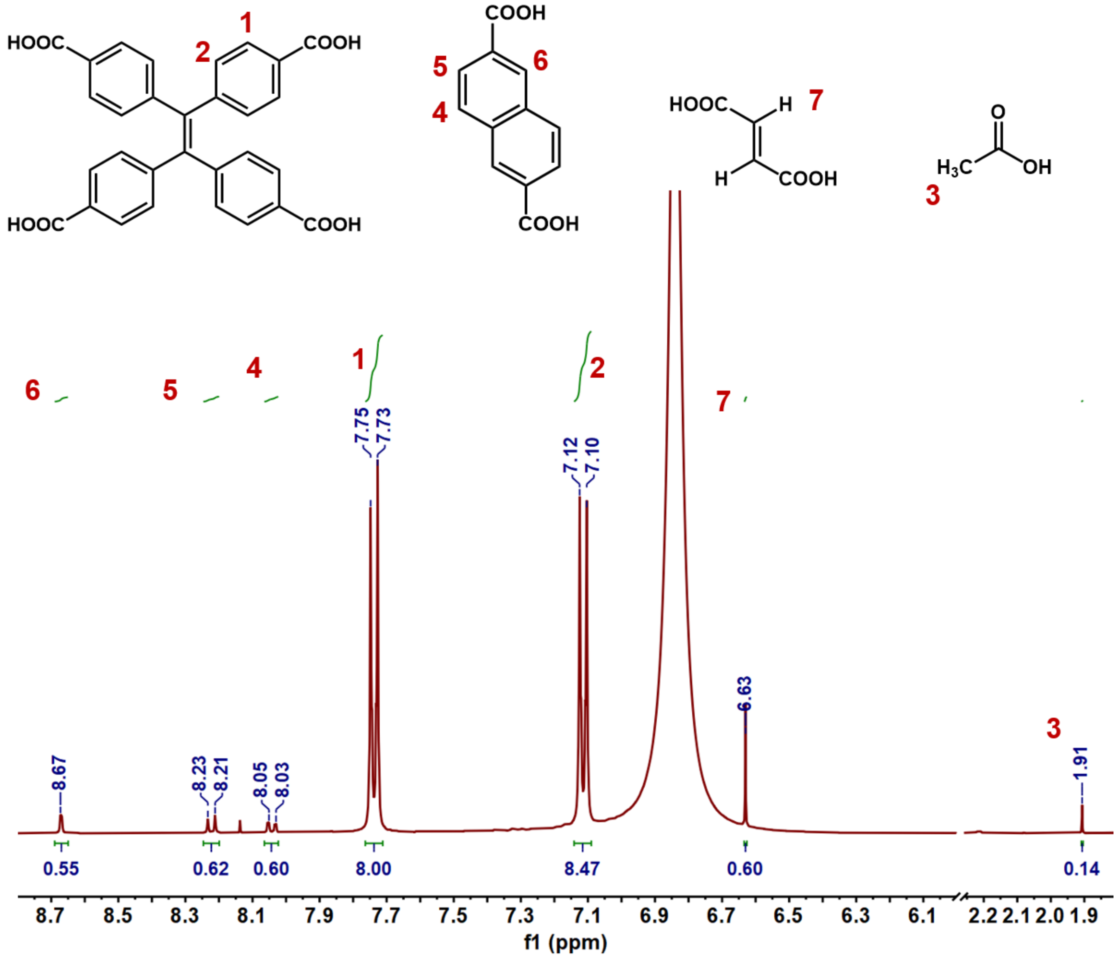

Supplementary Figure 34. The ^{1}H NMR spectroscopy of digested Zr-TCPE-DLI'-H (TCPE: L_1: L_2: AA = 1: 0.3: 0.3: 0.04).

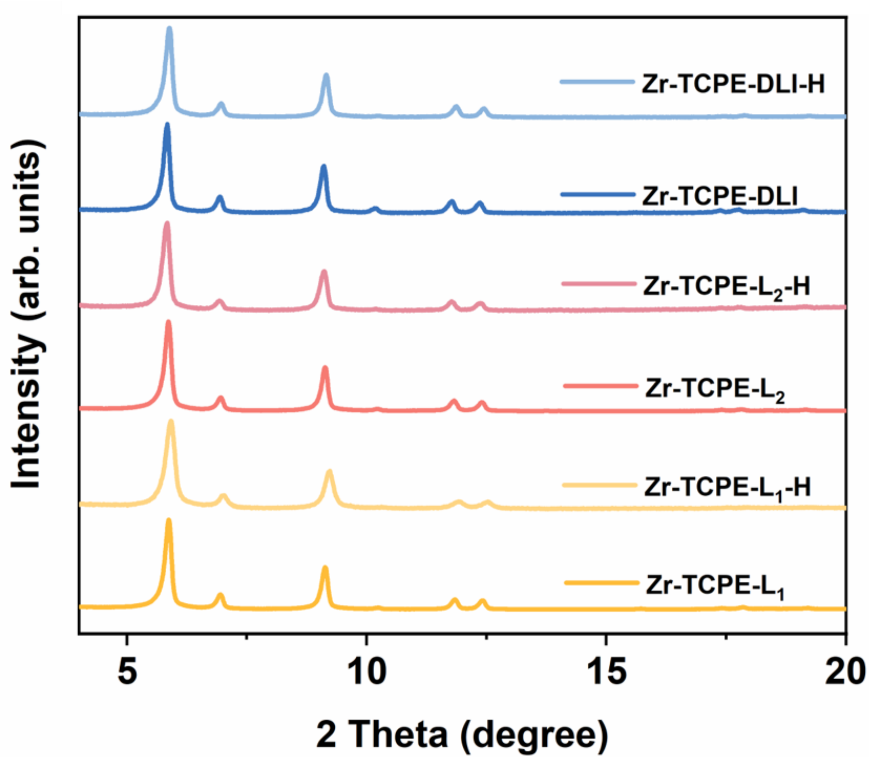

Supplementary Figure 35. The PXRD patterns of Zr-TCPE-L_1, Zr-TCPE-L_1-H, Zr-TCPE-L_2, Zr-TCPE-L_2-H, Zr-TCPE-DLI, and Zr-TCPE-DLI-H.

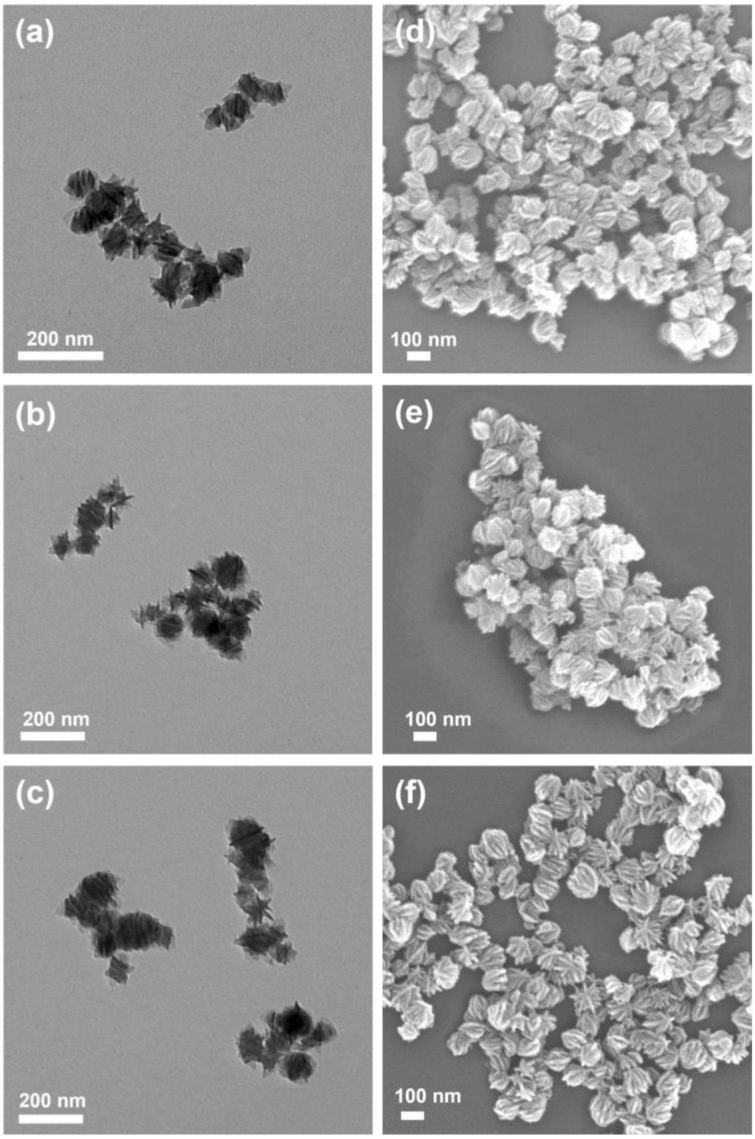

Supplementary Figure 36. (a-c) TEM and (d-f) SEM images of Zr-TCPE with linker insertion. (a,d) Zr-TCPE-L_1, (b,e) Zr-TCPE-L_2, and (c,f) Zr-TCPE-DLI.

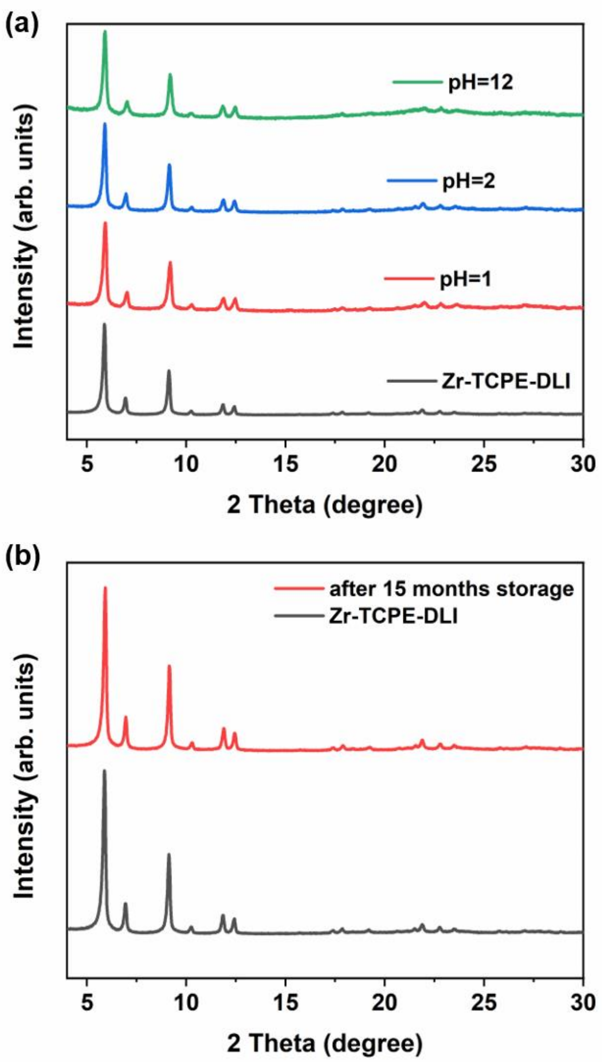

Supplementary Figure 37. The PXRD patterns of (a) Zr-TCPE-DLI immersing in aqueous solutions with different pH values for 24 h and (b) Zr-TCPE-DLI after 15 months of storage. The crystallinity of Zr-TCPE-DLI was well maintained, indicating enhanced stability.

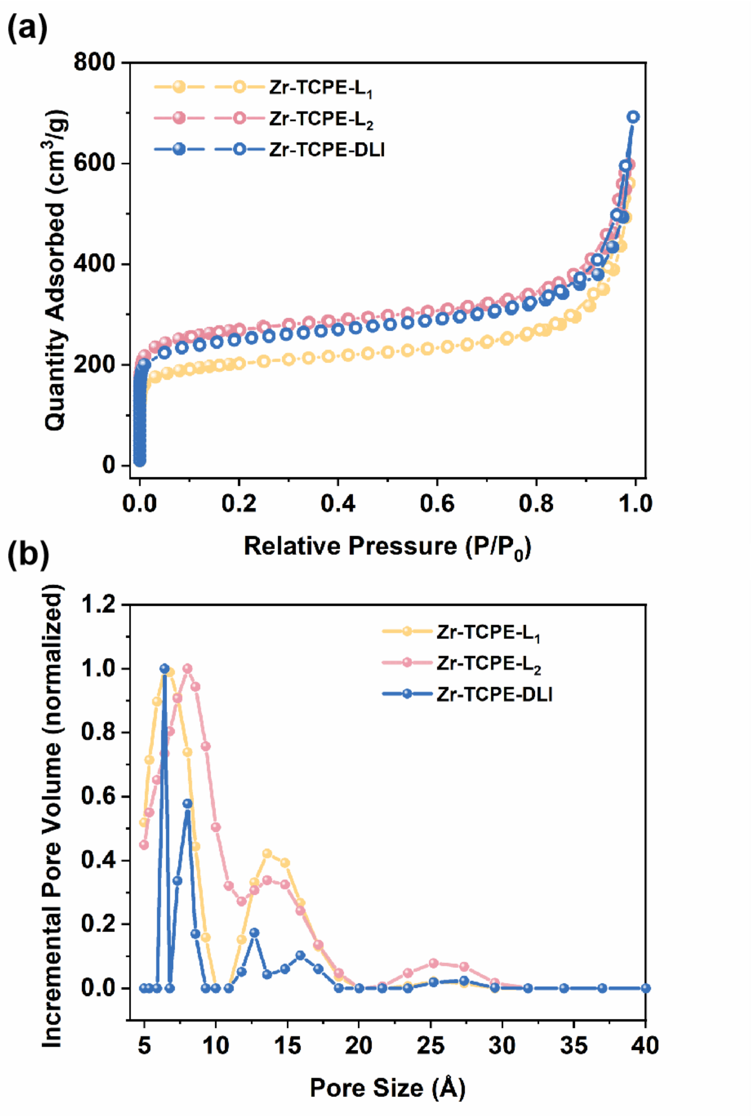

Supplementary Figure 38. (a) The N_2 adsorption-desorption isotherms and (b) pore size distribution of Zr-TCPE-L_1, Zr-TCPE-L_2, and Zr-TCPE-DLI measured at 77K.

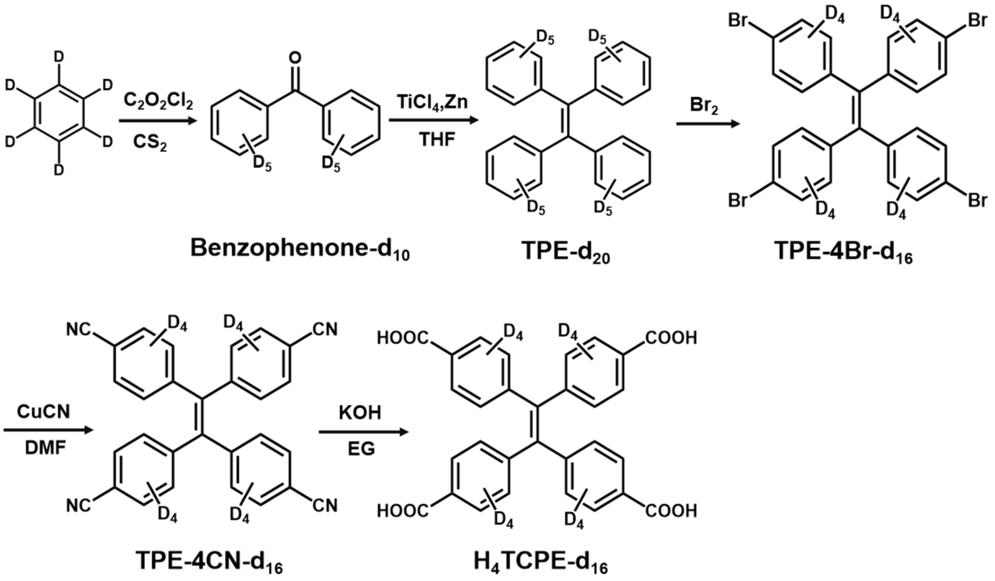

Supplementary Figure 39. Synthesis route of H_4TCPE-d_{16}.

Synthesis of benzophenone-d_{10}.[3] The anhydrous $AlCl_3$ (2.6 g) was slowly added to the mixture of benzene-d_6 (2.505 mL), oxalyl chloride (0.854 mL), and carbon disulfide (5 mL). After no gas is produced, the reaction was heated at 60 ^oC for 2 h. The mixture was concentrated under reduced pressure. The crude product was extracted with ether three times. Then the combined organic phase was dried with anhydrous Na_2SO_4 and concentrated under reduced pressure. A quantity of 2.695 g (52.25 %) of benzophenone-d_{10} was isolated as white powder. ^{13}C NMR ($CDCl_3$): δ = 127.79 (t), 129.67(t), 131.95 (t), 137.43 (s), 196.73 (s) ppm. HRMS: m/z calculated for $C_{13}D_{10}O$: 192.14, found: 192.14.

Synthesis of TPE-d_{20}.[3] Under N_2 atmosphere, zinc powder (3.82 g) was dispersed in anhydrous THF (50 mL) under stirring in the ice bath. Then $TiCl_4$ (3.2 mL) was slowly added to the mixture. The resulting mixture was refluxed for 30 min after stirring for another 10 min. The solution of benzophenone-d_{10} (2.6 g) in THF (10.6 mL) was slowly added to the mixture. Then, the reaction was refluxed for 12 h under N_2 atmosphere. After cooling to room temperature, the crude product was extracted with ethyl acetate three times. Then the combined organic phase was dried with anhydrous Na_2SO_4 and concentrated under reduced pressure. A quantity of 2.053 g (84.45 %) of TPE-d_{20} was isolated as pale yellow powder. ^{13}C NMR ($CDCl_3$): δ = 125.90 (t), 127.13 (t), 130.90 (t), 140.82 (s), 143.56 (s) ppm. HRMS: m/z calculated for $C_{26}D_{20}$: 352.28, found: 352.28. HRMS: m/z calculated for $C_{26}D_{120}H$ $[M+H]^+$: 353.29, found: 353.28.

Synthesis of TPE-4Br-d_{16}.[4] Under N_2 atmosphere, TPE-d_{20} (2.03 g) was dissolved in acetic acid (30 mL) in a two-necked round bottom flask under the ice bath. The bromine (4.8 mL) was slowly injected into the solution with an injection syringe. Then dichloromethane (20 mL) was added to the mixture. After stirring for 15 min, the ice-water bath was removed. The mixture was heated at 50 °C for 15 min. Ice water (200 mL) was poured into the mixture. The crude product was filtered and washed repeatedly with water and ethanol until the yellowish product was obtained. A quantity of 1.042 g (28.81 %) of TPE-4Br-d_{16} was isolated as pale yellow powder. ^{13}C NMR ($CDCl_3$): δ = 121.07 (s), 130.90(t), 132.31 (t), 139.48 (s), 141.32(s) ppm. HRMS: m/z calculated for $C_{26}D_{16}Br_4$: 662.89, found: 663.46.

Synthesis of TPE-4CN-d_{16}.[4] Under N_2 atmosphere, TPE-4Br-d_{20} (1.04 g), CuCN (0.76 g), and dry DMF (20 mL) were heated at reflux for 60 h in a two-necked round bottom flask. Then water (50 mL) was poured into the mixture. After adding ethylenediamine (7.5 mL), the resulting mixture was stirred at 100°C for 1 h. After cooling to room temperature, the crude product was filtered and extracted with dichloromethane three times. The combined organic phase was dried with anhydrous Na_2SO_4 and concentrated under reduced pressure. A quantity of 0.546 g (77.87 %) of TPE-4CN-d_{16} was isolated as pale yellow powder. ^{13}C NMR ($CDCl_3$): δ = 111.82 (s), 118.06 (s), 131.45 (m), 141.38 (s), 145.36 (s) ppm. HRMS: m/z calculated for $C_{30}D_{16}N_4H$ $[M+H]^+$: 449.24, found: 449.29. HRMS: m/z calculated for $C_{30}D_{16}N_4H_2$ $[M+H]^{2+}$: 450.24, found: 450.23.

Synthesis of H_4TCPE-d_{16}.[5] TPE-4CN-d_{16} (0.52 g) and KOH (0.71 g) were refluxed for three days in ethylene glycol (40 mL). After cooling to room temperature, distilled water (20 mL) was added to the mixture. Then the mixture was washed with dichloromethane three times to remove the unreacted TPE-4CN-d_{16}. The mixture was then acidified using 1 M HCl. The precipitate was washed with water and dichloromethane several times and dried under vacuum. A quantity of 0.352 g (54.80 %) of H_4TPE-d_{16} was isolated as pale yellow powder. ^{13}C NMR ($CDCl_3$): δ = 129.19 (m), 129.68 (s), 130.89 (m), 141.49 (s), 146.73 (s), 167.35 (s) ppm.

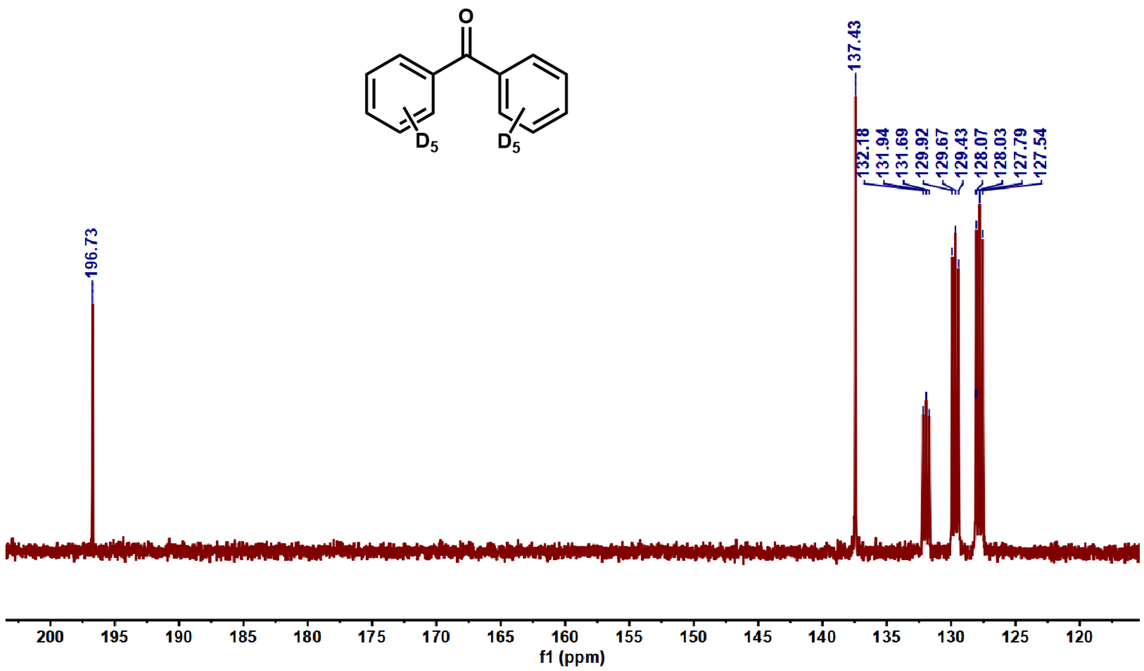

Supplementary Figure 40. The ^{13}C NMR spectroscopy of benzophenone-d_{10} in $CDCl_3$.

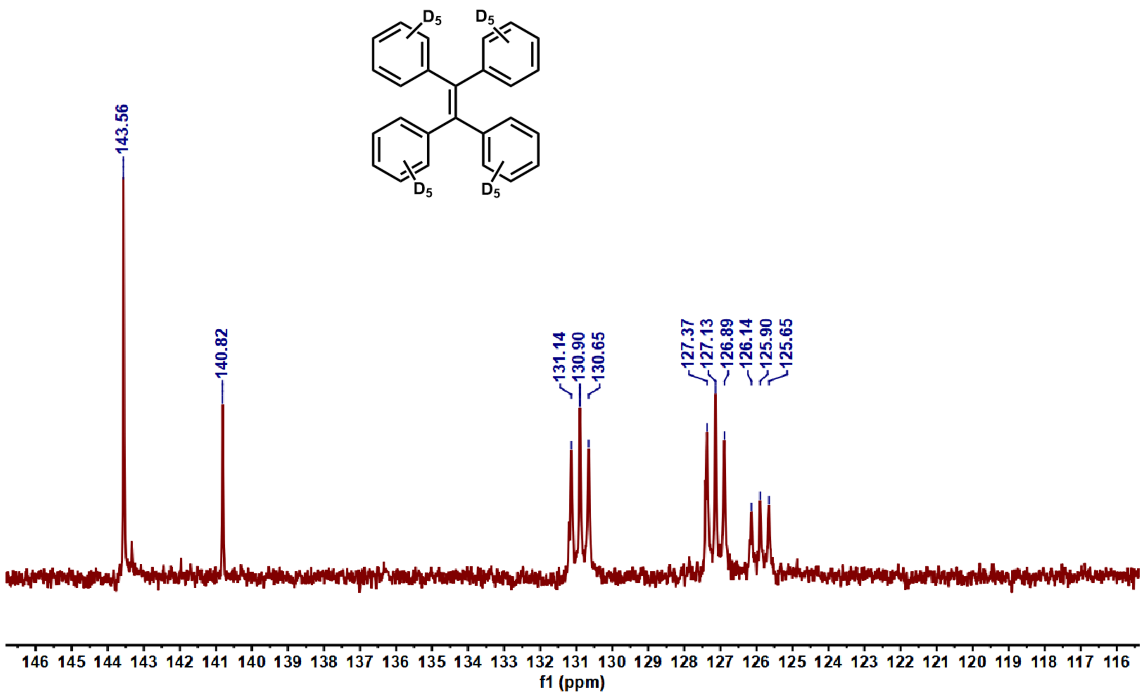

Supplementary Figure 41. The ^{13}C NMR spectroscopy of TPE-d_{20} in $CDCl_3$.

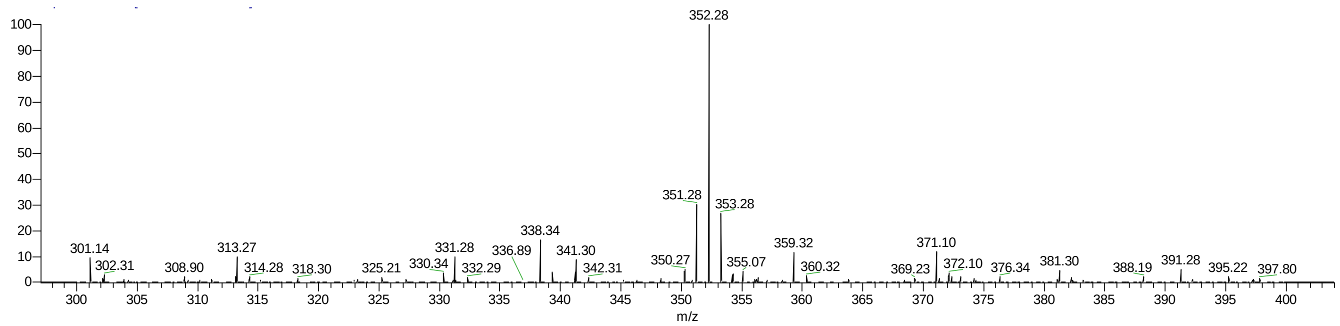

Supplementary Figure 42. The HRMS spectroscopy of TPE-d_{20} in MeOH.

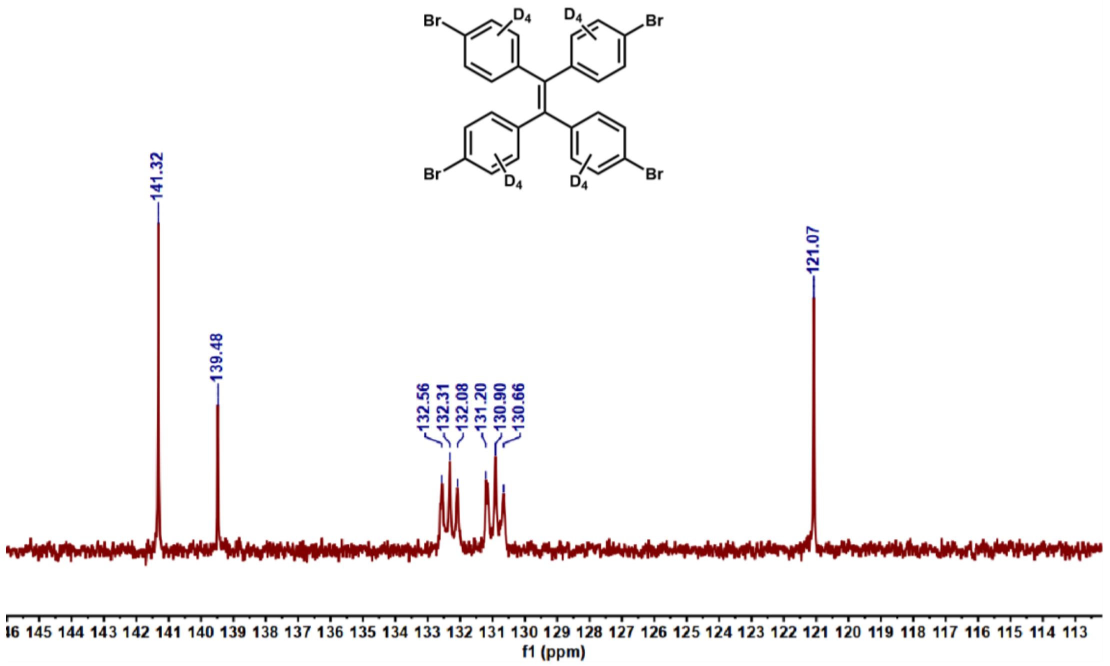

Supplementary Figure 43. The ^{13}C NMR spectroscopy of TPE-4Br-d_{16} in $CDCl_3$.

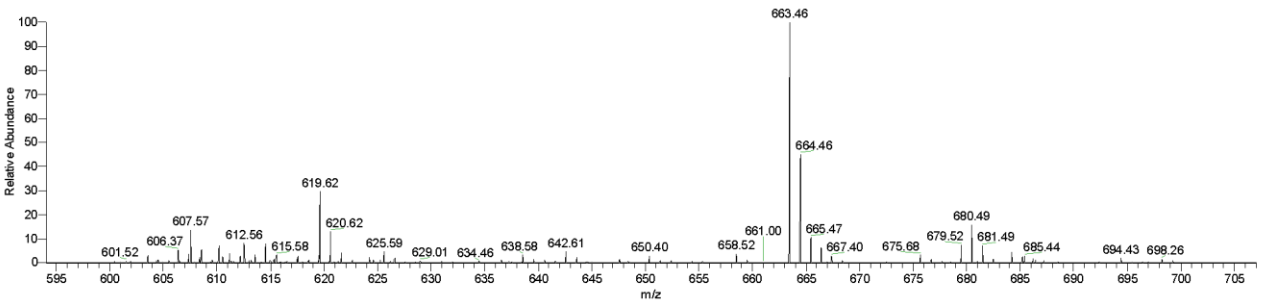

Supplementary Figure 44. The HRMS spectroscopy of TPE-4Br-d_{16} in MeOH.

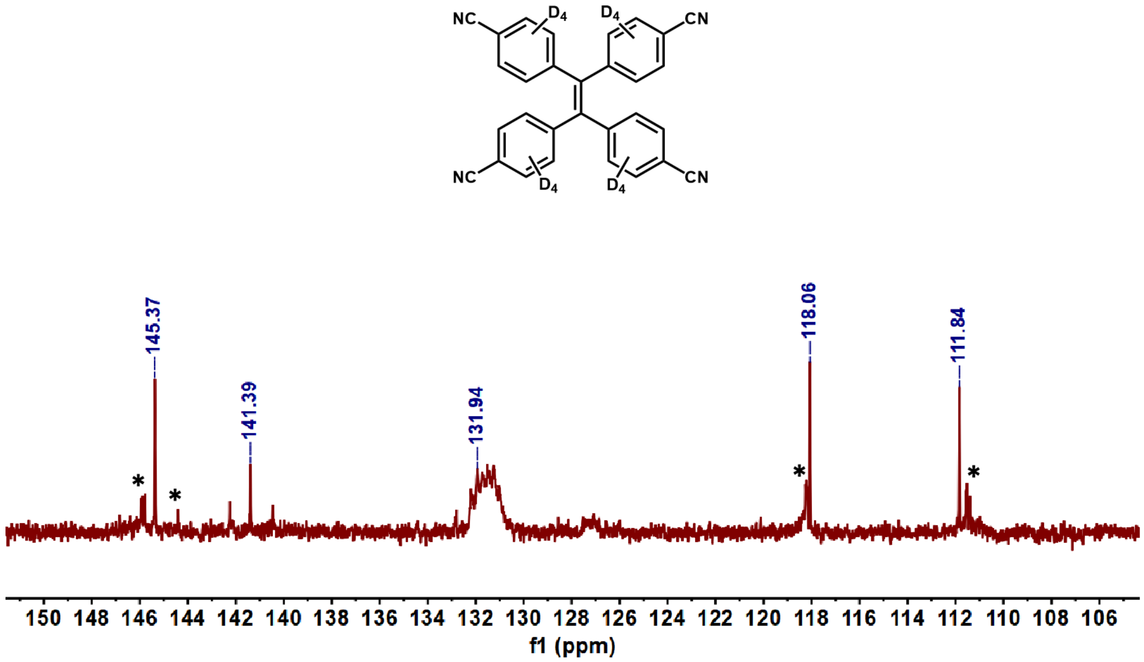

Supplementary Figure 45. The ^{13}C NMR spectroscopy of TPE-4CN-d_{16} in $CDCl_3$. The "*" peaks represent the impurities in the product.

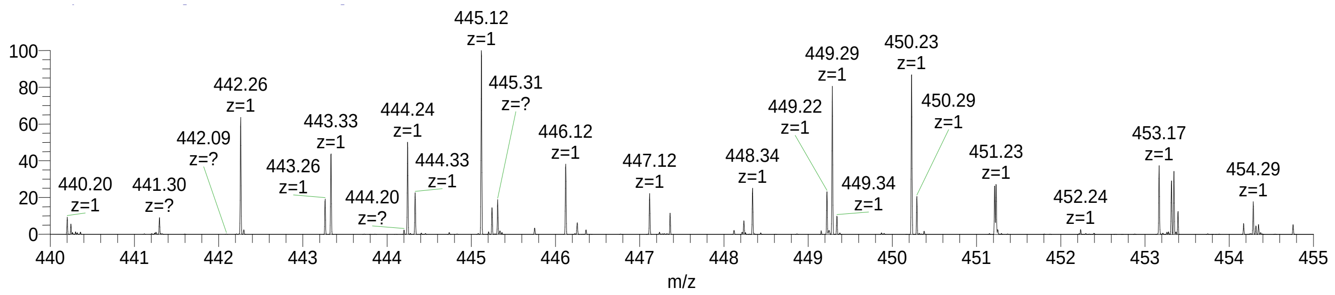

Supplementary Figure 46. The HRMS spectroscopy of TPE-4CN-d_{16} in MeOH.

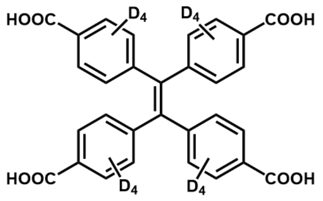

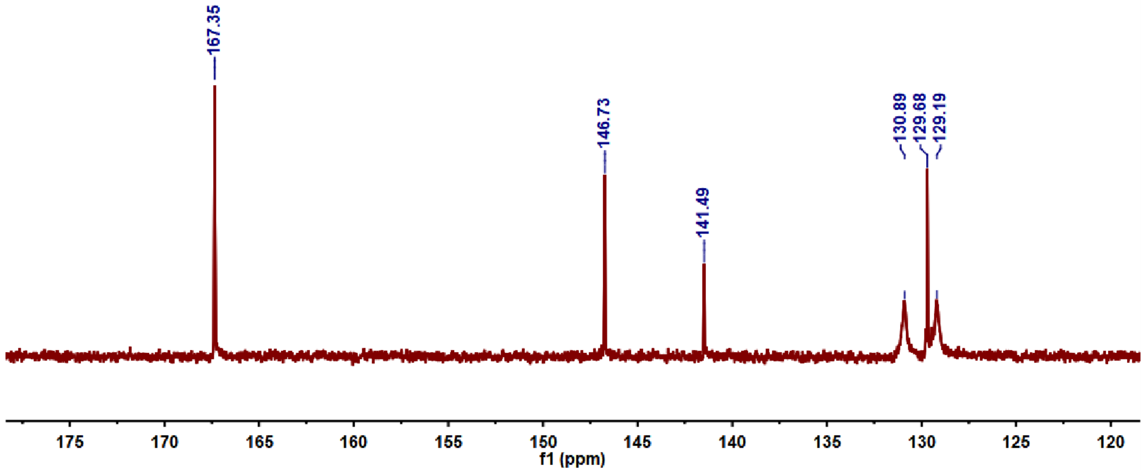

Supplementary Figure 47. The ^{13}C NMR spectroscopy of H_4TPE-d_{16} in $CDCl_3$.

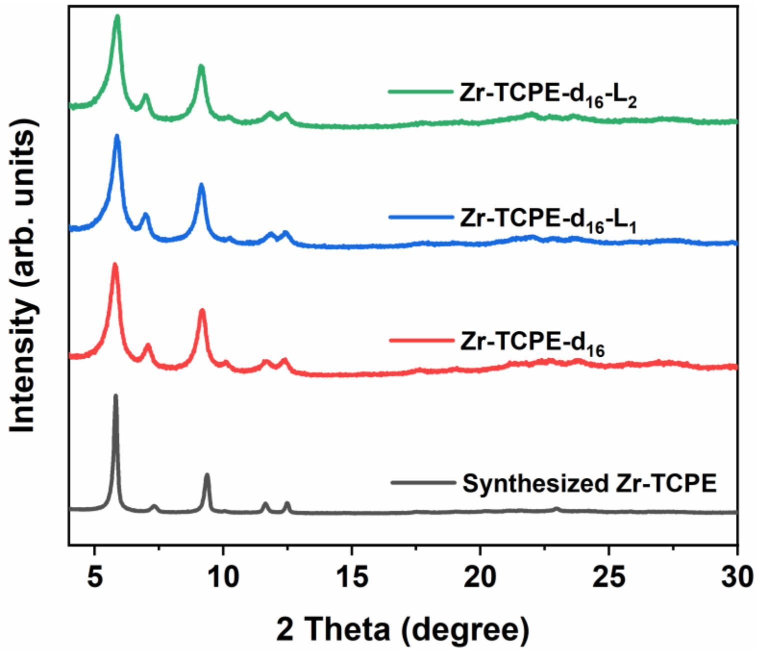

Supplementary Figure 48. The PXRD patterns of Zr-TCPE, Zr-TCPE -L_1, and Zr-TCPE- L_2 constructed with deuterated H_4TCPE.

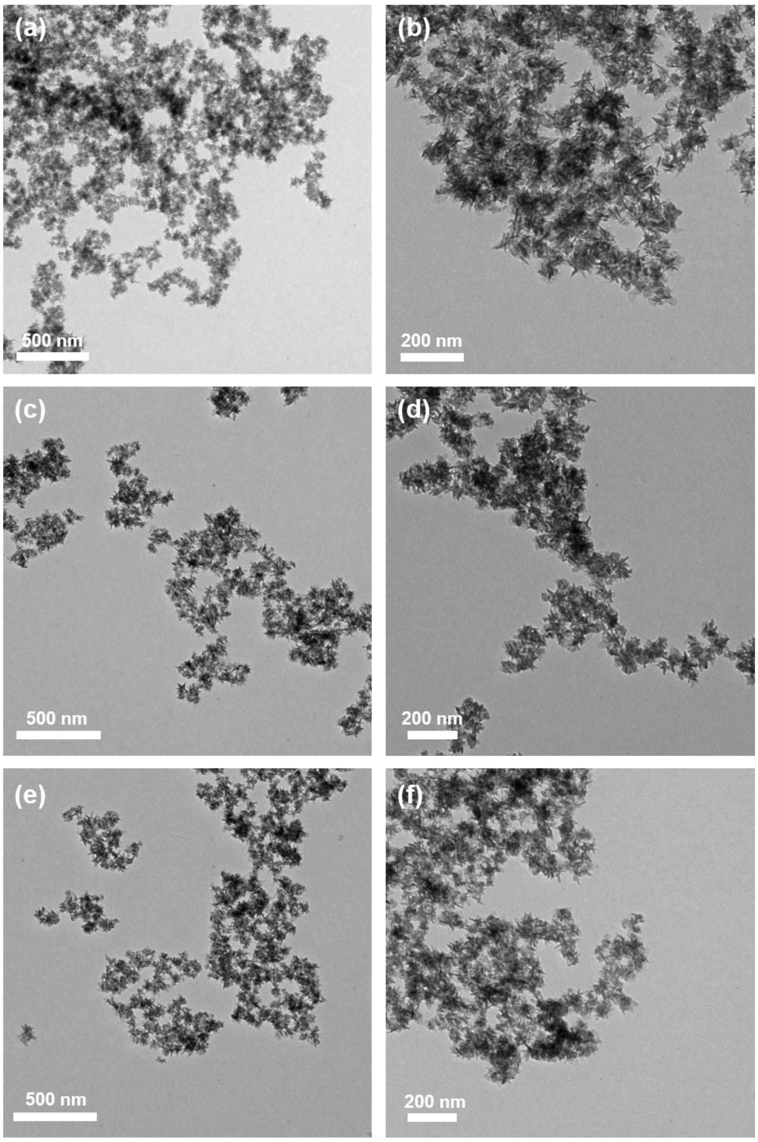

Supplementary Figure 49. The TEM images of (a,b) Zr-TCPE, (c,d) Zr-TCPE-L_1, and (e,f) Zr-TCPE-L_2 constructed with deuterated H_4TCPE.

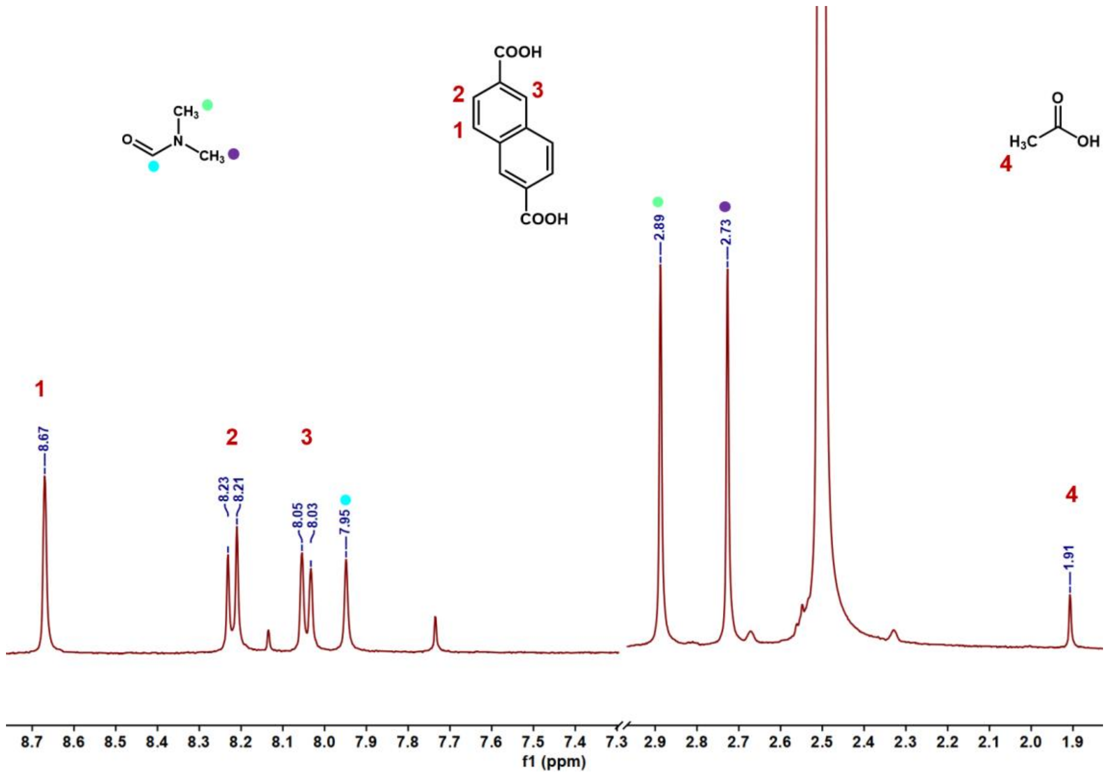

Supplementary Figure 50. The ^{1}H NMR spectroscopy of digested Zr-TCPE-L_1 constructed with deuterated H_4TCPE.

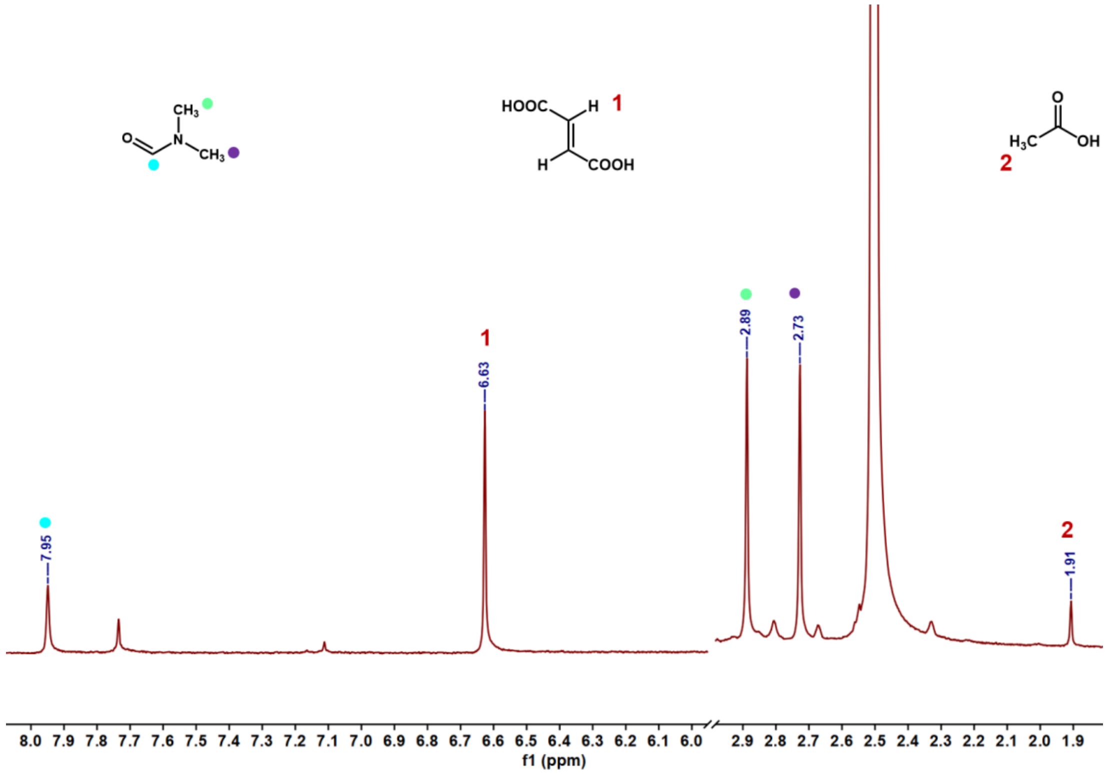

Supplementary Figure 51. The ^{1}H NMR spectroscopy of digested Zr-TCPE-L_2 constructed with deuterated H_4TCPE.

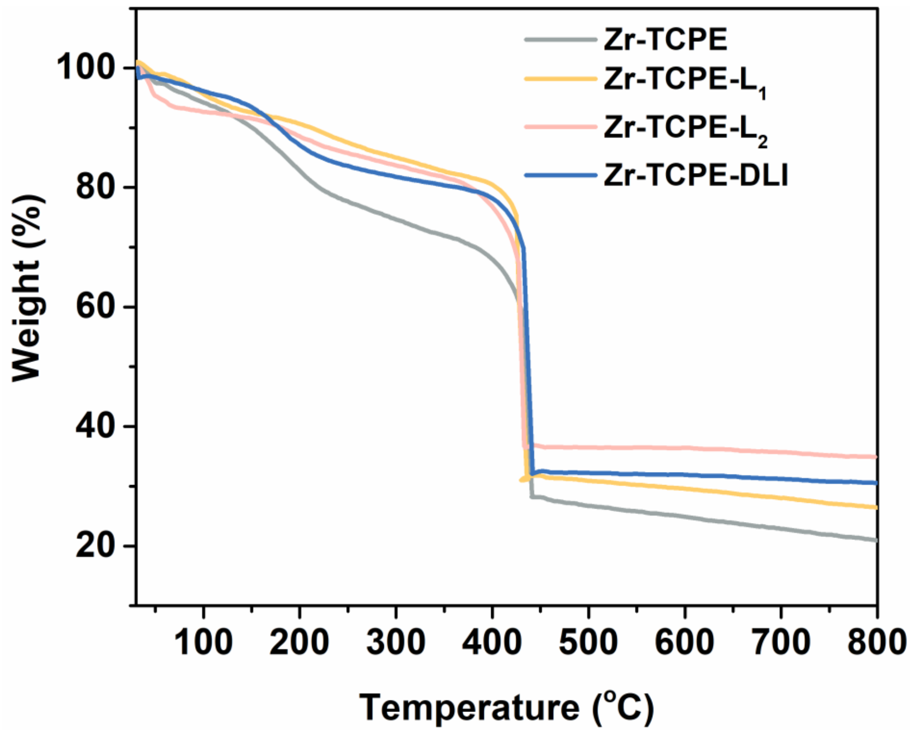

Supplementary Figure 52. TGA plots of Zr-TCPE, Zr-TCPE-L_1, Zr-TCPE- L_2, and Zr-TCPE-DLI. Experiment condition: temperature ramp from 30 ^oC to 800 ^oC at 10 ^oC/min under O_2 flow.

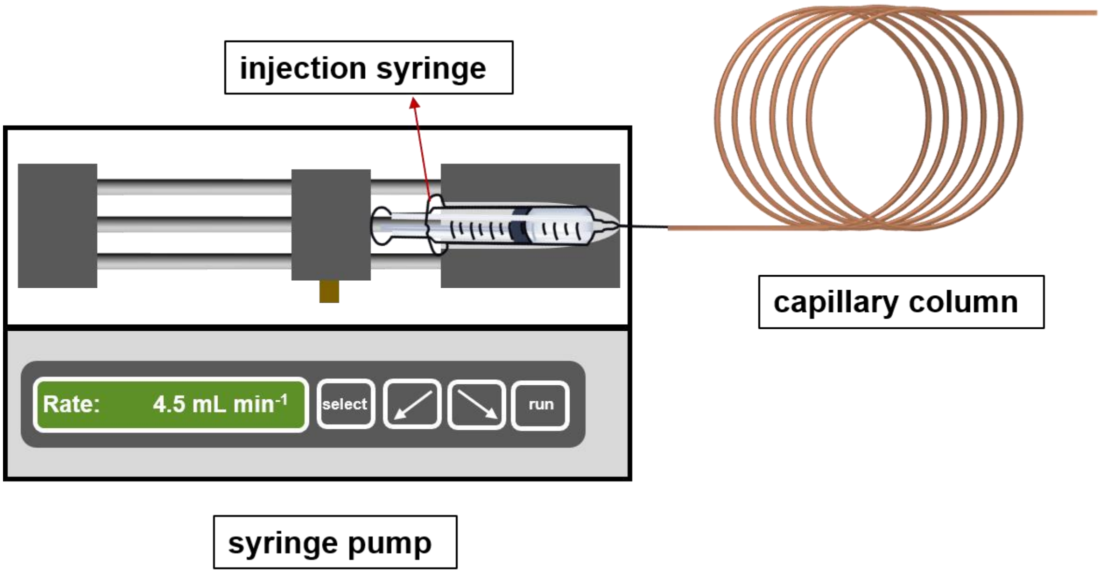

Supplementary Figure 53. The illustration of coating materials into the capillary column.

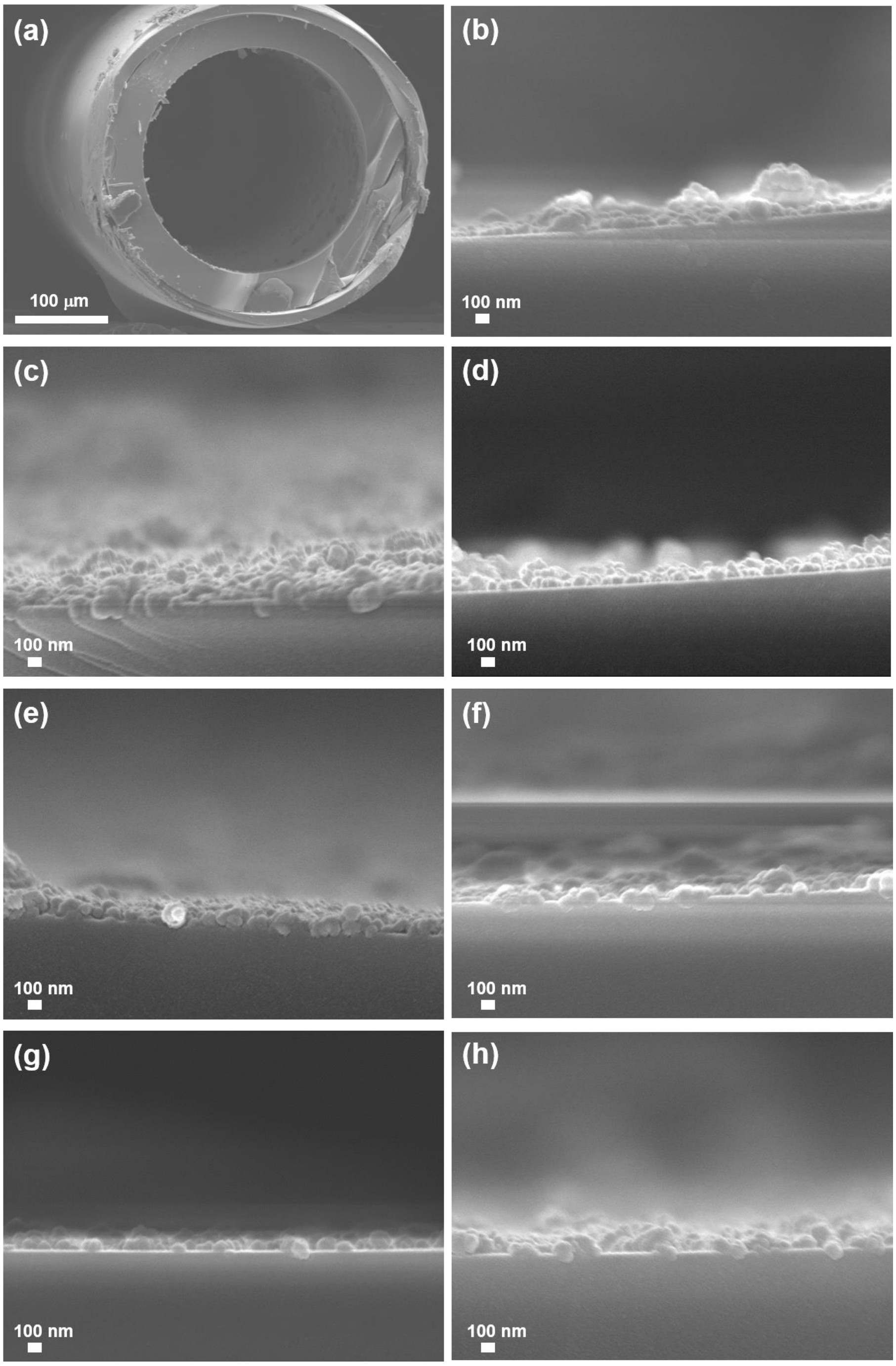

Supplementary Figure 54. The SEM images from the cross-view of capillary columns coated with(a) Zr-TCPE, (b) Zr-TCPE-H, (c) Zr-TCPE-L_1, (d) Zr-TCPE-L_1-H, (e) Zr-TCPE-L_2, (f) Zr-TCPE-L_2-H, (g) Zr-TCPE-DLI and (h) Zr-TCPE-DLI-H.

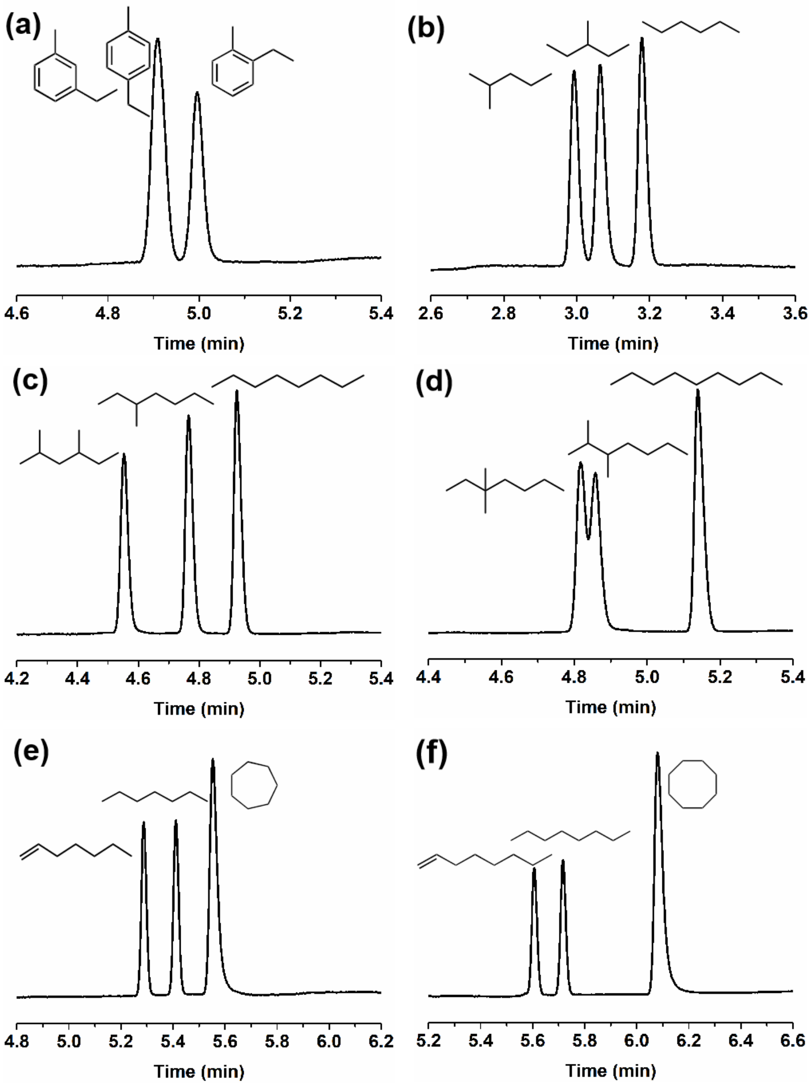

Supplementary Figure 55. Gas chromatograms on the Zr-TCPE coated capillary column (15 m long × 0.25 mm i.d.) for the separation of (a) ethylbenzene isomers using a temperature program of 120 °C for 1 min, and then 20 °C·min^{-1} to 250 °C; (b) C_6 alkane isomers using a temperature program of 80 °C for 1 min, and then 20 °C·min^{-1} to 250 °C; (c) C_8 alkane isomers using a temperature program of 100 °C for 1 min, and then 20 °C·min^{-1} to 250 °C; (d) C_9 alkane isomers using a temperature program of 120 °C for 1 min, and then 20 °C·min^{-1} to 250 °C; (e) mixture of 1-heptene, n-heptane, and cycloheptane using a temperature program of 100 °C for 1 min, and then 20 °C·min^{-1} to 250 °C; (f) mixture of 1-octene, n-octane, and cyclooctane using a temperature program of 100 °C for 1 min, and then 20 °C·min^{-1} to 250 °C.

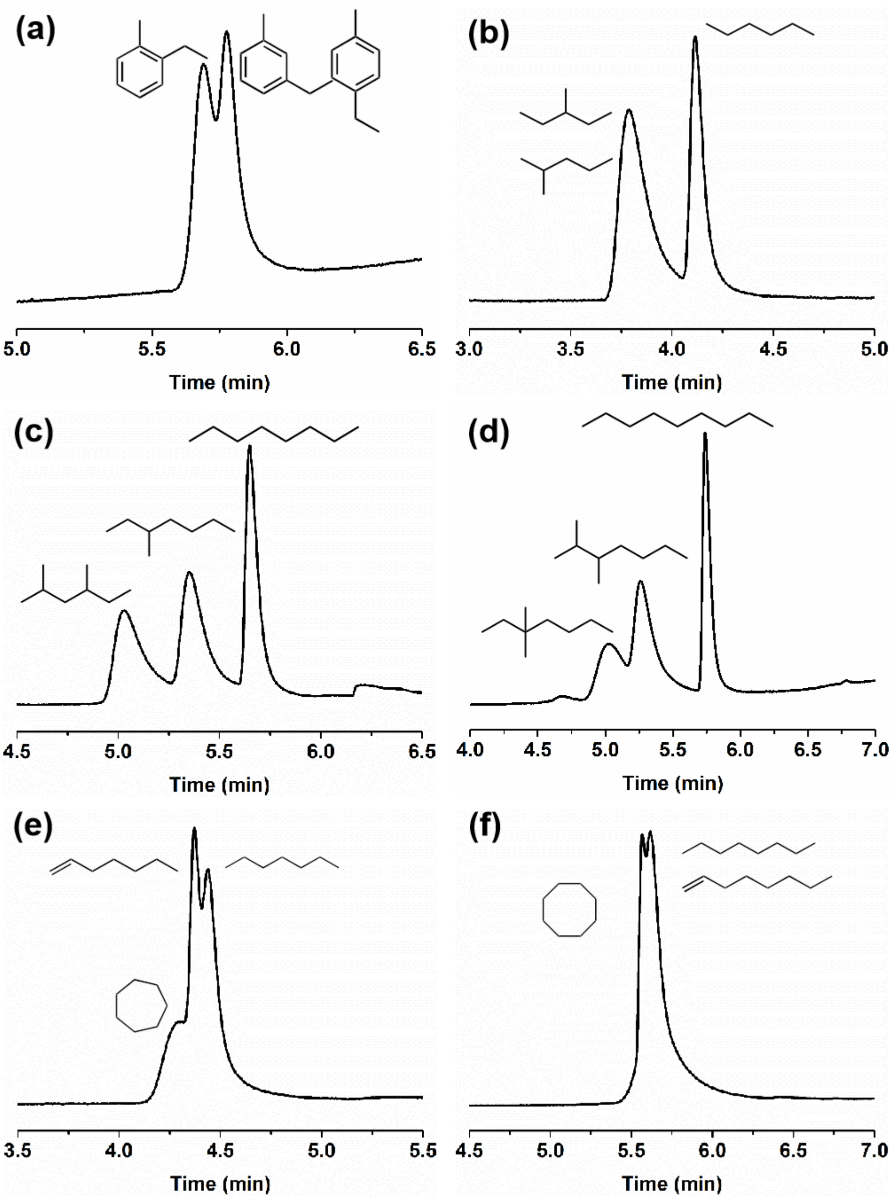

Supplementary Figure 56. Gas chromatogram on the Zr-TCPE-H coated capillary column (15 m long × 0.25 mm i.d.) for the separation of (a) ethylbenzene isomers using a temperature program of 120 °C for 1 min, and then 20 °C·min^{-1} to 250 °C; (b) C_6 alkane isomers using a temperature program of 80 °C for 1 min, and then 20 °C·min^{-1} to 250 °C; (c) C_8 alkane isomers using a temperature program of 100 °C for 1 min, and then 20 °C·min^{-1} to 250 °C; (d) C_9 alkane isomers using a temperature program of 120 °C for 1 min, and then 20 °C·min^{-1} to 250 °C; (e) mixture of 1-heptene, n-heptane, and cycloheptane using a temperature program of 100 °C for 1 min, and then 20 °C·min^{-1} to 250 °C; (f) mixture of 1-octene, n-octane, and cyclooctane using a temperature program of 100 °C for 1 min, and then 20 °C·min^{-1} to 250 °C.

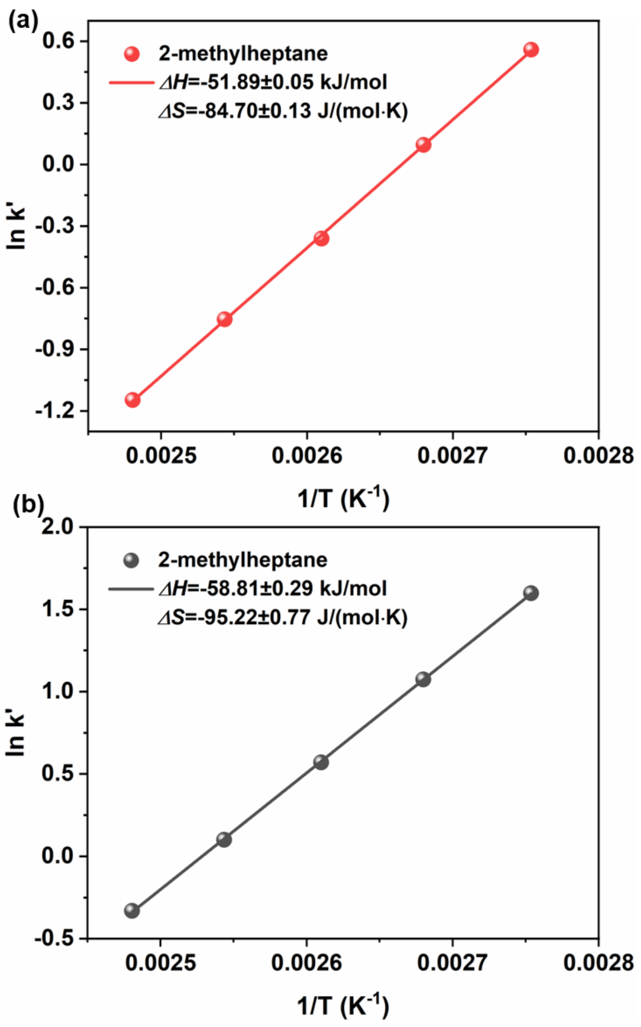

Supplementary Figure 57. The van't Hoff plots of 2-methylpentane on (a) Zr-TCPE and (b) Zr-TCPE-H coated columns. The thermodynamic adsorption enthalpy (*ΔH*) of 2-methylpentane on Zr-TCPE and Zr-TCPE-H coated column was -51.89 kJ/mol and -58.81 kJ/mol, respectively.

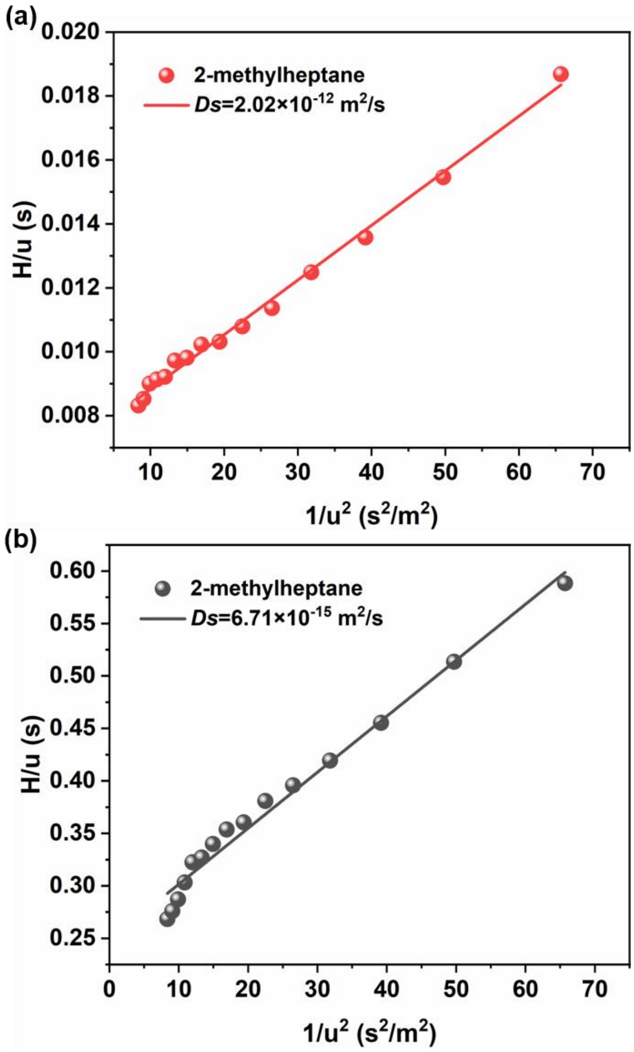

Supplementary Figure 58. The Golay plots of 2-methylpentane on (a) Zr-TCPE and (b) Zr-TCPE-H coated columns. The test temperature was 393.15 K. The kinetic diffusion constant (*Ds*) of 2-methylpentane on Zr-TCPE and Zr-TCPE-H coated column was 2.02×10^{-12} m^2/s and 6.71×10^{-15} m^2/s, respectively. The calculated resistance to mass transfer coefficient (C_s) of 2-methylpentane on Zr-TCPE and Zr-TCPE-H coated column was 7.10×10^{-4} and 0.25, respectively.

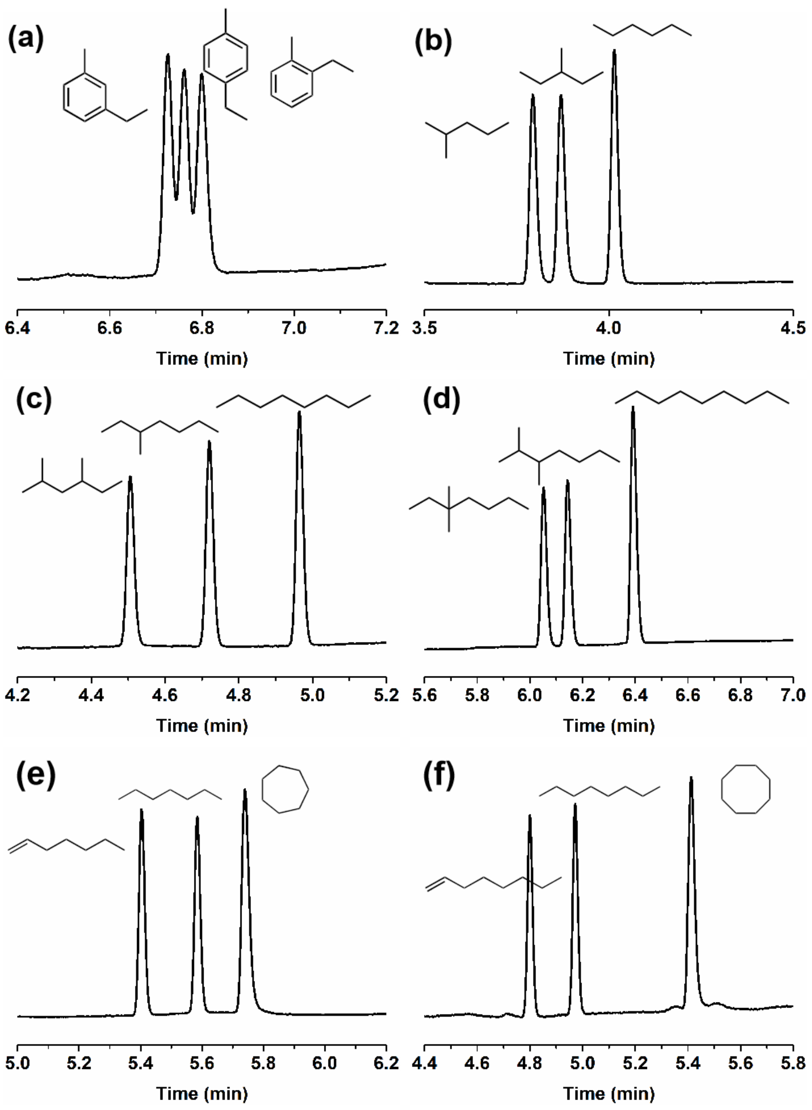

Supplementary Figure 59. Gas chromatogram on the Zr-TCPE-L_2 coated capillary column (15 m long × 0.25 mm i.d.) for the separation of (a) ethylbenzene isomers using a temperature program of 100 °C for 1 min, and then 20 °C·min^{-1} to 250 °C; (b) C_6 alkane isomers using a temperature program of 80 °C for 1 min, and then 20 °C·min^{-1} to 250 °C; (c) C_8 alkane isomers using a temperature program of 120 °C for 1 min, and then 20 °C·min^{-1} to 250 °C; (d) C_9 alkane isomers using a temperature program of 140 °C for 1 min, and then 20 °C·min^{-1} to 250 °C; (e) mixture of 1-heptene, n-heptane, and cycloheptane using a temperature program of 80 °C for 1 min, and then 20 °C·min^{-1} to 250 °C; (f) mixture of 1-octene, n-octane, and cyclooctane using a temperature program of 100 °C for 1 min, and then 20 °C·min^{-1} to 250 °C.

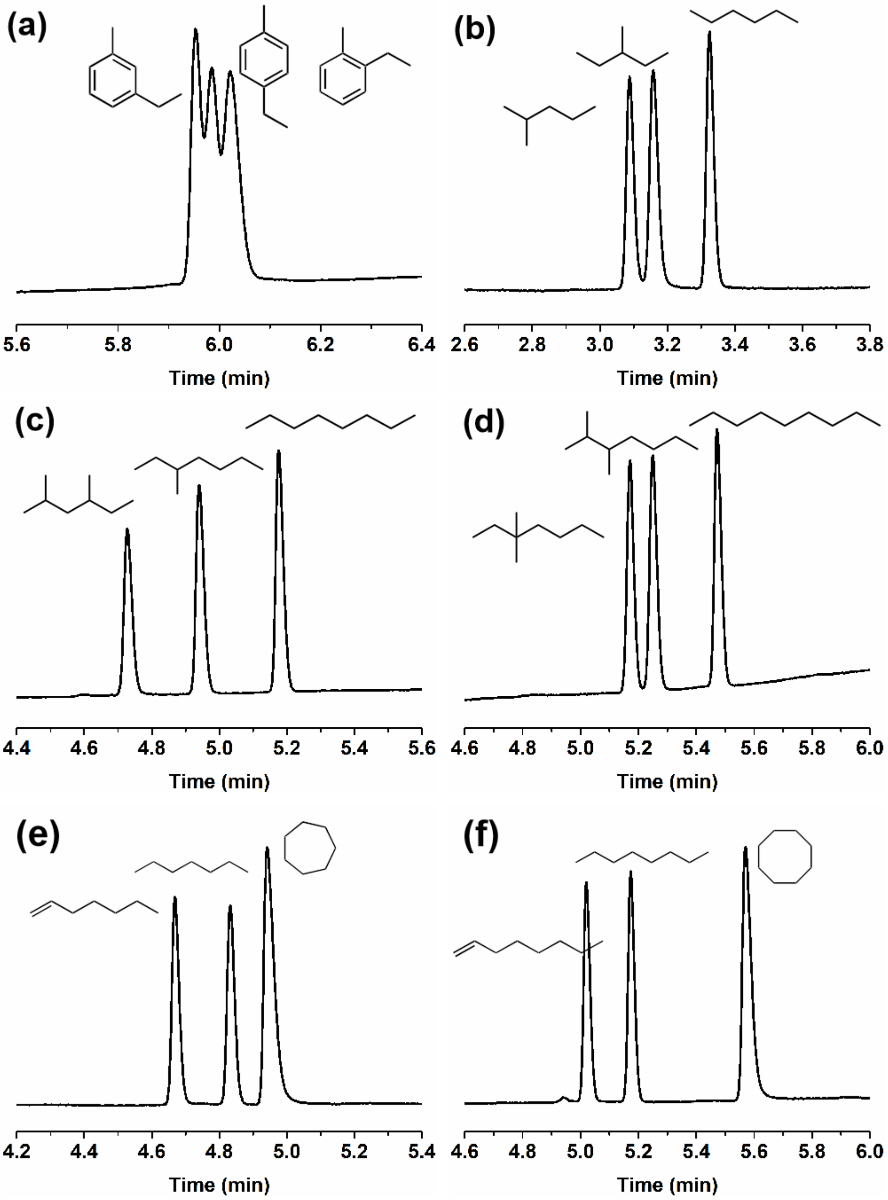

Supplementary Figure 60. Gas chromatogram on the Zr-TCPE-L_2-H coated capillary column (15 m long × 0.25 mm i.d.) for the separation of (a) ethylbenzene isomers using a temperature program of 100 °C for 1 min, and then 20 °C·min^{-1} to 250 °C; (b) C_6 alkane isomers using a temperature program of 80 °C for 1 min, and then 20 °C·min^{-1} to 250 °C; (c) C_8 alkane isomers using a temperature program of 100 °C for 1 min, and then 20 °C·min^{-1} to 250 °C; (d) C_9 alkane isomers using a temperature program of 120 °C for 1 min, and then 20 °C·min^{-1} to 250 °C; (e) mixture of 1-heptene, n-heptane, and cycloheptane using a temperature program of 80 °C for 1 min, and then 20 °C·min^{-1} to 250 °C; (f) mixture of 1-octene, n-octane, and cyclooctane using a temperature program of 100 °C for 1 min, and then 20 °C·min^{-1} to 250 °C.

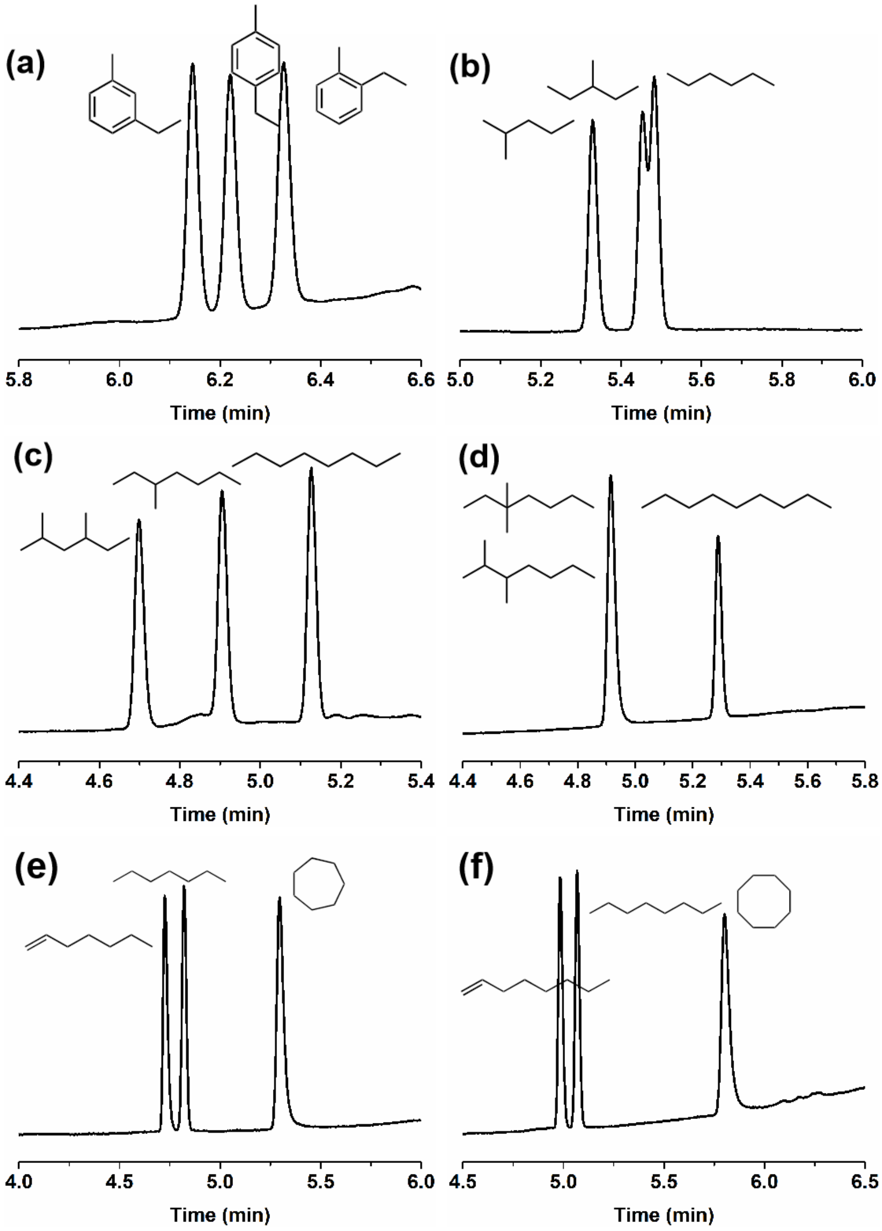

Supplementary Figure 61. Gas chromatogram on the Zr-TCPE-L_1 coated capillary column (15 m long × 0.25 mm i.d.) for the separation of (a) ethylbenzene isomers using a temperature program of 100 °C for 1 min, and then 20 °C·min^{-1} to 250 °C; (b) C_6 alkane isomers using a temperature program of 80 °C for 1 min, and then 20 °C·min^{-1} to 250 °C; (c) C_8 alkane isomers using a temperature program of 140 °C for 1 min, and then 20 °C·min^{-1} to 250 °C; (d) C_9 alkane isomers using a temperature program of 160 °C for 1 min, and then 20 °C·min^{-1} to 250 °C; (e) mixture of 1-heptene, n-heptane, and cycloheptane using a temperature program of 120 °C for 1 min, and then 20 °C·min^{-1} to 250 °C; (f) mixture of 1-octene, n-octane, and cyclooctane using a temperature program of 140 °C for 1 min, and then 20 °C·min^{-1} to 250 °C.

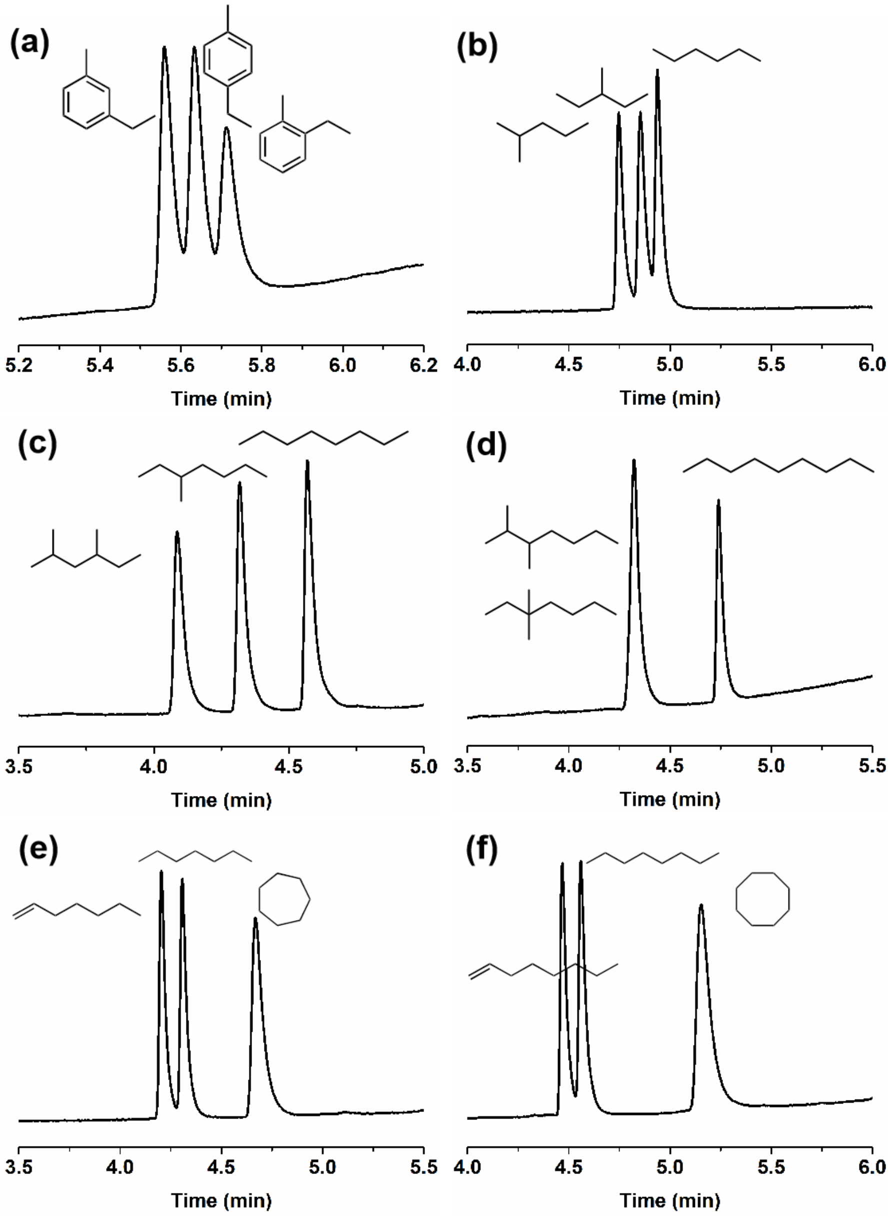

Supplementary Figure 62. Gas chromatogram on the Zr-TCPE-L_1-H coated capillary column (15 m long × 0.25 mm i.d.) for the separation of (a) ethylbenzene isomers using a temperature program of 140 °C for 1 min, and then 20 °C·min^{-1} to 250 °C; (b) C_6 alkane isomers using a temperature program of 80 °C for 1 min, and then 20 °C·min^{-1} to 250 °C; (c) C_8 alkane isomers using a temperature program of 140 °C for 1 min, and then 20 °C·min^{-1} to 250 °C; (d) C_9 alkane isomers using a temperature program of 160 °C for 1 min, and then 20 °C·min^{-1} to 250 °C; (e) mixture of 1-heptene, n-heptane, and cycloheptane using a temperature program of 120 °C for 1 min, and then 20 °C·min^{-1} to 250 °C; (f) mixture of 1-octene, n-octane, and cyclooctane using a temperature program of 140 °C for 1 min, and then 20 °C·min^{-1} to 250 °C.

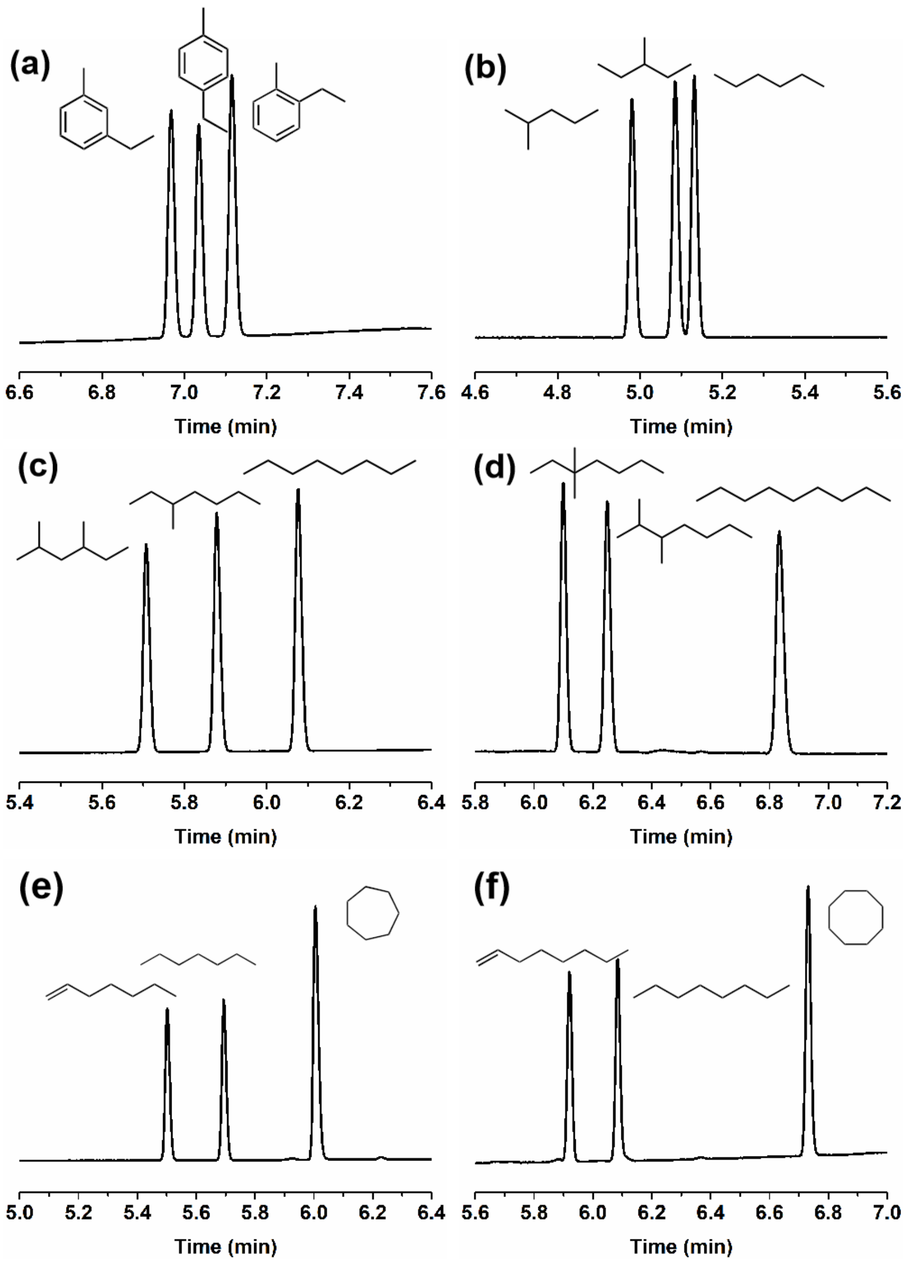

Supplementary Figure 63. Gas chromatogram on the Zr-TCPE-DLI coated capillary column (15 m long × 0.25 mm i.d.) for the separation of (a) ethylbenzene isomers using a temperature program of 120 °C for 1 min, and then 20 °C·min^{-1} to 250 °C; (b) C_6 alkane isomers using a temperature program of 80 °C for 1 min, and then 20 °C·min^{-1} to 250 °C; (c) C_8 alkane isomers using a temperature program of 120 °C for 1 min, and then 20 °C·min^{-1} to 250 °C; (d) C_9 alkane isomers using a temperature program of 120 °C for 1 min, and then 20 °C·min^{-1} to 250 °C; (e) mixture of 1-heptene, n-heptane, and cycloheptane using a temperature program of 100 °C for 1 min, and then 20 °C·min^{-1} to 250 °C; (f) mixture of 1-octene, n-octane, and cyclooctane using a temperature program of 120 °C for 1 min, and then 20 °C·min^{-1} to 250 °C.

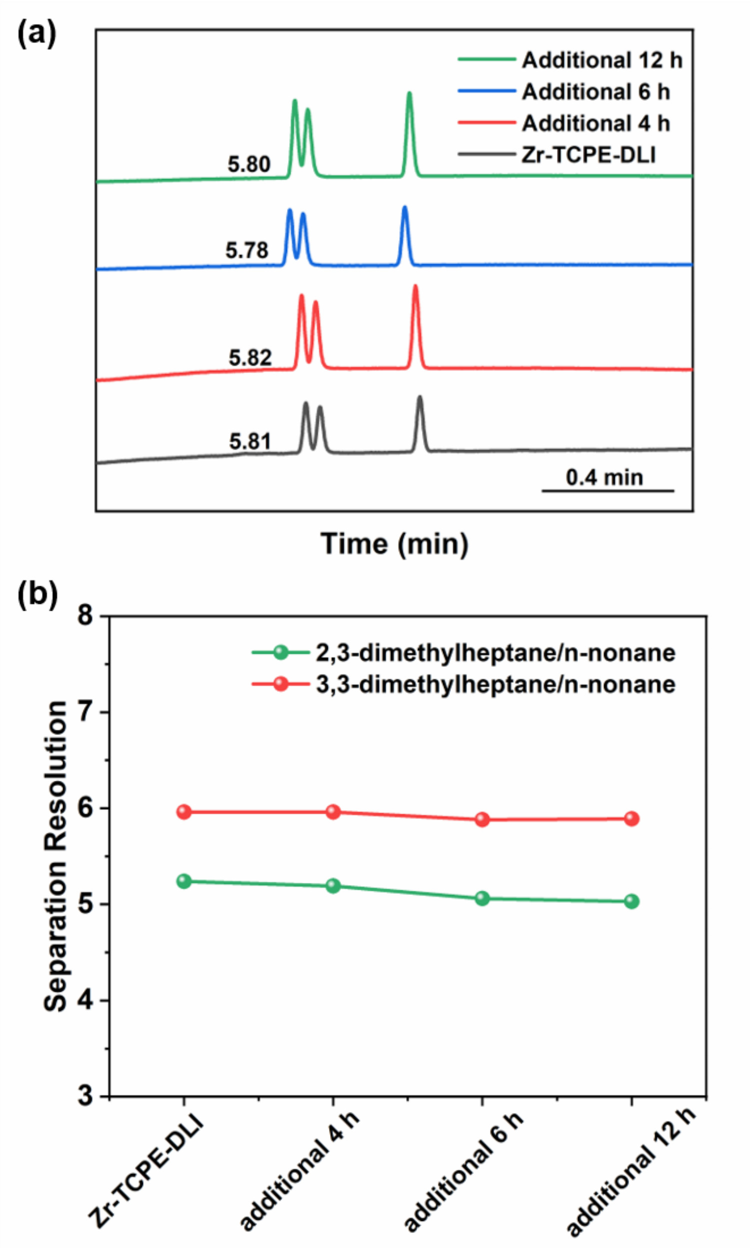

Supplementary Figure 64. (a) The gas chromatograms on Zr-TCPE-DLI coated column for the separation of nonane isomers with additional heating at 250 ^{o}C for 4 h, 6 h, and 12 h. (b) The separation resolution of nonane isomers on Zr-TCPE-DLI coated column with additional heating at 250 ^{o}C for 4 h, 6 h, and 12 h.

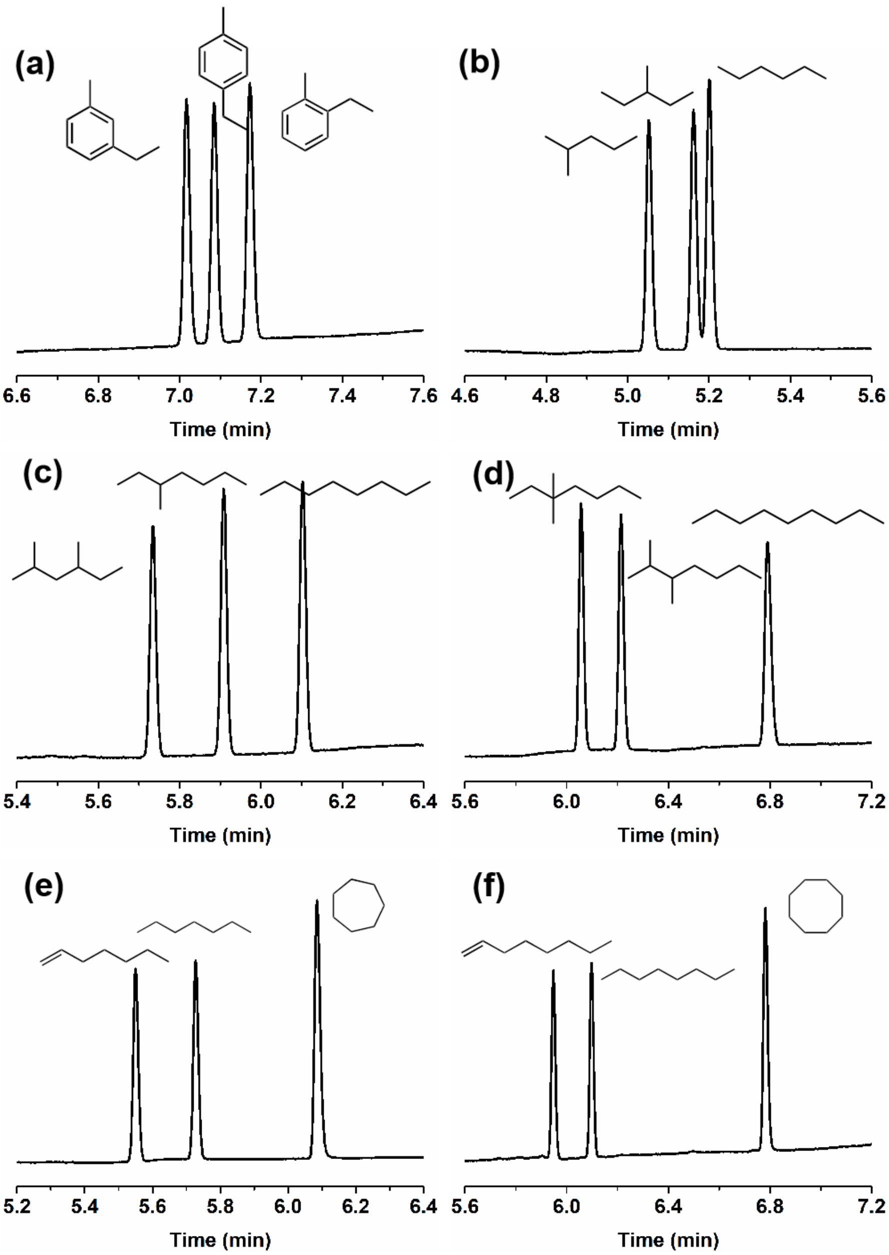

Supplementary Figure 65. Gas chromatogram on the Zr-TCPE-DLI-H coated capillary column (15 m long × 0.25 mm i.d.) for the separation of (a) ethylbenzene isomers using a temperature program of 120 °C for 1 min, and then 20 °C·min^{-1} to 250 °C; (b) C_6 alkane isomers using a temperature program of 80 °C for 1 min, and then 20 °C·min^{-1} to 250 °C; (c) C_8 alkane isomers using a temperature program of 120 °C for 1 min, and then 20 °C·min^{-1} to 250 °C; (d) C_9 alkane isomers using a temperature program of 120 °C for 1 min, and then 20 °C·min^{-1} to 250 °C; (e) mixture of 1-heptene, n-heptane, and cycloheptane using a temperature program of 100 °C for 1 min, and then 20 °C·min^{-1} to 250 °C; (f) mixture of 1-octene, n-octane, and cyclooctane using a temperature program of 120 °C for 1 min, and then 20 °C·min^{-1} to 250 °C.

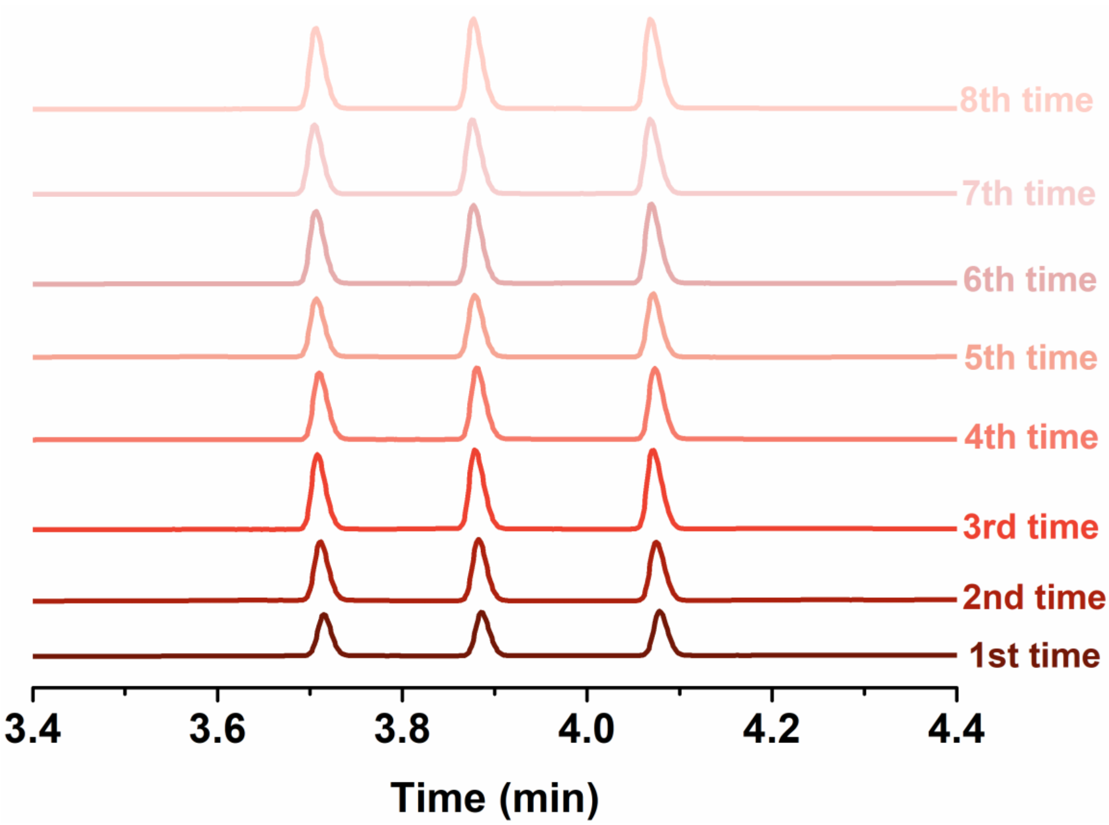

Supplementary Figure 66. The repeatability of Zr-TCPE-DLI-H coated capillary column for the separation of C_8 alkane isomers 8 times continuously using a temperature program of 160 ^{o}C for 1 min, and then 20 $^{o}C \cdot min^{-1}$ to 250 ^{o}C.

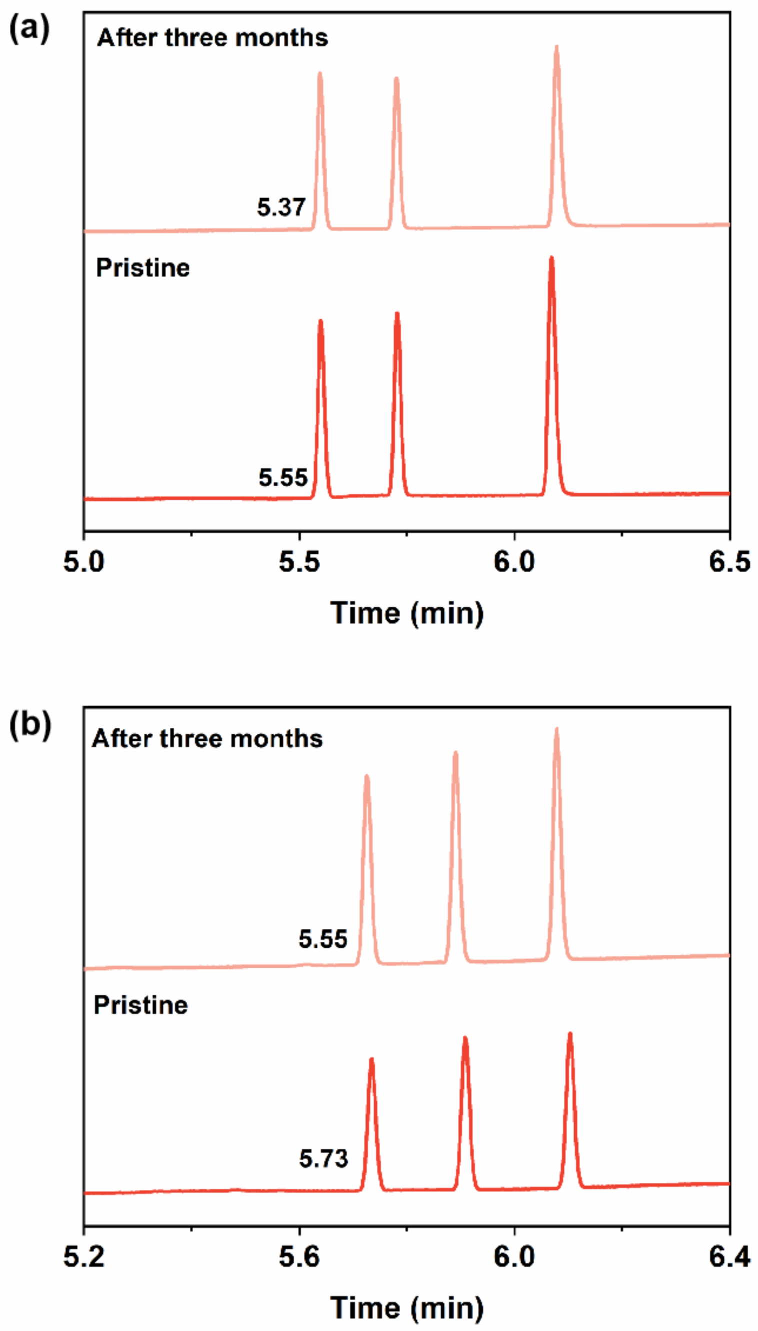

Supplementary Figure 67. The separation stability of Zr-TCPE-DLI-H coated capillary column for the separation of (a) C_7 alkene isomers and (b) C_8 alkane isomers before and after the storage for three months. The numbers on the left represent the elution time of the first peak. The separation ability and peak shape have no obvious changes. The forward shift of retention time resulted from the shortening of capillary column length due to unavoidable depletion.

Supplementary Tables

Supplementary Table 1. Crystallographic parameters of synthesized Zr-TCPE.

**Compound**	**Zr-TCPE**
Crystal system	Orthorhombic
Space group	*Cmmm*
Radiation	CuKα (l=1.54056 Å)
a/Å	17.5111
b/Å	30.1849
c/Å	12.1237
V/Å^3	6408.20
a	90.00000
b	90.00000
g	90.00000
R_{wp}/%	13.61
R_p/%	10.68
R_{Bragg}/%	2.77
GOF	3.98

Supplementary Table 2. Atomic parameters of Zr-TCPE.

Atom	x/a	y/b	z/c
O1	0.56037	0.61971	0.79012
O2	0.67488	0.61874	0.5577
C3	0.68413	0.66037	0.80988
C4	0.73336	0.70335	0.83599
C5	0.75656	0.69527	0.83985
H6	0.75226	0.64777	0.95985
C7	0.755	0.69553	0.83847
H8	0.72529	0.6694	0.79736
C9	0.59055	0.72943	0.51884
C10	0.66646	0.7544	0.90941
C11	0.62371	0.67245	0.83232
H12	0.40513	0.73503	1.45902
H13	0.22274	0.83402	1.20438
Zr14	0.38698	0.48315	0.36164
O15	0.45017	0.50124	0.21789
O16	0.26593	0.48604	0.29215
Zr17	0.46672	0.57749	0.44966
O18	0.50335	0.53726	0.32087
O19	0.27924	0.56993	0.56334
C20	0.68962	0.72665	0.85677

Supplementary Table 3. Main reflection parameters of Zr-TCPE.

(hkl)	2 Theta (degree)	d-spacing (nm)
(020)	5.85	1.51
(001)	7.29	1.21
(021)	9.35	0.94
(130)	10.13	0.87
(040)	11.72	0.75
(131)	12.49	0.71

Supplementary Table 4. Porosity parameters of Zr-TCPE from N_2 and Ar adsorption isotherms.

**Adsorbate**	**BET surface area (m^2/g)**	**Main pore size (Å)**	**Total pore volume (cm^3/g)**
N_2	934	9.4	0.67
Ar	955	8.7	0.64

Supplementary Table 5. Ligand ratios from ^{1}H NMR spetra of digested materials.

**Material**	**Ligand ratios**
Zr-TCPE	TCPE: AA= 1:0.5
Zr-TCPE- L_1	TCPE: L_1: AA= 1:0.5:0.03
Zr-TCPE- L_2	TCPE: L_2: AA=1:0.4:0.03
Zr-TCPE-DLI	TCPE: L_1: L_2: AA=1:0.5:0.1:0.03
Zr-TCPE-DLI’	TCPE: L_1: L_2: AA=1:0.3:0.3:0.04
Zr-TCPE-H	TCPE: AA= 1:0.3
Zr-TCPE-L_1-H	TCPE: L_1: AA=1:0.5:0.03
Zr-TCPE-L_2-H	TCPE: L_2: AA=1:0.3:0.06
Zr-TCPE-DLI-H	TCPE: L_1: L_2: AA=1:0.5:0.1:0.02
Zr-TCPE-DLI’-H	TCPE: L_1: L_2: AA=1:0.3:0.3:0.04

Supplementary Table 6. Separation resolution (R_S) for different isomers on different MOFs coated capillary columns.

Analytes	Zr-TCPE-H	Zr-TCPE-L_1-H	Zr-TCPE-L_2-H	Zr-TCPE-DLI-H
3-ethylbenzene/4-ethylbenzene	0.16	0.63	0.28	**1.33**
4-ethylbenzene/2-ethylbenzene	0.22	0.55	0.25	**1.60**
3-ethylbenzene/2-ethylbenzene	0.04	1.10	0.50	**2.77**
2-methylpentane/3-methylpentane	0.00	0.83	1.02	**2.34**
3-methylpentane/n-hexane	0.82	0.56	**1.96**	0.83
2-methylpentane/n-hexane	0.85	1.35	3.00	**3.22**
2,4-dimethylhexane/3-methylheptane	0.75	1.45	2.51	**3.23**
3-methylheptane/n-octane	0.97	1.50	2.89	**3.58**
2,4-dimethylhexane/n-octane	1.63	2.91	5.24	**6.70**
4,4-dimethyloctane/3,3-dimethyloctane	0.00	0.82	1.03	**2.35**
3,3-dimethyloctane/n-decane	1.58	2.75	2.45	**8.13**
4,4-dimethyloctane/n-decane	1.67	3.81	3.55	**10.11**
1-heptene/n-heptane	0.10	0.77	2.01	**3.50**
n-heptane/cycloheptane	0.49	1.90	1.12	**6.43**
1-heptene/cycloheptane	0.43	2.66	2.84	**9.64**
1-octene/n-octane	0.12	0.77	1.67	**2.97**
n-octane/cyclooctane	0.37	2.85	4.01	**12.11**
1-octene/cyclooctane	0.24	3.45	5.12	**16.35**

The highest value for each pair is marked as bold.

Reference

1. Yuan, S. et al. Linker Installation: Engineering Pore Environment with Precisely Placed Functionalities in Zirconium MOFs. *J. Am. Chem. Soc.* **138**, 8912-8919 (2016).
2. Robison, L. et al. Designing Porous Materials to Resist Compression: Mechanical Reinforcement of a Zr-MOF with Structural Linkers. *Chem. Mater.* **32**, 3545-3552 (2020).
3. Liang, L. et al. Non-Interpenetrated Single-Crystal Covalent Organic Frameworks. *Angew. Chem. Int. Ed.* **59**, 17991-17995 (2020).
4. Xie, S. et al. Fluorogenic Ag^+-Tetrazolate Aggregation Enables Efficient Fluorescent Biological Silver Staining. *Angew. Chem. Int. Ed.* **57**, 5750-5753 (2018).
5. Shustova, N.B., McCarthy, B.D. & Dincă, M. Turn-On Fluorescence in Tetraphenylethylene-Based Metal-Organic Frameworks: An Alternative to Aggregation-Induced Emission. *J. Am. Chem. Soc.* **133**, 20126-20129 (2011).
